# Novel Cancer Chemotherapy Hits by Molecular Topology: Dual Akt and Beta-Catenin Inhibitors

**DOI:** 10.1371/journal.pone.0124244

**Published:** 2015-04-24

**Authors:** Riccardo Zanni, Maria Galvez-Llompart, Cecilia Morell, Nieves Rodríguez-Henche, Inés Díaz-Laviada, Maria Carmen Recio-Iglesias, Ramon Garcia-Domenech, Jorge Galvez

**Affiliations:** 1 Department of Physical Chemistry, School of Pharmacy, University of Valencia, Valencia, Spain; 2 Department of System Biology, Biochemistry and Molecular Biology Unit, School of Medicine, University of Alcala, Alcalá de Henares, Spain; 3 Department of Pharmacology, School of Pharmacy, University of Valencia, Valencia, Spain; Taipei Medical University, TAIWAN

## Abstract

**Background and Purpose:**

Colorectal and prostate cancers are two of the most common types and cause of a high rate of deaths worldwide. Therefore, any strategy to stop or at least slacken the development and progression of malignant cells is an important therapeutic choice. The aim of the present work is the identification of novel cancer chemotherapy agents. Nowadays, many different drug discovery approaches are available, but this paper focuses on Molecular Topology, which has already demonstrated its extraordinary efficacy in this field, particularly in the identification of new *hit* and *lead* compounds against cancer. This methodology uses the graph theoretical formalism to numerically characterize molecular structures through the so called topological indices. Once obtained a specific framework, it allows the construction of complex mathematical models that can be used to predict physical, chemical or biological properties of compounds. In addition, Molecular Topology is highly efficient in selecting and designing new *hit* and *lead* drugs. According to the aforementioned, Molecular Topology has been applied here for the construction of specific Akt/mTOR and β-catenin inhibition mathematical models in order to identify and select novel antitumor agents.

**Experimental Approach:**

Based on the results obtained by the selected mathematical models, six novel potential inhibitors of the Akt/mTOR and β-catenin pathways were identified. These compounds were then tested *in vitro* to confirm their biological activity.

**Conclusion and Implications:**

Five of the selected compounds, CAS n° 256378-54-8 (Inhibitor n°1), 663203-38-1 (Inhibitor n°2), 247079-73-8 (Inhibitor n°3), 689769-86-6 (Inhibitor n°4) and 431925-096 (Inhibitor n°6) gave positive responses and resulted to be active for Akt/mTOR and/or β-catenin inhibition. This study confirms once again the Molecular Topology’s reliability and efficacy to find out novel drugs in the field of cancer.

## Introduction

The US-National Institute of Health estimated the direct cost on oncology care to be $89 billion in 2007 [1]. Surgery, hospitalization, physician visits, imaging, chemotherapy, radiation and biologic therapy are derived costs from oncology care [2]. In 2014, approximately 18% of US gross domestic product has been spent on healthcare and 5% of that keeps being for cancer care [3]. Ten to fifteen percent of total spending on oncology care is linked to cancer drugs [2]. Therefore, developing quality cost-saving strategies for cancer care is an imperative [3].

There are many approaches to fight cancer ranging from chemoprevention (strategy of blocking or slowing the onset of premalignant tumors with relatively nontoxic chemical substances [4]) to chemotherapy, radiotherapy or finally surgical oncology.

Ones of the most common and deadly forms of cancer, colorectal cancer (CRC) and prostate cancer (PtC) were selected as main targets for this study. In order to give an idea of the importance of these forms of cancer, a brief description of their incidence and burden is following.

Worldwide, about 2.1 million people were diagnosed with CRC in 2008, placing it second among the most frequent cancer in women and third in men [5]. Moreover, it is the third most common cause of cancer death worldwide, with more than 600,000 deaths per year [6]. The prevalence of CRC is expected to increase significantly in most developed countries as a result of the growing population belonging to the elderly, since the incidence of CRC increases with age [5].

On the other hand, prostate cancer is the second most common malignancy in men, with more than 900,000 newly diagnosed cancer cases and about 260,000 cancer deaths in 2008 [7]. The incidence of this deadly disease has significantly increased in recent years because of the widespread screening for prostate-specific antigen (PSA), which allows early detection of tumors that otherwise, might remain undetected. In the USA, 90% of patients with prostate cancer who presented localized lesions at the diagnosis, usually have a good prognosis after treatment. However, by 5 years, nearly 30% of treated patients exhibit a rise in PSA levels and evidence of recurrent disease [7].

Considering what has been said up to now, it is not difficult to understand the importance of trying to reduce the incidence and progression of CRC and PtC, so to prevent the inevitable health-spending burden that their treatment and follow-up of patients would imply. Again, the main objective of this work consists in try to discover novel drugs for cancer treatment.

**Fig 1:**
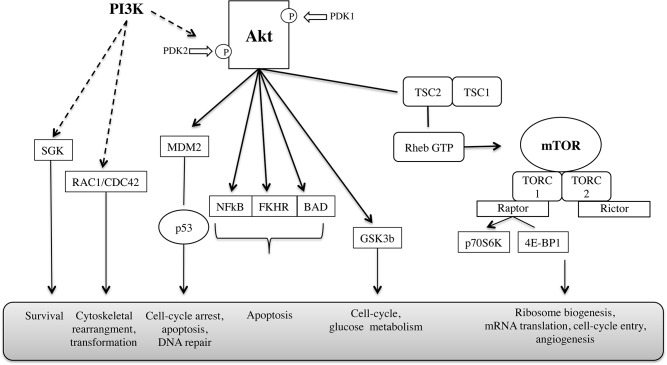
PI3K/Akt/mTOR pathway related to cancer onset and progression [11].

There are many targets involved in CRC and PtC which could be potentially interesting in order to select novel chemotherapeutic compounds. This work focused on two fundamental signaling pathways: PI3K/Akt/mTOR and Wnt/β-catenin (Fig 1).

### The PI3K/Akt/mTOR Pathway

Phosphatidylinositol-3-kinase (PI3K) is a key enzyme in the control of cell growth and proliferation. The most common form of this enzyme is activated by the actions of growth factors receptors. By forming triply phosphorylated inositols attracts Akt which becomes phosphorylated by phosphorinositide-dependent kinase (PDKs). Akt then proceeds to phosphorylate a variety of substrates, including the mammalian target of rapamycin (mTOR) regulating cell proliferation, survival and size.

Several studies have shown changes in Akt activity or expression in human precancerous tissues in precancerous prostatic intraepithelial neoplasia and neoplastic colonic epithelium [8]. Moreover, the PI3K/Akt and mTOR signaling pathways are demonstrated to be hyper-activated signaling pathways in CRC and PtC cancers [8–15]. Therefore, these signaling pathways are potent targets for inducing cancer cell death [12].

### The Wnt/β-Catenin Pathway

In the absence of Wnt, β-catenin is associated with the multi-protein β-catenin destruction complex that includes constitutively active glycogen synthase kinase 3 (GSK3). GSK3 phosphorylates β-catenin, triggering its degradation through the ubiquitin-proteasome pathway. The presence of Wnt, induces β-catenin dissociation of the degradation complex and its translocation into the nucleus, where it binds to T-cell factor (TCF) transcription factors regulating the expression of many genes including Cyclin D1 (CycD1).

Deregulation of Wnt/β-catenin signaling is a hallmark of the majority of sporadic forms of colorectal cancer and results in increased stability of the protein β-catenin [16]. There are several reasons for targeting the Wnt/β-catenin pathway in CRC and PtC. Approximately 90% of colorectal cancers (CRCs) has mutations on the Wnt/β-catenin (canonical pathway). Those are mainly found in the adenomatous polyposis coli (APC) and β-catenin genes and both lead to pathway activation, although other pathway components can also harbor mutations [16]. Accumulation of β-catenin in the nucleus can be detected in > 80% of CRC tumors [16]. Moreover, high levels of nuclear β-catenin have been correlated with a poor prognosis in CRC patients [16].

In PtC, it has been widely demonstrated how androgen receptor (AR) is the major therapeutic target. However, targeting AR alone can result in drug resistance and disease recurrence. Simultaneous targeting of multiple pathways could in principle be an effective approach to treating prostate cancer [17]. Growing evidence indicates that the canonical Wnt/β-catenin pathway plays an important role in prostate tumor-genesis [18]. Especially because AR binds β-catenin directly to stimulate AR-mediated gene transcription, and importantly, the AR gene itself is a transcriptional target of β-catenin [17]. So, the inhibition of both the AR and β-catenin–signaling pathways that are often unregulated in prostate cancer may represent an effective way for PtC treatment.

### Molecular Topology in Cancer

Once it is clear the importance to find new chemotherapy agents against CRC and PtC, the question to be asked is: how could it be done? In the field of Computer Aided Drug Design (CADD) and Quantitative structure–activity relationship (QSAR), the possibilities are many, but the results obtained are seldom satisfactory, especially in the field of cancer. Since, the Drug Design and Molecular Topology Research Unit at the University of Valencia (Spain), has a consolidated background of achievements in cancer drug discovery [19–26], including several patents [27–29], thanks to a computational methodology based on Molecular Topology. In addition to the identification of novel active molecules in PtC [26], our research unit also succeeded in finding new molecules active on the initial stages of colorectal cancer. Moreover, our team has obtained significant results in IBD (inflammatory bowel disease) treatment, by the identification of several compounds inhibiting inflammation mediators like IL-6, NF-ĸβ and COX-2 [30–34].

The use of QSAR with topological indices as regression variables has proved to be an excellent approach for a fast and accurate prediction of many physicochemical and biological properties [35–37]. Molecular Topology (MT) can be defined as a part of mathematical chemistry consisting of the topological description of molecular structures. Such description deals basically with the connectivity of the atoms forming the molecule that must yield numerical values which are invariant under deformation of the structure. Features such as bond lengths, dihedral angles, energies or any other sort of physical or geometrical magnitudes are not being considered in this scenario. Molecular descriptors are ‘numbers that characterize a specific aspect of a molecule’ [38]. That is, they are numbers containing information derived from the structural representation of the molecule.

MT’s singularity lies in the following reasons:

- Molecular structure is described within a mathematical framework [39].
- It may find out new drugs, either by screening compound’s databases or by designing novel compounds following the reverse process (properties → structure).
- It can be easily computerized.

The use of MT as a tool for the design and selection of new drugs has been our team’s task since the early 1980’s [38–42].

## Materials and Methods

### Molecular Topology Model

**Fig 2:**
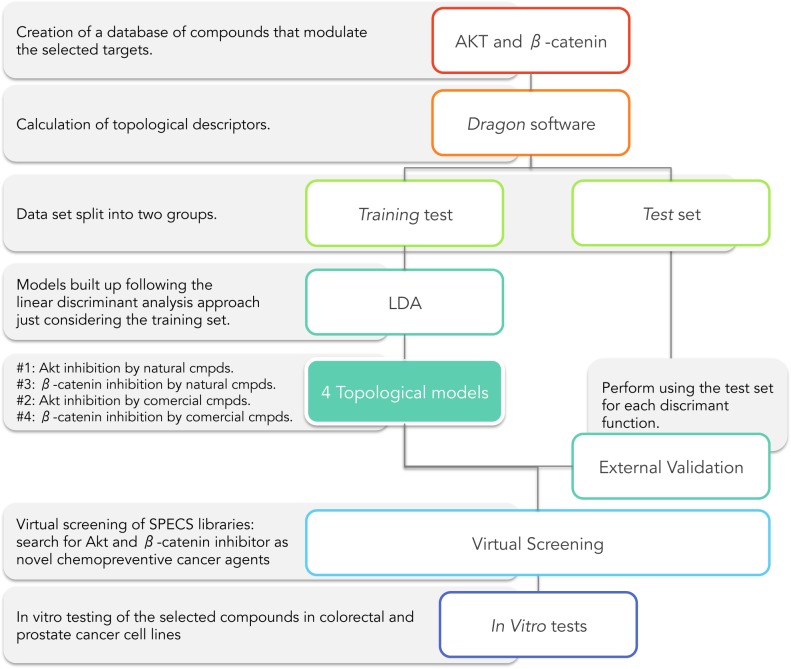
Scheme of Akt and β-catenin inhibitors research through Molecular Topology by virtual screening on SPECS databases.

Obtaining a MT-QSAR model for the identification of novel cancer chemotherapeutic agents consists of the following steps (Fig 2):

- Step I: Creating an in-house database of compounds active on the selected targets (Akt and β-catenin).
- Step II: Calculation of topological descriptors using Dragon software [43].
- Step III: Splitting data set into two groups: *training* and *test set*.
- Step IV: Models were built up following the linear discriminant analysis (LDA) approachment just considering the *training set*. The model’ labels were:
  - Model #1: Akt inhibition model for natural compounds.
  - Model #2: Akt inhibition model for comercial compounds.
  - Model #3: β-catenin inhibition model for natural compounds.
  - Model #4: β-catenin inhibition model for comercial compounds.
- Step V: Development of an external model validation using the *test set* for each discriminant function.
- Step VI: Carrying out a virtual screening in SPECS libraries. Search for potential Akt and β-catenin inhibitors as novel chemotherapeutics cancer agents.
- Step VII: *In vitro* testing of the selected compounds in colorectal and prostate cancer cell lines.

### Compound analysis

#### Model #1: Akt inhibition for natural compounds

The QSAR model was developed from a *training set* composed by 155 compounds (32 actives and 123 inactives) with heterogeneous molecular structures. This model was then tested against a set of 39 compounds consisting of 8 natural Akt inhibitors and 31 Akt-inactive compounds.

While we are confident that the active group compounds have been tested and exhibit pharmacological activity against Akt, regarding the inactive group compounds we don’t know for sure if they have been tested against such pharmacological activity. However, given that the number of compounds in the inactive set is significantly large, the risk of misclassification is negligible.

All compounds were obtained from different sources [44–80] and from a database named MicroSource Pure Natural Products Collection [81].

#### Model #2: Akt inhibition for comercial compounds

The QSAR model was developed from a *training set* composed by 647 compounds (20 actives and 627 inactive) with significant structural heterogeneity. External validation of this model was performed with a set of 205 compounds consisting of 6 active on Akt inhibition and 199 inactive.

As mentioned above, whereas we cannot ensure inactivity for all compounds belonging to the inactive set, the risk of false inactive is very low.

All compounds were obtained from Akt Selleckchem database [82], literature [83–97] and MicroSource US Drugs Collection [98].

#### Model #3: β-catenin inhibition for natural compounds

The QSAR model was developed from a structurally heterogeneous *training set* made of 153 compounds (50 actives and 103 inactives). This model was validated against a set of 27 compounds consisting of 10 natural β-catenin inhibitors and 17 17 inactive.

All compounds were obtained from literature [99–159] and from a database named MicroSource Pure Natural Products Collection [81].

#### Model #4: β-catenin inhibition for comercial compounds

Finally, the last QSAR model developed was formed by a *training set* composed by 1047 commercial compounds (93 actives and 954 inactive). External validation of the model was made on a *test set* of 207 compounds consisting of 17 β-catenin inhibitors and 190 inactive compounds.

As in the previous cases, the probability of activity within the inactive set is negligible due to its size.

All compounds were obtained from β-catenin Selleckchem database [143], literature [160–201] and the database MicroSource US Drugs Collection [98].

### Molecular descriptors

Topological descriptors codify information about molecular structure in a purely numerical way. This numerical format significantly eases the search of other molecules showing similar properties, thereby making easier the discovery of new drugs.

The 2D structure of each compound was drawn using the ChemDraw Ultra package (version 10.0) [202]. Every compound was characterized by a set of descriptors including constitutional and topological descriptors. Among the last stand the edge adjacency indices, walk and path counts, connectivity indices and topological charge indices [203]. Other graph-theoretical descriptors were also calculated but are not depicted here because their lack of effectiveness. All indices were calculated with Dragon software version 5.4 [43] and their values for every compound included in this study (*training*, *test*, and *virtual screening set*) are shown in the Supplementary Material.

Other complementary models (not shown here) were also applied to find molecules showing drug-like profiles. Among the properties considered in these models stand water solubility, toxicity, oral absorption, etc… (details on these additional models can be disclosed under request). Dragon software version 5.4 and in-house software were used to obtain the models.

### Modeling techniques

Linear discriminant analysis (LDA) was used to distinguish between the active and inactive compounds. It is a statistical method to find the best linear combination of variables (TIs in our case) that better distinguish between two or more categories or objects (in our case active or inactive as Akt or β-catenin inhibition). We start from a *training set* of compounds in which everyone is classified either as active or inactive. LDA was then applied to these two groups to get the discriminant function (DF) that better separate the two categories. The software used was Statistica 9.0 [204].

TIs were used as the independent variables and the two groups were balanced so that both (active and inactive) show the same weight, regardless of the number of compounds in each one.

The discriminant capability was assessed as the percentage of correct classifications in each set of compounds. The classification criterion was based on the minimum Mahalanobis distance (distance of each case to the mean of all the cases in a category) [205] and the quality of the discrimination was evaluated using the Wilks’ parameter, *λ* [206], which was obtained by multivariate analysis of variance that tests the equality of group means for the variable in the discriminant model. The shorter the Wilks’ parameter value, the smaller the overlap of the active and inactive (*λ* = 0 would mean a perfect separation between the groups).

The descriptors’ selection was carried out according to the value of the Fisher–Snedecor parameter (*F*) [207], which establishes the relevance of candidate variables. The descriptors input to compute the linear classification function are chosen in a stepwise-manner: at each step, the variable making the largest contribution to the division of the groups is introduced into the discriminant equation (or the variable that makes the smallest contribution is removed).

The validation of the selected function was done using an external *test set*. Approximately 20% of the data set was randomly selected as *test set* and therefore they were not used to create the model.

### Pharmacological activity distribution diagrams

A pharmacological distribution diagram (PDD) is a graphical representation that provides a direct way of visualizing the zones of minimum overlap between active and inactive compounds, as well as the region in which the probability of finding active compounds is maximum [208].

From a different perspective, a PDD is a frequency distribution diagram of dependent variables in which the ordinate represents the expectancy (probability of activity) and the abscissa the DF values in the range. For an arbitrary range of values of a given function, the expectancy of activity can be defined as *Ea* = *a/(i* +1*)*, where *a* is the number of active compounds in the range divided by the total number of active compounds and *i* is the number of inactive compounds in the interval divided by the total number of inactive compounds. The expectancy of inactivity is defined likewise as *Ei* = *i/(a*+1*)*. By means of these diagrams, it is easy to visualize the intervals in which there is a maximum probability to find new active compounds as well as the minimum probability to find inactive compounds.

### Topological virtual screening

Once the models are set up, it is possible to carry out a virtual screening in databases to select novel Akt and β-catenin inhibitors. Two commercial SPECS databases were screened, the first was made of some one thousand natural compounds and the second containing about 200 000 drug-like small molecules [209].

This screening led to the identification of 6 compounds showing potential chemotherapeutic activity as Akt and β-catenin inhibitors. Later on, the selected molecules underwent *in vitro* tests to confirm the expected activity.

### In Vitro Assays

#### Reagents and antibodies

Chemicals compounds with CAS n° 256378-54-8 (Inhibitor n°1), 663203-38-1 (Inhibitor n°2), 247079-73-8 (Inhibitor n°3), 689769-86-6 (Inhibitor n°4), 15940-61-1 (Inhibitor n°5) and 431925–096 (Inhibitor n°6) were obtained from Specs (Zoetermeeer, The Netherlands) and dissolved in DMSO. LY294002 and AT7519 were supplied by Deltaclon (Madrid, Spain) a Selleckchem supplier. Akt inhibitor IV and FH535 were obtained from Sigma-Aldrich (Madrid, Spain). Recombinant Human Wnt-3a (R&D Systems, Minneapolis, USA) was reconstituted at 200 μg/mL in sterile PBS containing 0.1% bovine serum albumin. The polyclonal antibodies against phospho-mTOR, mTOR, phospho-Akt-ser473, Akt and anti-rabbit IgG were from Cell Signaling Technology (Danvers, MA, USA) and anti-β-catenin was obtained from Santa Cruz Biotechnology (Bergheimer, Heidelberg, Germany). Monoclonal antibody against cyclin D1 was from EMD Millipore (Darmstadt, Germany) and anti-β-tubulin and anti-mouse IgG were from Sigma (Madrid, Spain). Anti-rabbit IgG conjugated to Alexa488 was from Invitrogen (Life Technologies, Alcobendas, Spain).

#### Cell cultures

PC3 cell line derived fron human prostate adenocarcinoma was obtained from ATCC (CRL-1435, Rockville, MD, USA) and maintained in RPMI medium containing 10% foetal bovine serum and 1% penicillin/streptomycin. Cells were used between passages 10 and 20 and seeded at a density of 20,000 cells/cm^2^. Sixteen hours post-seeding, medium was changed to serum free medium and treatments were performed 24 hours later. HT-29 cell line derived from a human colon adenocarcinoma (ATCC, Rockville, MD), was used between passages 15 and 25 and cultured in DMEM glucose concentration 4.5 g/L supplemented with 20% fetal bovine serum, penicillin (100 U/mL) and streptomycin sulfate (100 μg/mL) in a humidified 5% CO_2_ atmosphere at 37 °C. According to the solubility, the compounds assayed were dissolved in a maximum DMSO concentration of 0.5%.

#### Cytotoxicity assay

The effect of compounds on cell viability was evaluated with the 3-[4,5-dimethylthiazol-2-yl]-2,5-diphenyl-tetrazolium bromide (MTT) assay [210]. After 24 h seeding of HT-29 cells, the medium was replaced with fresh DMEM + 0.5% FBS. Two hours later, cells were exposed to compounds at the concentrations (1, 10, or 100 μM) in a 96-well microplate at 37 °C for 24 h. Same protocol was applied to PC-3 cells but cells were seeded in 12-well plates and mantained 24 hours in a serum-free medium prior the treatments (20, 50, 100, 150 and 200 μM).

The medium was then removed and 100 μL per well of a 0.5 mg/mL solution of MTT was added. The resulting solution was incubated at 37 °C until blue deposits were visible and then, this colored metabolite was dissolved in dimethyl sulfoxide (DMSO). Absorbance was measured at 490 nm with a Labsystems Multiskan EX plate reader (Helsinki, Finland). The results were expressed in absolute absorbance readings; a decrease in absorbance indicated a reduction in cell viability. The percentage of cell viability was determined as follows:

$$Cell\;viability\;(\%)=\frac{Optical\;density\;of\;treated\;cells}{Optical\;density\;of\;non\;–\;treated\;cells}\times 100$$

The experiments were performed a minimum of three times with three replicates per concentration.

#### Western blot

After 1, 4 or 48 hours of treatments according to the experiments, cells were lysed in ice-cold lysis buffer (50 mM Tris pH 7.4, 0.8 M NaCl, 5 mM MgCl2, 0.1% Triton X-100, 1 mM PMSF, 10 μg/mL soybean trypsin inhibitor, 1 μg/mL aprotinin, and 5 μg/mL leupeptin), and cleared by microcentrifugation. Protein concentration was determined by the Bradford method and equivalent protein amounts of each sample were loaded onto SDS-PAGE gels and transferred to PVDF membrane. Immunoblot analysis was performed followed by enhanced chemoluminescence detection as previously described [211–212].

#### Confocal microscopy

Cells seeded onto 1.5 mm glass coverslips were treated for 4 hours with 50 μM of the some of the selected potential chemotherapy agents: Inhibitors n°1-6. Then cells were fixed in 4% paraformaldehyde, permeabilized with 0.5% Triton X-100 and incubated with β-catenin antibody overnight at 4˚C. The cells were washed and incubated first with secondary antibody conjugated to Alexa488 and then with 4′-6-Diamidino-2-phenylindole (DAPI) as a nuclear counterstain. Cells were visualized in a Leica TCS SP5 laser-scanning confocal microscope with LAS-AF imaging software, using a 63X oil objective.

#### Statistical analysis

Statistical analysis was performed with a one-way analysis of variance (ANOVA) and Dunnett’s t test. The results are presented as the mean ± SEM. GraphPad Prism 4.0 software (GraphPad Software Inc., San Diego, CA, USA) was used for all calculations. Data related with PC3 cell lines are presented as the mean ± S.D. of the number of experiments indicated.

## Results and Discussion

### Topological Models

#### Model #1: Akt inhibition for natural compounds

The discriminant function (DF_1_) includes three variables, as shown below:

$${\text{DF}}_{1}=-2.046970-1.007164*\text{nR}09-0.000005*\text{Wap}+2.276221*\text{EEig}11\text{r}$$

The parameters accounting for the significance of this equation were:

N=153,F=28.628,λ=0.6344,p<0.00002

where N is the number of data compounds; F, Fisher–Snedecor parameter; λ, Wilks’ lambda; p, statistical significance; nR09, number of 9-membered rings; Wap, all path Wiener index; EEig11r, eigenvalue 11 from edge adj. matrix weighted by resonance integrals.

**Fig 3:**
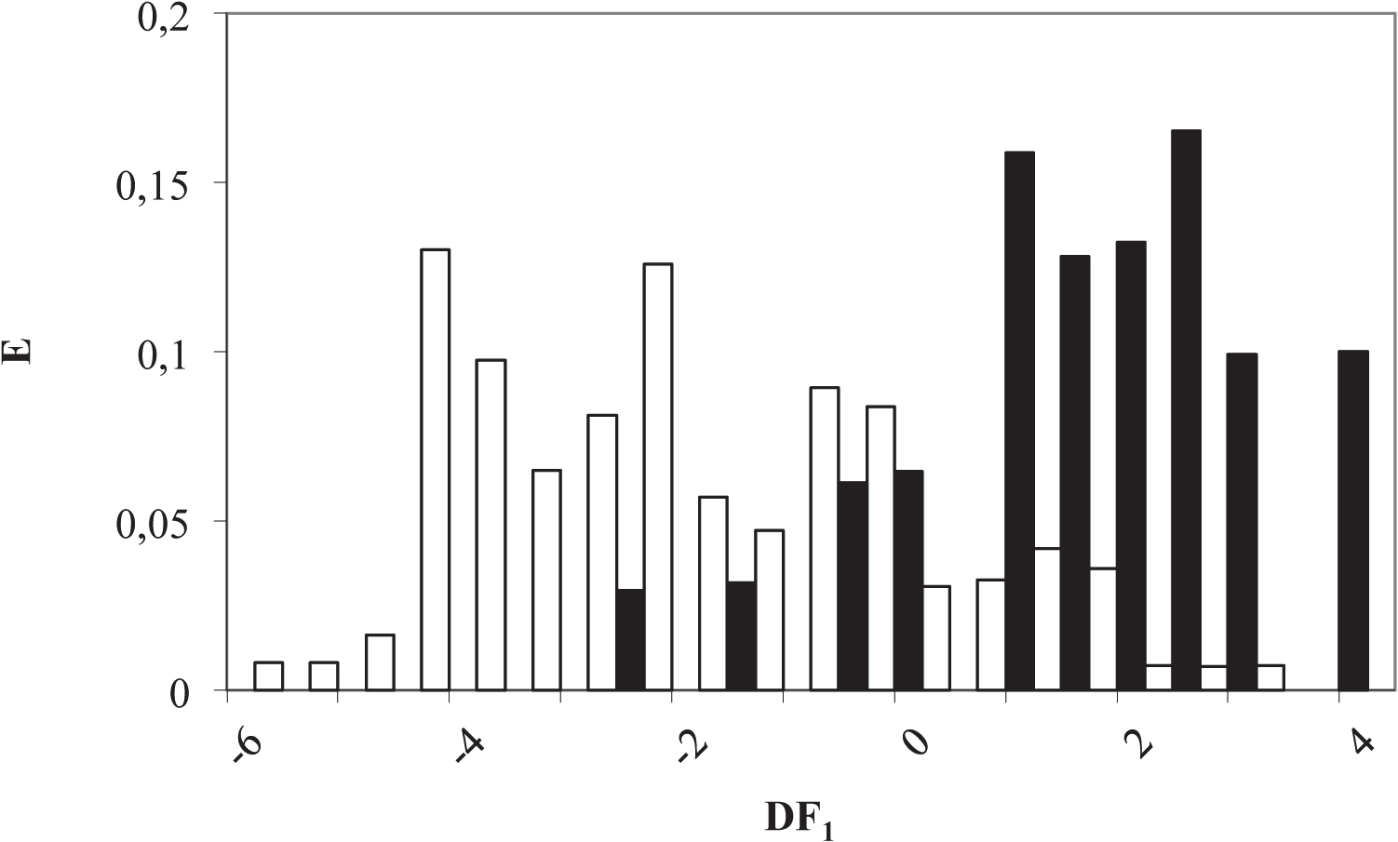
Pharmacological distribution diagram for natural Akt inhibitors obtained using the DF_1_ (the black colour represents Akt inhibitors and the white colour, the compounds without Akt inhibition activity).

According to DF_1_, a compound is classified as Akt natural inhibitor if DF_1_ takes values from 0 to 4; on the other hand, a compound will be labelled as inactive when DF_1_ takes values between -0.5 and -6 (see Fig 3). Finally, molecules scoring DF_1_ > 4 or DF_1_ < -6 will be non-classified by this model. By applying this criterion to the *training set* composed by 153 compounds, 26 out of 30 natural Akt inhibitors were correctly classified (81% accuracy) while 101 out of 123 inactive compounds were also matched (82% accuracy), as can be seen in Table 1.

**Table 1:** Classification matrix obtained through the selected DF_1_ for *training* and *test set*.

	PERCENT-CORRECT CLASSIFICATION (%)	COMPOUNDS		
		Classified as active	Classified as inactive	Non-classified
***TRAINING SET***				
Active group	81	26	4	_
Inactive group	82	22	101	_
***TEST SET***				
Active group	63	5	3	_
Inactive group	87	3	27	1

The best way to evaluate the quality of any DF is to apply it to an external validation group (*test set*). In our case, this group was made up of 39 compounds (8 active and 31 inactive as Akt inhibitors) which were not included in the *training set*. *Test set* was randomly selected with a percentage of 20 of all *data set*. The model enabled a correct classification of 63% of active group (5 out of 8 compounds) and 87% of the inactive set (27 out of 31 compounds), as outlined in Table 1.

Furthermore, although the strict DF application leads to the loss of some of the active compounds (37%), the important point is that 87% of the inactive compounds were correctly classified, so that the risk of including “false active” is minimized, which means that our model has a high specificity in discovering new Akt natural inhibitors.

#### Model #2: Akt inhibition for comercial compounds

A new discriminant function (DF_2_) was used for Akt inhibition by commercial compounds. This function includes five variables as follows:

$$DF_{2}=-13.835+0.073*T\left(O\ldots Br\right)-0.002*SRW08+0.137*MPC04+6.256*piPC02-1.712*piPC05$$

Statistical data and parameters accounting for the significance of this equation were:

N=647,F=5.950,λ=0.956,p<0.00002

where *N* is the number of data compounds; *F*, Fisher–Snedecor parameter; *λ*, Wilks’ lambda; *p*, statistical significance; T(O…Br),topological distance between oxygen and bromide; SRW08, self-returning walk count of order 08; MPC04, molecular path count of order 04; piPC02, molecular multiple path count of order 02; piPC05, molecular multiple path count of order 05.

**Fig 4:**
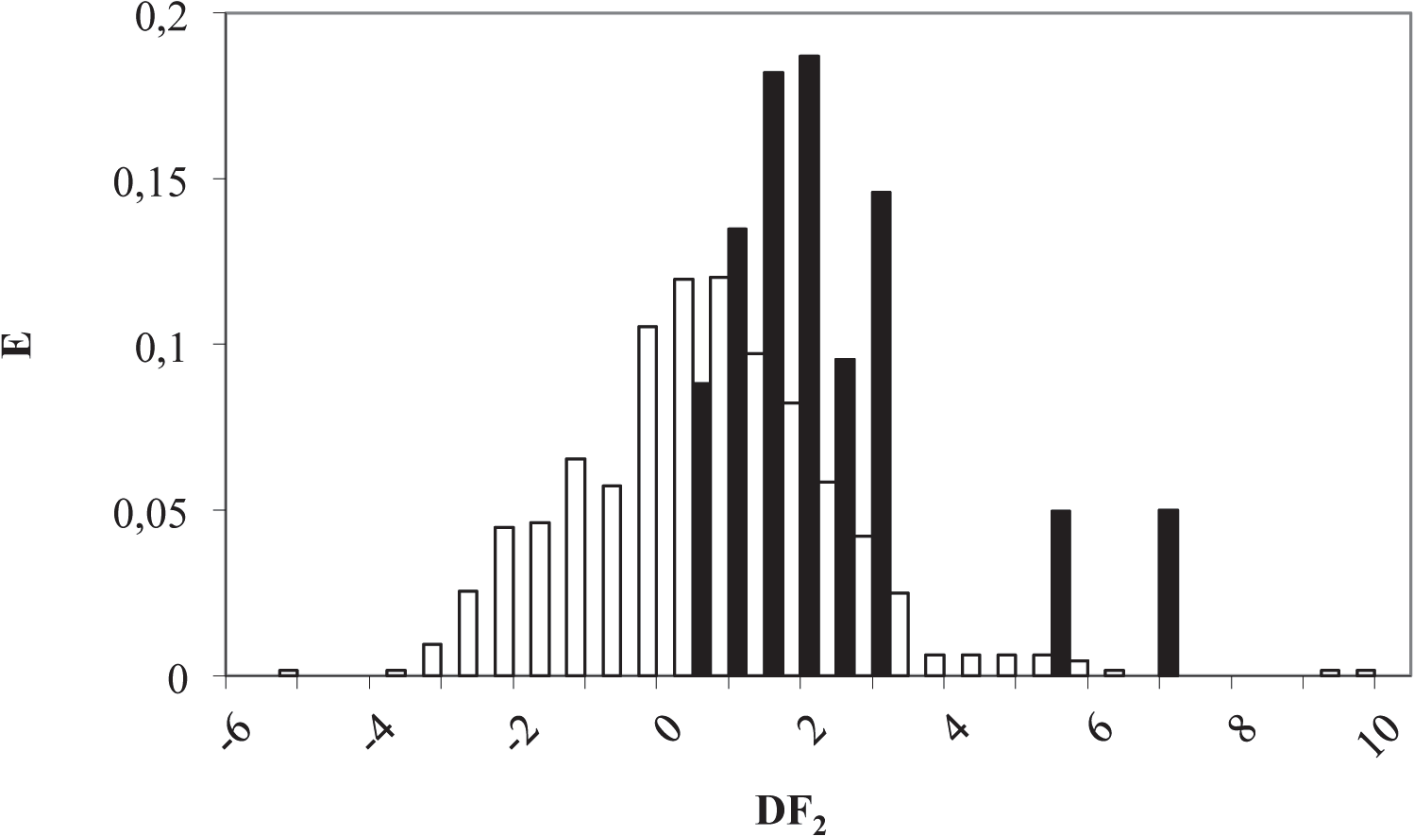
Pharmacological distribution diagram for Akt inhibitors obtained using the DF_2_ (the *black colour* represents Akt inhibitors and the *white colour*, the compounds without Akt inhibition activity).

According to DF_2_, a compound is classified as Akt inhibitor if its DF values range from 1.5 to 7.5. On the other hand, a compound will be labeled as inactive if DF_2_ stands between 1.5 and -6 (see Fig 4). Finally, molecules scoring DF_2_> 7.5 or DF_2_ < -6 are considered as non-classified by this model. By applying the model criterion to the *training set* composed by 647 compounds, 16 out of 20 (80%) were correctly classified as Akt inhibitors, while 452 out of 627 inactive compounds were also well classified (72% accuracy), as can be seen in Table 2.

**Table 2:** Classification matrix obtained through the selected DF_2_ for *training* and *test set*.

	PERCENT-CORRECT CLASSIFICATION (%)	COMPOUNDS		
		Classified as active	Classified as inactive	Non-classified
***TRAINING SET***				
Active group	80	16	4	_
Inactive group	72	173	452	2
***TEST SET***				
Active group	83	5	1	_
Inactive group	81	38	161	1

An external validation of DF_2_ was performed over 206 compounds (6 active and 200 inactive molecules as Akt inhibitors) not included in the *training set*. Twenty-percent of all data were used for t*est set*. DF_2_ was able to classify correctly 83% of the active group (5 out of 6 compounds) and 81% of inactive (161 out of 200 compounds) as illustrated in Table 2.

Therefore, application of DF leads to discard just 17% of the active compounds and 19% of inactive compounds. In other words, this model has a high specificity and sensitivity.

#### Model #3: β-catenin inhibition for natural compounds

To select β-catenin natural inhibitors another discriminant function was developed (DF_3_). This function includes five variables, as shown below:

$$DF_{3}=-12.179-0.427*Dz+1.174*S2K+6.219*PCR+1.634*X2sol+79.748*JGI4$$

Statistical data and parameters accounting for the significance of this equation were as follows:

N=111,F=7.656,λ=0.733,p<0.00002

where *N* is the number of data compounds; *F*, Fisher–Snedecor parameter; *λ*, Wilks’ lambda; *p*, statistical significance; Dz, Pogliani index; S2K, 2-path Kier alpha-modified shape index; PCR, ratio of multiple path count over path count; X2sol, solvation connectivity index chi-2; JGI4, mean topological charge index of order 4.

**Fig 5:**
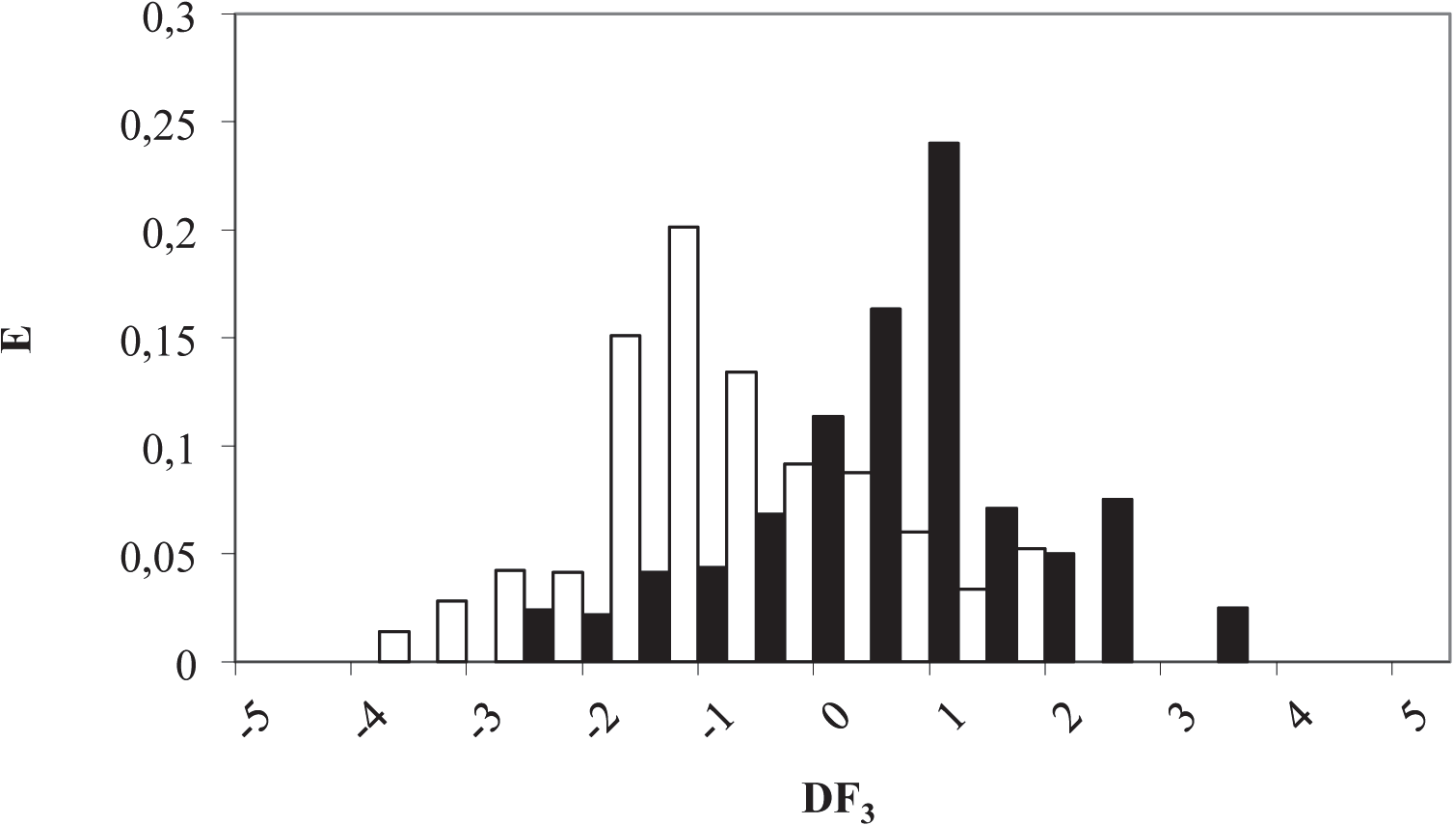
Pharmacological distribution diagram for natural β-catenin inhibitors obtained using the DF_3_ (the *black color* represents natural β-catenin inhibitors and the *white color*, the compounds without β-catenin inhibition activity).

According to DF_3_, a compound is classified as β-catenin natural inhibitor if it takes values between 0 and 5. On the other hand, a compound will be labeled as inactive when DF_3_ adopt values from 0 to -5. Finally, molecules scoring DF_3_ > 5 or DF_3_< -5 will be non-classified by this model (see Fig 5). Applying the above criteria to a *training set* composed by 111 compounds, 31 out of 40 natural β-catenin inhibitors were correctly classified for the model (78% correctly classified) while 52 out of 71 inactive compounds were also well classified (73% accuracy), as can be seen in Table 3.

**Table 3:** Classification matrix obtained through the selected DF_3_ for *training* and *test set*.

	PERCENT-CORRECT CLASSIFICATION (%)	COMPOUNDS		
		Classified as active	Classified as inactive	Non-classified
***TRAINING SET***				
**Active group**	78	31	9	_
**Inactive group**	73	19	52	_
***TEST SET***				
**Active group**	67	16	6	2
**Inactive group**	88	2	15	_

The validation of the DF was performed on an external group (*test set*). The group was made up of 41 compounds (24 active and 17 inactive as β-catenin inhibitors) not previously included in the *training set*. The *test set* was randomly selected as 20% of all *data set*. In this case, the model was able to correctly classify 67% of active group (16 out of 24 compounds) and 88% of the inactive one (15 out of 17 compounds) as can be seen in Table 3.

As in previous cases, the strict DF application results in the loss of 33% of the active compounds but 88% of the inactive natural compounds were correctly classified, thereby minimizing the risk of including “false active” compounds. That confirms our model’s high specificity to predict β-catenin natural inhibitors.

#### Model #4: β-catenin inhibition by comercial compounds

A discriminant function modeling β-catenin inhibition by commercial molecules was finally calculated (DF_4_). This equation includes four variables, as shown below:

$$DF_{4}=-0.381+0.254*SCBO-0.292*nN-0.075*ZM1+1.159*GGI4$$

Statistical data and parameters accounting for the significance of this equation were as follows:

N=837,F=15.498,λ=0.931,p<0.00002

where *N* is the number of data compounds; F, Fisher–Snedecor parameter; λ, Wilks’ lambda; p, statistical significance; SCBO, sum of conventional bond orders (H-depleted); nN, number of Nitrogen atoms; ZM1, First Zagreb index M1; GGI4, topological charge index of order 4.

**Fig 6:**
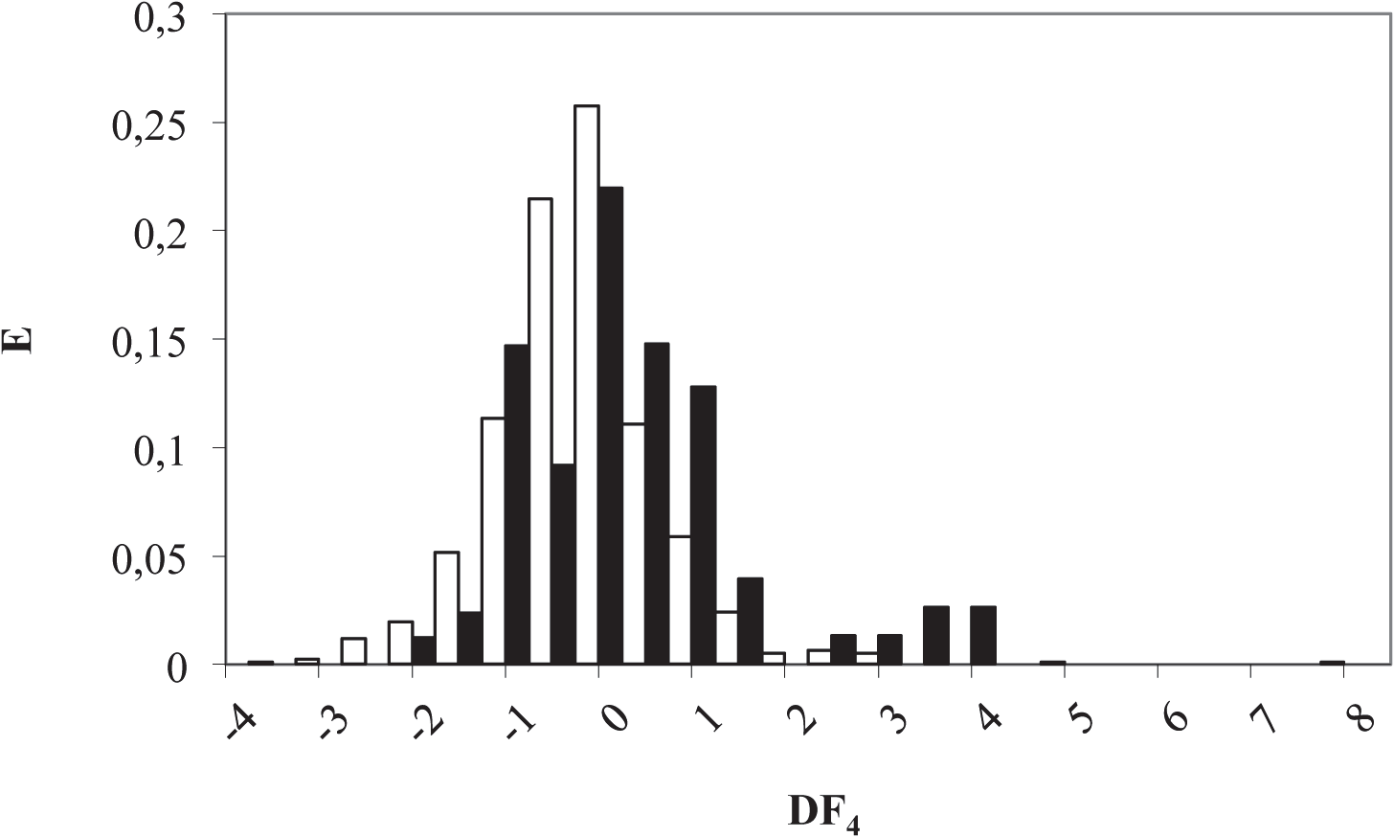
Pharmacological distribution diagram for β-catenin inhibitors obtained using the DF_4_ (the *black color* represents β-catenin inhibitors and the *white color*, the compounds without β-catenin inhibition activity).

A compound is classified as β-catenin inhibitor if DF_4_ takes values from 0 to 4.5, whereas a compound will be labeled as inactive if DF_4_ adopt values between 0 and -4. Finally, molecules scoring DF_4_ > 4.5 or DF_4_ < -4 will be non-classified (see Fig 6). For the entire set of 840 compounds, 48 out of 73 β-catenin inhibitors (66%) were correctly classified by the model, whilst 570 out of 764 inactive compounds were also well classified (75% accuracy), as illustrated in Table 4.

**Table 4:** Classification matrix obtained through the selected DF_4_ for *training* and *test set*.

	PERCENT-CORRECT CLASSIFICATION (%)	COMPOUNDS		
		Classified as active	Classified as inactive	Non-classified
***TRAINING SET***				
Active group	66	48	25	_
Inactive group	75	192	570	2
***TEST SET***				
Active group	88	14	2	_
Inactive group	74	49	141	_

In this case, the *external validation set* included 206 compounds (16 active and 190 inactive). *Test set* was a random selection of 20% of all *data set*. The model was able to correctly classify 88% of the active group (14 out of 16 compounds) and 74% of the inactive one (141 out of 190 compounds) as shown in Table 4.

According to this results, we can conclude that this model presents a higher sensitivity (88%) but a less satisfactory specificity since only 74% of the inactive compounds were recorded, missing the remaining 26%. This could lead to select “false active” compounds, but it can be avoided by applying the four models stepwise.

### Topological virtual screening

Based on the four discriminant models described (DF_1-4_), a *virtual screening* was carried out on two commercial chemical libraries from SPECS. Table 5 shows the compounds selected (naturals and synthetics). DF_1_ and DF_3_ were applied only to the natural compounds.

**Table 5:** Values of DF and probability of activity as Akt and β-catenin inhibitors of the selected anti-cancer agents after carried on a *virtual screening of* SPECS natural and screening compounds databases.

COMPOUNDS	CAS registry n°	Akt natural inh model		Akt inh. model		β-catenin natural inh. model		β-catenin inh. model	
		DF_1_	P. (Activ.)	DF_2_	P. (Activ.)	DF_3_	P. (Activ.)	DF_4_	P. (Activ.)
Inhibitor n°1*	256378-54-8	1.68	0.843	1.67	0.699	1.75	0.851	1.55	0.82
Inhibitor n°2	663203-38-1			3.97	0.67			1.31	0.781
Inhibitor n°3	247079-73-8			2.52	0.769			1.24	0.769
Inhibitor n°4	689769-86-6			3.02	0.921			0.36	0.582
Inhibitor n°5	15940-61-1			2.24	0.62			0.77	0.679
Inhibitor n°6	431925-09-6			4.28	0.914			0.19	0.539

DF: discriminant function; P.(Activ.): probability of being classified as active by the model.

*Natural compound.

### In vitro assays

#### Cytotoxicity assay: HT-29 and PC3 cell line

HT-29 cells were exposed to the selected Akt and β-catenin potential inhibitors selected by the DF_1-4_ models, in order to determine cell viability. As shown in Table 6, several among the selected compounds were better or similar to the reference β-catenin inhibitor (FH535), Akt inhibitor (LY294002) and multi-CDK inhibitor (AT7519). For instance, Inhibitor n°4 at 100 μM is able to kill 48% of HT-29 cells. This compared with FH535, LY294002 and AT7519 at 100 μM inhibition rates represent 38%, 51% and 36%, respectively. It’s quite a promising result in colorectal cancer cells inhibition.

**Table 6:** Effect of selected anti-cancer agents on the HT-29 cell viability.

Group	Concentration (μM)	A ± SEM	Cell viability (%)
**Blank**	**0**	0.88 ± 0.02	-
**Vehicule (DMSO)**	**1**	0.86 ± 0.01	97
	**10**	0.86 ± 0.07	98
	**100**	0.80 ± 0.10	91
**Inhibitor N°1**	**1**	0.84 ± 0.10	95
	**10**	0.77 ± 0.07	87
	**100**	0.86 ± 0.06	98
	**1**	0.87 ± 0.13	99
**Inhibitor N°2**	**10**	0.79 ± 0.11	89
	**100**	0.82 ± 0.11	93
**Inhibitor N°3**	**1**	0.84 ± 0.10	95
	**10**	0.78 ± 0.05	88
	**100**	0.63 ±0.08	72
**Inhibitor N°4**	**1**	0.77 ± 0.05	87
	**10**	0.75 ± 0.08	85
	**100**	0.46 ± 0.06**	52**
**Inhibitor N°5**	**1**	0.85 ± 0.12	96
	**10**	0.78 ± 0.09	89
	**100**	0.71 ± 0.07	80
**FH535**	**1**	0.80 ±0.02	91
	**10**	0.72 ± 0.06	81
	**100**	0.55 ± 0.08*	62*
**LY294002**	**1**	0.90 ± 0.03	102
	**10**	0.71 ± 0.04	81
	**100**	0.43 ± 0.05***	49***
**AT7519**	**1**	0.59 ± 0.05	67
	**10**	0.59 ± 0.02	67
	**100**	0.56 ± 0.01	64

Values were expressed in function of the blank (untreated cells) and represent mean absorbance ± SEM,

and are representative of at least three independent experiments per group. Differentiation with the blank

group (untreated cells) were determined by mean of a one-way analysis of variance (ANOVA) followed by

Dunnett's multiple comparisons test Dunnett’s t test.

No significant difference with the blank group (P>0.05);

*Significant difference with the blank group (P values from 0.01 to 0.05);

**Very significant (P values from 0.001 to 0.01);

***Extremely significant (P values from 0.0001 to 0.001) n = 3; Dunnett’s t test).

It was investigated whether the selected cancer chemotherapeutics agents had any effect on the proliferation of human prostate cancer cells. PC-3 cells were treated with different doses of the Inhibitors n°1-6 for 48 h and cell viability was monitored by MTT. Results show that Inhibitor n°4 totally blocked the viability of prostate cancer cells (Table 7). Inhibitors n°1 and n°6 also decreased cell viability although in a lesser extent; Inhibitor n°3 exerted a slight decrease whereas compounds Inhibitor n°2 and n°5 didn’t have any effect (Table 7).

**Table 7:** Effect of selected anti-cancer agents on the PC-3 cell viability.

Group	Concentration (μM)	A ± SEM	Cell viability (%)
**Blank**	**0**	0.4 ± 0.02	
**Vehicule (DMSO)**	**10**	0.4 ± 0.03	100
**Inhibitor N°1**	**50**	0.35 ± 0.04	88
	**100**	0.24± 0.03	61**
	**200**	0.16 ± 0.03	42***
**Inhibitor N°2**	**50**	0.37 ± 0.02	101
	**100**	0.37± 0.02	101
	**200**	0.34± 0.02	92
**Inhibitor N°3**	**50**	0.32± 0.02	83**
	**100**	0.32± 0.03	83*
	**200**	0.28± 0.02	72**
**Inhibitor N°4**	**50**	0.22± 0.01	60***
	**100**	0.16± 0.01	44**
	**200**	0.01± 0.001	3***
**Inhibitor N°5**	**50**	0.34± 0.001	101
	**100**	0.36± 0.001	106
	**200**	0.36± 0.005	107
**Inhibitor N°6**	**50**	0.34± 0.06	88
	**100**	0.32± 0.05	83*
	**200**	0.27± 0.06	69*

Values were expressed in function of the vehicle-treated cells and represent mean absorbance ± SD, and are representative of at least two experiments performed in duplicate. Differentiation with the blank group (untreated cells) were determined by mean of a Student ‘s T test. No significant difference with the blank group (P>0.05)

*Significant difference with the blank group (P values from 0.01 to 0.05)

**Very significant (P values from 0.001 to 0.01)

***Extremely significant (P values from 0.0001 to 0.001) n = 4)

#### Akt inhibition

**Fig 7:**
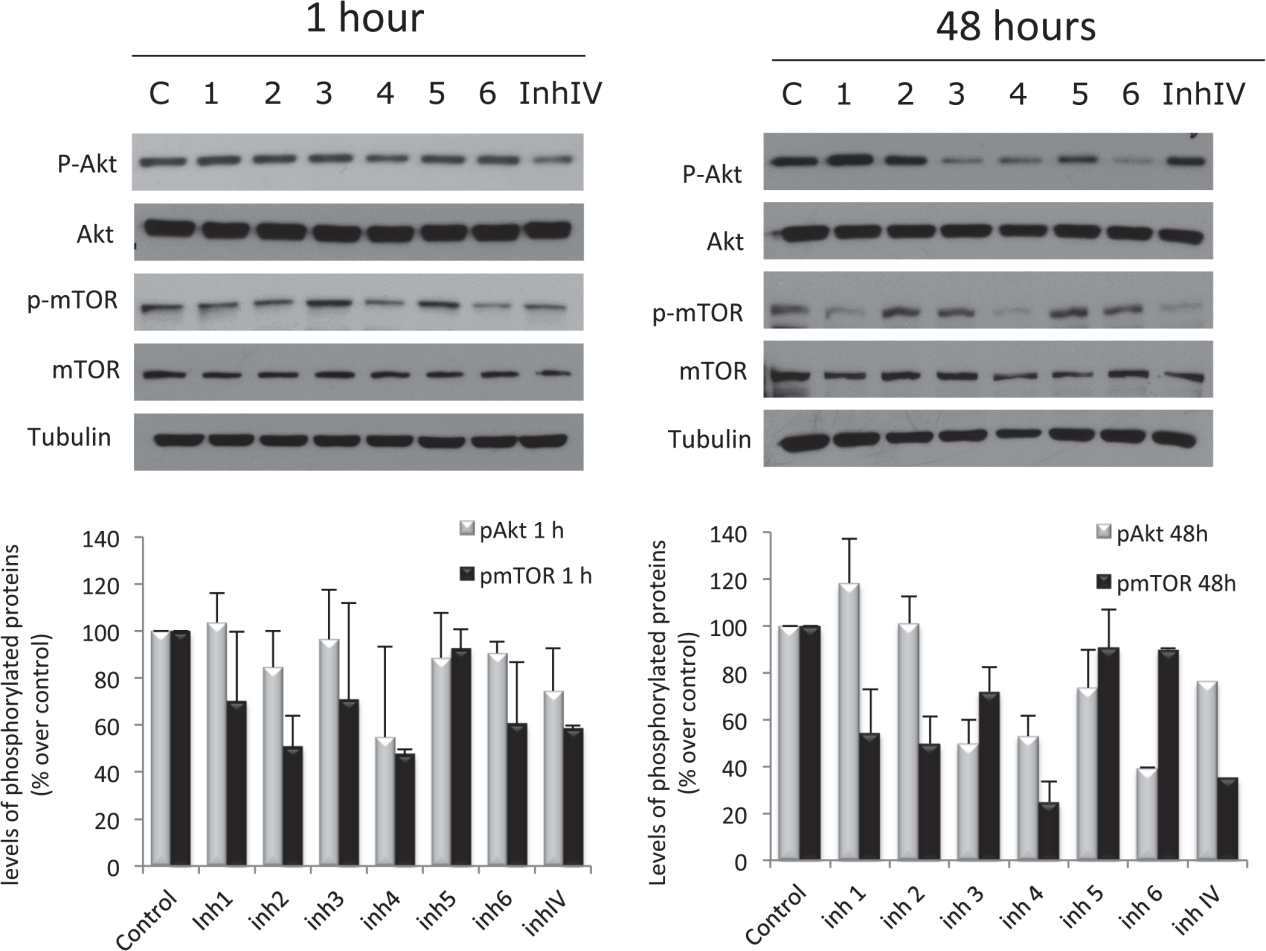
Effect of of selected anti-cancer agents on Akt/mTOR signaling pathway. PC-3 cells were treated with vehicle (C) or 50 μM of the selected compounds and proteins were detected by Western blot. Upper panel, a representative image of three different experiments. Lower panel, densitometric values represented as the mean ± S.D. of the three experiments.

Akt activation involves the phosphorylation of two residues: threonine 308 (Thr308) in the activation loop and serine 473 (Ser473) in the C-terminal hydrophobic motif. The phosphorylation of both of these regulatory sites leads to complete activation of the enzyme. To evaluate the inhibitory ability of the selected compounds against Akt we measured the phosphorylation of the enzyme in serine 347. As shown in Fig 7, Inhibitor n°4 at 1 hour of treatment, induced a decrease in Akt phosphorylation at Ser473. This effect may be compared with the well-known Akt inhibitor IV, a cell-permeable and reversible benzimidazole compound that inhibits Akt phosphorylation/activation. When cells were incubated with the compounds for 48 hours, this effect was even greater and was also observed for Inhibitors n°3 and n°6 (Fig 7).

To assess the impact of the compounds on downstream molecules of the Akt signaling pathway, we used Western blot analysis to observe phosphorylation status and total protein expression of the mammalian target of rapamycin (mTOR), a key effector downstream of Akt. As observed in Fig 7, all the agents tested except Inhibitor n°5, decreased mTOR phosphorylation, being the Inhibitors n°2, n°4 and n°6 the most effective at 1 hour and Inhibitors n°1, n°2 and n°4 at 48 hours.

These results indicate that all the compounds selected, except Inhibitor n°5, were able to inhibit Akt signaling pathway although with different efficacy.

#### β-Catenin inhibition

To investigate whether the compounds also inhibited β-catenin pathway we measured β-catenin levels as well as its downstream well-characterized target gene cyclin D1.

**Fig 8:**
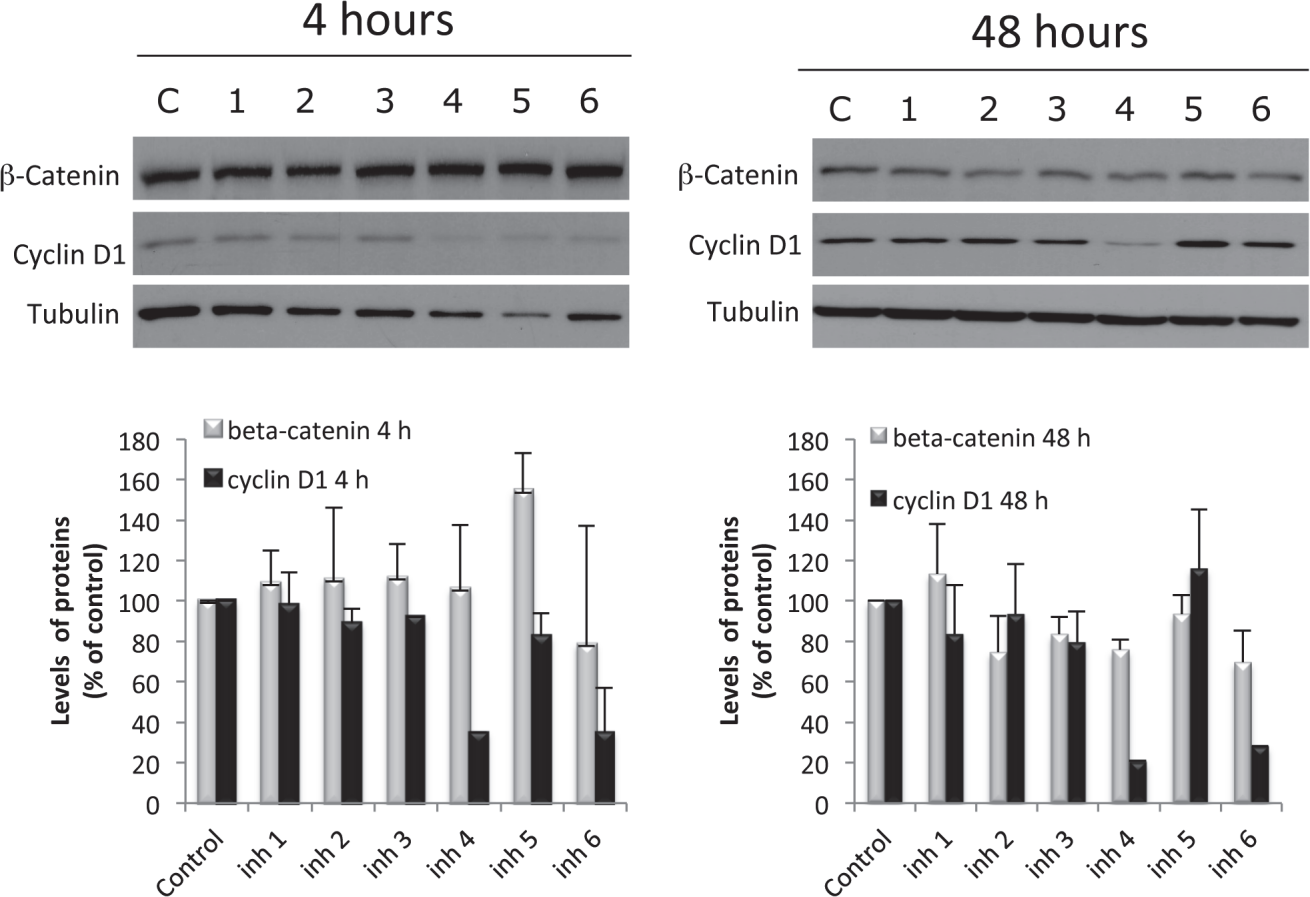
Effect of selected anti-cancer agents on β-Catenin/CyclinD1 signaling pathway. PC-3 cells were treated with vehicle (C) or 50 μM of the selected compounds and proteins were detected by Western blot. Upper panel, a representative image of three different experiments. Lower panel, densitometric values represented as the mean ± S.D. of the three experiments.

In the basal situation, much of the cellular β-catenin is bound to E-cadherin on the cell membrane. Cytosolic β-catenin maintained in an inactive state through its interaction with a large protein complex with several proteins including the GSK-3β. In this situation β-catenin is phosphorylated mainly by GSK-3β and labelled for polyubiquitination and degradation into proteasome. Under these conditions, β-catenin targeted genes are repressed. When the pathway is activated, GSK-3β is inhibited leading to the stabilization and accumulation of cytoplasmic β-catenin, which then enters the nucleus, and induces the expression of its target genes. One of the better characterized β-catenin-regulated gene is Cyclin D1 which promote cell cycle progression. To test the effect of selected compounds on the β-catenin pathway levels of β-catenin and Cyclin D1 were determined by Western blot. Fig 8 shows that the Inhibitors n°4 and n°6 were the most efficacious in inhibiting the expression of Cyclin D1, although Inhibitors n°1, n°2 and n°3 also exhibited a weak repression.

**Fig 9:**
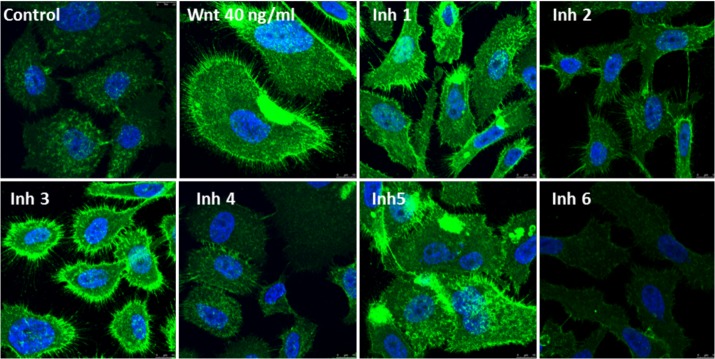
Effect of selected anti-cancer agents on β-catenin cellular distribution in PC-3 cells. PC-3 cells were seeded on glass coverslips and pre-treated for 30 min with 50 μM of the inhibitors and then co-treated with 40 ng/ml Wnt3 (Wnt) for 4 h. Cells were stained with polyclonal antibody anti-beta-catenin followed by Alexa-Fluor488-conjugated anti rabbit IgG as described in methods. Confocal image shown is representative of two experiments.

To further investigate the inhibitor role of the selected compounds, PC-3 cells were stimulated with the well-stablished β-catenin/Cyclin D1 pathway activator Wnt3a (Wnt). When cells were stimulated with Wnt, β-catenin was recruited by membrane adherens junctions and accumulated in the nucleus (Fig 9). When cells were pre-treated with the selected compounds β-catenin redistribution was inhibited. This effect was well apreciated in Inhibitors n°2, n°4 and n°6 (Fig 9). Moreover, levels of β-catenin in Inhibitor n°6-treated cells almost desappeared (Fig 9).

As seen in the Figs 8 and 9, the selected cancer chemotherapeutic agents were able to impact both the subcellular redistribution and the activity of β-catenin as they were able to inhibit its nuclear-cytoplasmic shuttling and its recruitment at plasma membrane as well as the expression of its target gene CyclinD1. The Inhibitors n°2, n°4 and n°6 were the most effective.

Notably, the compounds that inhibited both Akt and β-catenin had the greatest impact on cell viability supporting the idea that dual inhibitor of the Akt/mTOR pathway and β-catenin may result in a potent anti-proliferative effect against human prostate cancer PC-3 cells, although the putative impact of the compounds in other signaling pathways is not ruled out.

**Fig 10:**
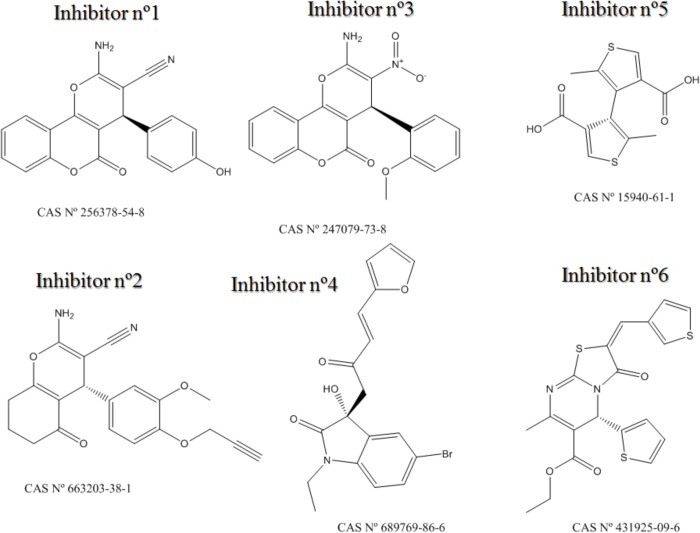
Cancer chemotherapeutic agents selected by Molecular Topology.

Therefore, after applying several models developed by Molecular Topology approach, six compounds has been selected as potential cancer chemotherapeutic agents. After *in vitro* study whether they inhibit Akt, mTOR, β-catenin and/or Cyclin D1 at different times of incubation; we can affirm that five out of these six compounds (see Fig 10) inhibit at least one of the studied targets. We can also state that these five compounds affect PC3 cell viability with different potency. And, we can remark also that Inhibitor n°4 also reduce cell viability on HT-29 cell line.

Finally, we can confirm that this five selected compounds could be used as cancer agents interacting with Akt/mTOR and Wnt/β-catenin pathways in colorectal and prostate human cancer cell lines.

Although it is evident that complex properties, such as inhibition of Akt or β-catenin, cannot be discussed in such simple “structural” terms, thanks to the QSAR study results, some interesting consequences can be pointed out.

Thus, in equations DF_1_ to DF_4_, there are indices more or less explicitly related to the existence of high conjugation (as for instance EEig11r, piPC02 or SCBO). Other descriptors, such as nR09, evaluate the presence of condensed rings. It is noteworthy that most selected compounds show condensed ring with large conjugation due to aromatic rings such as benzene, furan or thiophene. In this regard, it is significant that the only molecule not showing condensed rings (Inhibitor n°5) is inactive despite having two highly aromatic thiophene rings.

Furthermore, the two molecules having six-five member condensed rings are by far the most potent ones. Indeed Inhibitor n°4 exhibits the dihydro-indolone moiety and Inhibitor n°6 the thiazolopirimidinone ring. The next in the ranking of potency is Inhibitor n°2, which has two condensed six-member rings. Interestingly, the two less active compounds (Inhibitors n°1 and n°3) contain three condensed rings, namely the amino-pyrano-chromenone moiety, what points toward a maximum of two condensed rings for optimal activity.

It is also interesting to note that all selected molecules include one chiral carbon, which suggests that the activity of the enantiomers might not be as significant as predicted by the topological models, since these models do not take into account the chirality.

## Conclusions

Molecular Topology has been used to select novel potential anti-cancer compounds with prevention or therapeutic activity. The *in vitro* evaluation of the selected compounds, CAS n° 256378-54-8 (Inhibitor n°1), 663203-38-1 (Inhibitor n°2), 247079-73-8 (Inhibitor n°3), 689769-86-6 (Inhibitor n°4), 15940-61-1 (Inhibitor n°5) and 431925–096 (Inhibitor n°6), demonstrated significant activity as Akt and β-catenin inhibitors for all compounds except for Inhibitor n°5. However, particularly Inhibitors n°4 and n°6, can be considered as novel chemotherapy *hits* by dually inhibit Akt and β-catenin. Moreover, they also have demonstrated activity over prostate cancer cell line (Inhibitors n°4 and n°6) and on a human colorectal cancer cell line (Inhibitor n°4).

Topological Virtual Screening upon four discriminant models (DF_1-4_) resulted in an overall accuracy of five out of six compounds which showed the predicted activity. Accordingly, it can be concluded that Molecular Topology is a reliable and useful tool in the search and discovery of novel chemotherapeutic agents acting on Akt and beta-catenin pathways. The presented exhaustive performance opens new horizons for MT as a time-cost effective resource. Finally, it must be point out that the next step could be perform rational drug design based on the structures and then, carry on *in vivo* test to confirm their effectiveness as potential cancer chemotherapeutic agents.

## Supporting Information

S1 Table Compounds used in the training set and corresponding values of the DF_1_ to Akt natural inhibitors. (DOCX)

S2 Table Compounds used in the test set and corresponding values of the DF_1_ to Akt natural inhibitors. (DOCX)

S3 Table Compounds used in the training set and corresponding values of the DF_2_ to Akt inhibitors. (DOCX)

S4 Table Compounds used in the test set and corresponding values of the DF_2_ to Akt inhibitors. (DOCX)

S5 Table Compounds used in the training set and corresponding values of the DF_3_ to β-catenin natural inhibitors. (DOCX)

S6 Table Compounds used in the test set and corresponding values of the DF_3_ to β-catenin natural inhibitors. (DOCX)

S7 Table Compounds used in the training set and corresponding values of the DF_4_ to β-catenin inhibitors. (DOCX)

S8 Table Compounds used in the test set and corresponding values of the DF_4_ to β-catenin inhibitors. (DOCX)

S9 Table Selected compounds as potential anti-cancer agents by the virtual screening of SPECS databases by applying DF_1_-_4_. (DOCX)
