# Supplementary material for: Novel Cancer Chemotherapy Hits by Molecular Topology: Dual Akt and Beta-Catenin Inhibitors
Source: PLoS One. 2015 Apr 24;10(4):e0124244. doi: 10.1371/journal.pone.0124244 (PMC4409212; doi:10.1371/journal.pone.0124244)
Supplement: S1 Table — (DOCX) [file pone.0124244.s001.docx]

**S1 Table. Compounds used in the *training set* and corresponding values of the DF_1_ to Akt natural inhibitors.**

| **COMPOUNDS** | **nR09** | **Wap** | **EEig11r** | **DF** | **CLASS** | **P. (Activ.)** |
| --- | --- | --- | --- | --- | --- | --- |
| **ACTIVE GROUP** | | | | | | |
| Adenosinemonophosphate [47] | 1 | 9225 | 0.811 | -1.252 | I | 0.222 |
| Berbamine [48] | 0 | 956363 | 2.969 | 0.168 | A | 0.540 |
| Calcidiol [49] | 1 | 17594 | 1.832 | 1.032 | A | 0.737 |
| Celastrol [50] | 0 | 93004 | 2.186 | 2.487 | A | 0.923 |
| Chlorogenic acid [51] | 0 | 5830 | 1.002 | 0.206 | A | 0.551 |
| Enoxolone [52] | 0 | 99722 | 2.231 | 2.558 | A | 0.928 |
| Gambogic acid amide [53] | 2 | 367988 | 3.156 | 1.375 | A | 0.798 |
| Geneticin [54] | 0 | 22326 | 1.786 | 1.912 | A | 0.871 |
| Ginkgolide B [55] | 1 | 94014 | 2.075 | 1.222 | A | 0.772 |
| Hederagenin [56] | 0 | 98135 | 2.179 | 2.447 | A | 0.920 |
| Hesperidin [57] | 0 | 67545 | 2.892 | 4.215 | A | 0.985 |
| Hyperforin [58] | 0 | 17839 | 2.138 | 2.735 | A | 0.939 |
| Isoginkgetin [59] | 0 | 192923 | 2.707 | 3.198 | A | 0.961 |
| Kojic acid [60] | 0 | 320 | 0 | -2.048 | I | 0.114 |
| Madecassic acid [61] | 0 | 111544 | 2.452 | 3.005 | A | 0.953 |
| Mangiferin [62] | 0 | 36882 | 1.788 | 1.848 | A | 0.864 |
| Naringin [63] | 0 | 97739 | 2.865 | 4.01 | A | 0.982 |
| Pomiferin [64] | 0 | 35525 | 2.084 | 2.528 | A | 0.926 |
| Ouabain [65] | 1 | 180066 | 2.865 | 2.612 | A | 0.932 |
| Palmatine [66] | 0 | 31467 | 1.487 | 1.188 | A | 0.766 |
| Peoniflorin [67] | 1 | 34902 | 2.075 | 1.503 | A | 0.818 |
| Piperine [68] | 1 | 6450 | 1.222 | -0.303 | I | 0.425 |
| Pristimerin [69] | 0 | 100629 | 2.187 | 2.453 | A | 0.921 |
| Quercetin [70] | 0 | 8350 | 0.88 | -0.084 | I | 0.479 |
| Rhapontin [71] | 0 | 17239 | 1.766 | 1.891 | A | 0.869 |
| Rutin [62] | 0 | 98059 | 2.876 | 4.034 | A | 0.983 |
| Silibinin [72] | 0 | 81287 | 2.461 | 3.169 | A | 0.960 |
| Sitosterol [73] | 1 | 40984 | 1.996 | 1.295 | A | 0.785 |
| Ursolic acid [74] | 0 | 90531 | 2.277 | 2.706 | A | 0.937 |
| Vitamin E [75] | 0 | 12536 | 1.827 | 2.052 | A | 0.886 |
| **INACTIVE GROUP** | | | | | | |
| 2,6-Dimethoxyquinone | 0 | 506 | -0.882 | -4.057 | I | 0.017 |
| 2-Acetylpyrrole | 0 | 159 | 0 | -2.048 | I | 0.114 |
| 3,4-Dimethoxydalbergione | 0 | 3238 | 0.9 | -0.014 | I | 0.497 |
| 7,2-Dihydroxyflavone | 0 | 5633 | 0.463 | -1.02 | I | 0.265 |
| 7,4-Dihydroxyflavone | 0 | 5907 | 0.666 | -0.559 | I | 0.364 |
| 7,8-Dihydroxyflavone | 0 | 5762 | 0.515 | -0.902 | I | 0.289 |
| Acetoxyphenol | 0 | 409 | -1 | -4.325 | I | 0.013 |
| Aconitine | 3 | 489529 | 2.79 | -1.043 | I | 0.260 |
| Agmatine | 0 | 114 | 0 | -2.047 | I | 0.114 |
| Alizarin | 0 | 17132 | 1.73 | 1.81 | A | 0.859 |
| Arecoline | 0 | 397 | -1.036 | -4.407 | I | 0.012 |
| Artemisinin | 0 | 15916 | 0.85 | -0.188 | I | 0.453 |
| Asarylaldehyde | 0 | 754 | -0.768 | -3.799 | I | 0.022 |
| Azelaicacid | 0 | 344 | -0.737 | -3.726 | I | 0.024 |
| Baicalein | 0 | 6474 | 0.661 | -0.573 | I | 0.360 |
| Betaine | 0 | 66 | 0 | -2.047 | I | 0.114 |
| Bicuculline (+) | 3 | 77053 | 2.106 | -0.641 | I | 0.345 |
| Biochanin A | 0 | 7728 | 0.873 | -0.096 | I | 0.476 |
| Brucine | 6 | 280357 | 2.176 | -4.469 | I | 0.011 |
| Caperatic acid | 0 | 2707 | 1.352 | 1.018 | A | 0.735 |
| Capsaicin | 0 | 2688 | 0.753 | -0.346 | I | 0.414 |
| Carnitine | 0 | 170 | 0 | -2.048 | I | 0.114 |
| Cevadine | 2 | 1106372 | 2.929 | -2.65 | I | 0.065 |
| Cineole | 0 | 636 | -1 | -4.326 | I | 0.013 |
| Citrinin | 0 | 3002 | -0.009 | -2.082 | I | 0.111 |
| Cotarnine | 1 | 4474 | 0.086 | -2.88 | I | 0.053 |
| Cotinine | 0 | 1114 | -0.596 | -3.409 | I | 0.032 |
| Crassin Acetate | 0 | 10720 | 1.227 | 0.695 | A | 0.667 |
| Daidzein | 0 | 5920 | 0.593 | -0.725 | I | 0.326 |
| Desoxypeganine | 1 | 2788 | -0.511 | -4.23 | I | 0.014 |
| Digitoxin | 1 | 1067756 | 2.987 | -1.327 | I | 0.208 |
| Digoxina | 1 | 1102711 | 3.046 | -1.359 | I | 0.203 |
| Epicatechin | 0 | 7642 | 0.595 | -0.729 | I | 0.325 |
| Esculetin | 0 | 1405 | -0.507 | -3.208 | I | 0.039 |
| Eugenol | 0 | 521 | -0.871 | -4.032 | I | 0.017 |
| Folic acid | 0 | 23186 | 2 | 2.395 | A | 0.916 |
| Formononetin | 0 | 6847 | 0.759 | -0.352 | I | 0.413 |
| Galanthamine | 2 | 17777 | 0.884 | -2.134 | I | 0.106 |
| Geranylgeranio | 0 | 194 | 0 | -2.048 | I | 0.114 |
| Harmaline | 2 | 4686 | 0.204 | -3.619 | I | 0.026 |
| Hinokitiol | 0 | 537 | -0.87 | -4.03 | I | 0.017 |
| Huperzinea | 0 | 6120 | 0.476 | -0.992 | I | 0.270 |
| Hydrocotarnine | 1 | 4474 | 0.086 | -2.88 | I | 0.053 |
| Inositol | 0 | 486 | -0.677 | -3.59 | I | 0.027 |
| Menadione | 0 | 1380 | -0.516 | -3.228 | I | 0.038 |
| Menthol | 0 | 399 | -1 | -4.325 | I | 0.013 |
| Morin | 0 | 8350 | 0.812 | -0.238 | I | 0.441 |
| Theobromine | 1 | 1297 | -0.554 | -4.321 | I | 0.013 |
| Colchiceine | 0 | 19381 | 1.604 | 1.512 | A | 0.819 |
| Deltaline | 4 | 273281 | 2.216 | -2.33 | I | 0.088 |
| 1r,9s-Hydrastine | 2 | 50420 | 1.998 | 0.247 | A | 0.561 |
| Melatonin | 1 | 2735 | 0.121 | -2.792 | I | 0.058 |
| Ajmaline | 5 | 79223 | 1.779 | -3.41 | I | 0.032 |
| Salsolidine | 0 | 1994 | -0.332 | -2.812 | I | 0.057 |
| Vincamine | 3 | 55671 | 1.602 | -1.686 | I | 0.156 |
| Vindoline | 4 | 82993 | 2.04 | -1.826 | I | 0.139 |
| Dihydrojasmonic Acid, Methyl Ester | 0 | 1100 | -0.219 | -2.551 | I | 0.072 |
| Penicillic Acid | 0 | 441 | -0.858 | -4.002 | I | 0.018 |
| Diprotin A | 0 | 2894 | 1.104 | 0.452 | A | 0.611 |
| Albizziine | 0 | 136 | 0 | -2.048 | I | 0.114 |
| D,L-Threo-3-hydroxyaspartic acid | 0 | 121 | 0 | -2.047 | I | 0.114 |
| Rutilantinone | 0 | 44306 | 1.733 | 1.687 | A | 0.844 |
| Dihydromyristicin | 1 | 1564 | -0.596 | -4.418 | I | 0.012 |
| Paeonol | 0 | 504 | -0.867 | -4.023 | I | 0.018 |
| Apiole | 1 | 2128 | 0 | -3.064 | I | 0.045 |
| 4-O-Methylphloracetophenone | 0 | 611 | -0.691 | -3.623 | I | 0.026 |
| Acetosyringone | 0 | 742 | -0.647 | -3.523 | I | 0.029 |
| Eupatoriochromene | 0 | 2292 | 0 | -2.058 | I | 0.113 |
| Inosine | 1 | 5729 | 0.593 | -1.731 | I | 0.150 |
| Salicin | 0 | 3461 | 0.606 | -0.684 | I | 0.335 |
| Khellin | 1 | 6541 | 0.531 | -1.876 | I | 0.133 |
| Citropten | 0 | 1988 | -0.137 | -2.368 | I | 0.086 |
| Pachyrrhizin | 2 | 35654 | 1.801 | -0.131 | I | 0.467 |
| Pteryxin | 0 | 17482 | 1.508 | 1.303 | A | 0.786 |
| Evernic Acid | 0 | 5305 | 0.836 | -0.169 | I | 0.458 |
| Norstictic Acid | 1 | 31955 | 1.439 | 0.07 | A | 0.517 |
| Colforsin | 0 | 17727 | 1.412 | 1.083 | A | 0.747 |
| Salvinorin B | 0 | 24940 | 1.557 | 1.379 | A | 0.799 |
| Salvinorin A | 0 | 31497 | 1.747 | 1.78 | A | 0.856 |
| Eupatorin | 0 | 11901 | 1.267 | 0.781 | A | 0.686 |
| Ligustilide | 1 | 1604 | -0.329 | -3.811 | I | 0.022 |
| Griseofulvin | 1 | 10354 | 1.181 | -0.415 | I | 0.398 |
| Ichthynone | 1 | 57899 | 2.038 | 1.31 | A | 0.787 |
| Protoporphyrin Ix | 0 | 193446 | 2.735 | 3.26 | A | 0.963 |
| Quassin | 0 | 36221 | 1.719 | 1.694 | A | 0.845 |
| Cryptotanshinone | 1 | 18983 | 0.976 | -0.923 | I | 0.284 |
| Pachyrrhizone | 2 | 98597 | 2.037 | 0.107 | A | 0.526 |
| Pseudo-Anisatin | 2 | 7744 | 0.395 | -3.199 | I | 0.039 |
| Frequentin | 0 | 1551 | 0.181 | -1.642 | I | 0.162 |
| Abscisic Acid (Cis,Trans; +/-) | 0 | 1582 | 0 | -2.054 | I | 0.114 |
| Chrysanthemic Acid, Ethyl Ester | 0 | 641 | -0.553 | -3.309 | I | 0.035 |
| Dihydrocelastryl Diacetate | 0 | 145616 | 2.515 | 2.986 | A | 0.952 |
| Beta-escin | 0 | 2437316 | 3.533 | -5.582 | I | 0.004 |
| Nonylphenol | 0 | 1178 | 0 | -2.052 | I | 0.114 |
| Norharman | 2 | 2805 | -0.234 | -4.607 | I | 0.010 |
| Octopamine | 0 | 397 | -1 | -4.325 | I | 0.013 |
| Patulin | 1 | 879 | -0.998 | -5.33 | I | 0.005 |
| Picrotin | 3 | 28432 | 0.552 | -3.947 | I | 0.019 |
| Picrotoxinin | 3 | 25486 | 0.545 | -3.949 | I | 0.019 |
| Pimpinellin | 1 | 5840 | 0.361 | -2.26 | I | 0.094 |
| Plectocomine Methyl Ether | 2 | 3979 | -0.013 | -4.11 | I | 0.016 |
| Purpurin | 0 | 6999 | 0.446 | -1.065 | I | 0.256 |
| Purpurogallin | 0 | 2433 | -0.324 | -2.796 | I | 0.058 |
| Pyridoxine | 0 | 506 | -0.775 | -3.813 | I | 0.022 |
| Pyrocatechuic acid | 0 | 399 | -1 | -4.325 | I | 0.013 |
| Reserpine | 2 | 280011 | 2.751 | 0.871 | A | 0.704 |
| Resveratrol | 0 | 2319 | 0.076 | -1.885 | I | 0.132 |
| Retinol | 0 | 2271 | 0.659 | -0.558 | I | 0.364 |
| Safrole | 1 | 1090 | -0.727 | -4.714 | I | 0.009 |
| Scopoletin | 0 | 1687 | -0.356 | -2.865 | I | 0.054 |
| Securinine | 2 | 7901 | 0.2 | -3.644 | I | 0.025 |
| Sennoside | 0 | 1234645 | 3.327 | -0.339 | I | 0.413 |
| Tannicacid | 0 | 1596253 | 3.956 | -0.624 | I | 0.345 |
| Theaflavindigallate | 0 | 802628 | 3.215 | 1.459 | A | 0.810 |
| Thiamine | 0 | 2646 | 0.263 | -1.461 | I | 0.188 |
| Trigonelline | 0 | 314 | 0 | -2.048 | I | 0.114 |
| Tryptamine | 2 | 13238 | 0.634 | -2.681 | I | 0.064 |
| Umbelliferone | 0 | 1163 | -0.793 | -3.857 | I | 0.021 |
| Ursodiol | 1 | 34454 | 1.694 | 0.638 | A | 0.654 |
| Usnic acid | 2 | 13147 | 1.065 | -1.7 | I | 0.155 |
| Xanthopterin | 0 | 1402 | -0.537 | -3.276 | I | 0.036 |
| Xanthurenicacid | 0 | 1964 | -0.23 | -2.58 | I | 0.070 |
| Yohimbine | 2 | 55192 | 1.739 | -0.365 | I | 0.410 |

DF: discriminant function value for each compound.

CLASS: classification of the model for ach compound.

P.(Activ): probability of a compounds for being active.

nR09: number of 9-membered rings

Wap: all path Wiener index

EEig11r: eigenvalue 11 from edge adj. matrix weighted by resonance integrals.
