# Supplementary material for: Novel Cancer Chemotherapy Hits by Molecular Topology: Dual Akt and Beta-Catenin Inhibitors
Source: PLoS One. 2015 Apr 24;10(4):e0124244. doi: 10.1371/journal.pone.0124244 (PMC4409212; doi:10.1371/journal.pone.0124244)
Supplement: S2 Table — (DOCX) [file pone.0124244.s002.docx]

**S2 Table. Compounds used in the *test set* and corresponding values of the DF_1_ to Akt natural inhibitors.**

| **COMPOUNDS** | **nR09** | **Wap** | **EEig11r** | **DF** | **CLASS** | **P. (Activ.)** |
| --- | --- | --- | --- | --- | --- | --- |
| **ACTIVE GROUP** | | | | | | |
| Betulinic acid [76] | 1 | 88997 | 2.169 | 1.46 | A | 0.811 |
| Calcitriol [77] | 1 | 19531 | 1.872 | 1.11 | A | 0.753 |
| Camphor [78] | 0 | 729 | -0.81 | -3.89 | I | 0.020 |
| Cholecalciferol [79] | 1 | 16108 | 1.797 | 0.96 | A | 0.723 |
| Cosmosiin [80] | 0 | 32090 | 2 | 2.35 | A | 0.913 |
| Embelin [81] | 0 | 2470 | 0.675 | -0.52 | I | 0.372 |
| Ergosterol [82] | 1 | 37919 | 1.937 | 1.18 | A | 0.764 |
| Fisetin [83] | 0 | 7447 | 0.799 | -0.26 | I | 0.434 |
| **INACTIVE GROUP** | | | | | | |
| 6,4-Dihydroxyflavone | 0 | 5907 | 0.471 | -1.00 | I | 0.268 |
| Anethole | 0 | 422 | -1 | -4.33 | I | 0.013 |
| Anisodamine | 0 | 6614 | 0.85 | -0.14 | I | 0.464 |
| Avocadene | 0 | 1233 | 0.446 | -1.04 | I | 0.262 |
| Canavanine | 0 | 247 | -0.978 | -4.27 | I | 0.014 |
| Cantharidin | 1 | 2566 | -0.37 | -3.91 | I | 0.020 |
| Cedrol | 0 | 3796 | -0.077 | -2.24 | I | 0.096 |
| Chrysanthemic acid | 0 | 414 | -0.847 | -3.98 | I | 0.018 |
| Chrysin | 0 | 5761 | 0.469 | -1.01 | I | 0.268 |
| Creatinine | 0 | 164 | 0 | -2.05 | I | 0.114 |
| Dantron | 0 | 6228 | 0.456 | -1.04 | I | 0.261 |
| Emodic acid | 0 | 10142 | 0.707 | -0.49 | I | 0.381 |
| Erysolin | 0 | 198 | 0 | -2.05 | I | 0.114 |
| Estragole | 0 | 506 | -0.891 | -4.08 | I | 0.017 |
| Fusidic acid | 1 | 62762 | 2.47 | 2.27 | A | 0.906 |
| Glutathione | 0 | 969 | 0.083 | -1.86 | I | 0.134 |
| Guvacine | 0 | 239 | 0 | -2.05 | I | 0.114 |
| Harmalol | 2 | 3979 | -0.188 | -4.51 | I | 0.011 |
| Harmane | 2 | 3352 | -0.193 | -4.52 | I | 0.011 |
| Icariin | 0 | 99894 | 3.001 | 4.31 | n.c. | 0.987 |
| Mimosine | 0 | 742 | -0.472 | -3.13 | I | 0.042 |
| Naringenin | 0 | 6688 | 0.723 | -0.43 | I | 0.393 |
| Lappaconitine | 3 | 391443 | 2.742 | -0.69 | I | 0.334 |
| Colchicine | 0 | 21224 | 1.622 | 1.54 | A | 0.824 |
| 1-Methylxanthine | 1 | 1059 | -0.685 | -4.62 | I | 0.01 |
| Piplartine | 0 | 4641 | 1.141 | 0.53 | A | 0.629 |
| Salsoline | 0 | 1685 | -0.418 | -3.01 | I | 0.047 |
| Strychnine | 6 | 192569 | 2.009 | -4.43 | I | 0.012 |
| Kinetin | 1 | 3422 | -0.081 | -3.26 | I | 0.037 |
| Kainic Acid | 0 | 851 | -0.15 | -2.39 | I | 0.084 |
| Physcion | 0 | 9087 | 0.681 | -0.54 | I | 0.368 |

DF: discriminant function value for each compound.

CLASS: classification of the model for ach compound.

P.(Activ): probability of a compounds for being active.

nR09: number of 9-membered rings

Wap: all path Wiener index

EEig11r: eigenvalue 11 from edge adj. matrix weighted by resonance integrals.
