# Supplementary material for: Novel Cancer Chemotherapy Hits by Molecular Topology: Dual Akt and Beta-Catenin Inhibitors
Source: PLoS One. 2015 Apr 24;10(4):e0124244. doi: 10.1371/journal.pone.0124244 (PMC4409212; doi:10.1371/journal.pone.0124244)
Supplement: S3 Table — (DOCX) [file pone.0124244.s003.docx]

**S3 Table. Compounds used in the *training set* and corresponding values of the DF_2_ to Akt inhibitors.**

| **COMPOUNDS** | **T(O..Br)** | **SRW08** | **MPC04** | **piPC02** | **piPC05** | **DF** | **CLASS** | **P. (Activ.)** |
| --- | --- | --- | --- | --- | --- | --- | --- | --- |
| **ACTIVE GROUP** | | | | | | | | |
| 1,3-Dihydro-1-(1-((4-(6-Phenyl-1h-Imidazo[4,5-G]Quinoxalin-7-Yl)Phenyl)Methyl)-4-Piperidinyl)-2h-Benzimidazol-2-One [86] | 0 | 9342 | 142 | 4.745 | 6.468 | 5.55 | A | 0.945 |
| 10-(4'-(N-Diethylamino)Butyl)-2-Chlorophenoxazine [87] | 0 | 4432 | 62 | 3.97 | 5.576 | 1.09 | I | 0.450 |
| 1l6-Hydroxymethyl-Chiro-Inositol-2-(R)-2-O-Methyl-3-O-Octadecyl-Sn-Glycerocarbonate [88] | 0 | 5016 | 63 | 3.932 | 4.331 | 1.95 | A | 0.620 |
| A-66 [85] | 0 | 4856 | 68 | 4.234 | 5.771 | 2.38 | A | 0.724 |
| AT7867 [85] | 0 | 5054 | 69 | 4.094 | 5.587 | 1.56 | A | 0.522 |
| Balanol [89] | 0 | 8466 | 115 | 4.585 | 5.994 | 3.41 | A | 0.721 |
| CCT128930 [85] | 0 | 5010 | 67 | 4.078 | 5.525 | 1.38 | I | 0.480 |
| Erlotinib [90] | 0 | 4758 | 68 | 4.19 | 5.835 | 2.19 | A | 0.691 |
| Gefitinib [91] | 0 | 5620 | 76 | 4.263 | 5.802 | 2.07 | A | 0.608 |
| GSK690693 [85] | 0 | 6136 | 88 | 4.29 | 5.858 | 2.76 | A | 0.726 |
| H-89 [92] | 26 | 4718 | 59 | 4.248 | 5.717 | 3.5 | A | 0.895 |
| Miltefosine [85] | 0 | 2768 | 25 | 3.555 | 3.296 | 0.65 | I | 0.461 |
| MK-2206 [85] | 0 | 7984 | 103 | 4.466 | 6.227 | 1.59 | A | 0.325 |
| Perifosine [85] | 0 | 3758 | 40 | 3.738 | 3.892 | 0.85 | I | 0.440 |
| PF-04691502 [85] | 0 | 6332 | 90 | 4.277 | 5.889 | 2.51 | A | 0.661 |
| SH-5 [93] | 0 | 5108 | 61 | 3.97 | 4.357 | 1.68 | A | 0.549 |
| SH-6 [93] | 0 | 4628 | 53 | 3.912 | 4.174 | 1.5 | A | 0.538 |
| Staurosporine [94] | 0 | 10764 | 185 | 4.595 | 6.617 | 7.4 | A | 0.986 |
| TIC10 [85] | 0 | 6190 | 91 | 4.277 | 5.694 | 3.26 | A | 0.812 |
| Triciribine [85] | 0 | 5676 | 86 | 4.025 | 5.684 | 2.04 | A | 0.598 |
| **INACTIVE GROUP** | | | | | | | | |
| Acarbose | 0 | 9606 | 122 | 4.304 | 5.182 | 1.72 | A | 0.256 |
| Acebutolol | 0 | 3276 | 40 | 3.807 | 4.949 | 0.44 | I | 0.374 |
| Aceclidine | 0 | 2362 | 29 | 3.045 | 3.738 | -1.94 | I | 0.068 |
| Acedapsone | 0 | 4168 | 48 | 4.127 | 5.468 | 0.86 | I | 0.413 |
| Acetaminophen | 0 | 1430 | 16 | 3.219 | 4.344 | -1.8 | I | 0.098 |
| Acetanilide | 0 | 1172 | 14 | 3.091 | 4.143 | -2.02 | I | 0.086 |
| Acetarsol | 0 | 2662 | 26 | 3.611 | 4.828 | -1.27 | I | 0.114 |
| Acetazolamide | 0 | 2134 | 22 | 3.555 | 4.22 | -0.07 | I | 0.333 |
| Acetriazoic acid | 0 | 2924 | 36 | 3.664 | 4.956 | -0.31 | I | 0.238 |
| Acetylcholine | 0 | 1098 | 6 | 2.708 | 1.946 | -1.6 | I | 0.128 |
| Acetylcysteine | 0 | 1046 | 9 | 2.773 | 2.565 | -1.74 | I | 0.115 |
| Acyclovir | 0 | 2782 | 40 | 3.555 | 4.883 | -0.04 | I | 0.300 |
| Adenosinephosphate | 0 | 4866 | 65 | 3.97 | 5.236 | 1.21 | I | 0.449 |
| Adiphenine | 0 | 3376 | 46 | 3.85 | 5.17 | 0.95 | I | 0.492 |
| Adrenalinebitartrate | 0 | 1872 | 19 | 3.258 | 4.466 | -2.24 | I | 0.058 |
| Aklomide | 0 | 2146 | 23 | 3.466 | 4.691 | -1.32 | I | 0.125 |
| Alaproclate | 0 | 2698 | 27 | 3.555 | 4.489 | -0.98 | I | 0.146 |
| Albendazole | 0 | 2810 | 40 | 3.689 | 5.094 | 0.38 | I | 0.393 |
| Albuterol | 0 | 2762 | 27 | 3.526 | 4.644 | -1.55 | I | 0.087 |
| Alendronate | 0 | 3134 | 19 | 3.332 | 2.485 | -0.91 | I | 0.139 |
| Alfluzosin | 0 | 4984 | 67 | 4.094 | 5.595 | 1.41 | I | 0.490 |
| Algesteroneacetophenide | 0 | 10344 | 143 | 4.357 | 5.7 | 2.57 | A | 0.393 |
| Aliskirenhemifumarate | 0 | 6018 | 64 | 4.205 | 5.069 | 0.53 | I | 0.227 |
| Allopurinol | 0 | 1834 | 25 | 3.296 | 4.635 | -1.39 | I | 0.127 |
| Alprenolol | 0 | 2228 | 27 | 3.466 | 4.575 | -0.74 | I | 0.199 |
| Althiazide | 0 | 4778 | 56 | 4.111 | 5.416 | 0.73 | I | 0.340 |
| Alverinecitrate | 0 | 2580 | 33 | 3.714 | 4.812 | 0.52 | I | 0.443 |
| Amantadine | 0 | 3326 | 42 | 3.091 | 4.007 | -2.26 | I | 0.038 |
| Amikacin | 0 | 8080 | 101 | 4.159 | 4.89 | 1.49 | I | 0.298 |
| Amiloride | 0 | 2490 | 28 | 3.584 | 4.779 | -0.74 | I | 0.188 |
| Aminocaproic acid | 0 | 560 | 5 | 2.398 | 1.792 | -2.34 | I | 0.076 |
| Aminoglutethimide | 0 | 3628 | 42 | 3.664 | 4.875 | -0.76 | I | 0.140 |
| Aminohippuric acid | 0 | 1876 | 20 | 3.434 | 4.533 | -1.12 | I | 0.158 |
| Aminosalicylate | 0 | 1762 | 19 | 3.296 | 4.443 | -1.74 | I | 0.095 |
| Amiodarone | 0 | 5582 | 80 | 4.248 | 5.781 | 2.64 | A | 0.735 |
| Amitraz | 0 | 3506 | 41 | 3.989 | 5.352 | 0.56 | I | 0.388 |
| Amitriptyline | 0 | 3738 | 55 | 3.932 | 5.583 | 1.26 | I | 0.545 |
| Amlodipine | 0 | 5006 | 73 | 4.094 | 5.583 | 2.21 | A | 0.680 |
| Amodiaquine | 0 | 4570 | 61 | 4.111 | 5.714 | 1.32 | I | 0.498 |
| Amoxapine | 0 | 4430 | 66 | 4.025 | 5.724 | 1.73 | A | 0.609 |
| Amoxicillin | 0 | 6534 | 76 | 4.043 | 5.182 | -0.07 | I | 0.122 |
| Amphotericinb | 0 | 10550 | 130 | 4.71 | 5.407 | 3.08 | A | 0.505 |
| Amprolium | 0 | 2930 | 37 | 3.761 | 5.17 | 0.05 | I | 0.310 |
| Ampyzinesulfate | 0 | 1158 | 12 | 2.944 | 4.043 | -3.01 | I | 0.034 |
| Anethole | 0 | 1362 | 16 | 3.178 | 4.443 | -2.09 | I | 0.077 |
| Aniracetam | 0 | 2710 | 35 | 3.584 | 4.745 | -0.16 | I | 0.279 |
| Anthralin | 0 | 3998 | 56 | 3.892 | 5.572 | 0.65 | I | 0.375 |
| Apomorphine | 0 | 5218 | 75 | 4.007 | 5.823 | 1.1 | I | 0.398 |
| Apramycin | 0 | 8304 | 107 | 4.111 | 4.942 | 1.47 | I | 0.281 |
| Aripiprazole | 0 | 5478 | 72 | 4.19 | 5.509 | 1.85 | A | 0.565 |
| Armodafinil | 0 | 3000 | 42 | 3.807 | 5.153 | 0.91 | I | 0.510 |
| Ascorbic acid | 0 | 2060 | 24 | 3.178 | 3.784 | -1.26 | I | 0.134 |
| Ascorbylpalmitate | 0 | 3430 | 43 | 3.784 | 4.22 | 1.64 | A | 0.656 |
| Atenolol | 0 | 2342 | 27 | 3.555 | 4.595 | -0.45 | I | 0.244 |
| Atomoxetine | 0 | 2796 | 37 | 3.714 | 5.03 | 0.27 | I | 0.366 |
| Atovaquone | 0 | 5714 | 78 | 4.205 | 5.765 | 1.86 | A | 0.550 |
| Atropineoxide | 0 | 4652 | 59 | 3.871 | 4.883 | 0.8 | I | 0.366 |
| Atropinesulfate | 0 | 3966 | 55 | 3.738 | 4.787 | 0.96 | I | 0.452 |
| Avobenzone | 0 | 4104 | 44 | 4.007 | 5.313 | -0.04 | I | 0.225 |
| Azaperone | 0 | 3860 | 49 | 3.951 | 5.165 | 1.03 | I | 0.478 |
| Azithromycin | 0 | 11160 | 129 | 4.454 | 5.056 | 0.73 | I | 0.075 |
| Aztreonam | 0 | 6606 | 63 | 4.22 | 4.934 | -0.46 | I | 0.084 |
| Bacampicillin | 0 | 7260 | 88 | 4.19 | 5.204 | 1 | I | 0.249 |
| Bacitracin | 0 | 15218 | 199 | 5.209 | 5.999 | 5.31 | A | 0.709 |
| Bekanamycin | 0 | 7102 | 88 | 3.97 | 4.71 | 0.79 | I | 0.219 |
| Benazepril | 0 | 5074 | 71 | 4.19 | 5.434 | 2.65 | A | 0.765 |
| Bendroflumethiazide | 0 | 6378 | 75 | 4.357 | 5.775 | 1.05 | I | 0.310 |
| Benserazide | 0 | 2788 | 28 | 3.611 | 4.585 | -0.83 | I | 0.162 |
| Benurestat | 0 | 1994 | 21 | 3.466 | 4.543 | -1.04 | I | 0.165 |
| Benzbromarone | 30 | 4644 | 65 | 4.094 | 5.7 | 3.83 | A | 0.923 |
| Benzethonium | 0 | 5102 | 55 | 4.094 | 5.165 | 0.27 | I | 0.228 |
| Benzocaine | 0 | 1620 | 18 | 3.258 | 4.431 | -1.81 | I | 0.092 |
| Benzoylpas | 0 | 3172 | 39 | 3.871 | 5.193 | 0.49 | I | 0.394 |
| Benzoylperoxide | 0 | 2630 | 32 | 3.761 | 4.997 | 0.26 | I | 0.377 |
| Benzthiazide | 0 | 5592 | 67 | 4.344 | 5.787 | 1.43 | I | 0.451 |
| Benzylbenzoat | 0 | 2246 | 28 | 3.638 | 4.89 | -0.1 | I | 0.319 |
| Bepheniumhydroxynapthoate | 0 | 2824 | 33 | 3.714 | 4.844 | -0.02 | I | 0.301 |
| Bepridil | 0 | 3958 | 56 | 3.951 | 5.112 | 1.89 | A | 0.676 |
| Beta-Carotene | 0 | 6498 | 70 | 4.466 | 5.46 | 1.35 | I | 0.368 |
| Betahistine | 0 | 1048 | 13 | 2.944 | 4.06 | -2.68 | I | 0.048 |
| Betaine | 0 | 974 | 6 | 2.565 | 0 | 1.09 | I | 0.690 |
| Beta-Propiolactone | 0 | 866 | 2 | 2.197 | 0 | -1.55 | I | 0.142 |
| Bethanechol | 0 | 1364 | 11 | 2.833 | 1.946 | -0.66 | I | 0.257 |
| Bezafibrate | 0 | 4172 | 43 | 4.043 | 5.22 | 0.07 | I | 0.241 |
| Bifonazole | 0 | 4350 | 65 | 4.143 | 5.714 | 2.51 | A | 0.776 |
| Biotin | 0 | 2502 | 37 | 3.332 | 3.85 | 0.48 | I | 0.440 |
| Bisacodyl | 0 | 4558 | 64 | 4.205 | 5.72 | 2.33 | A | 0.732 |
| Bisoprololfumarate | 0 | 2614 | 30 | 3.611 | 4.585 | -0.21 | I | 0.274 |
| Bithionate | 0 | 3668 | 44 | 3.912 | 5.293 | 0.27 | I | 0.310 |
| Bromhexine | 0 | 3118 | 38 | 3.611 | 4.762 | -0.43 | I | 0.208 |
| Bromindione | 14 | 3852 | 56 | 3.912 | 5.525 | 2.17 | A | 0.742 |
| Bromocriptinemesylate | 60 | 11998 | 179 | 4.625 | 6.146 | 9.48 | N.C. | 0.998 |
| Bromperidol | 19 | 4788 | 55 | 4.06 | 5.293 | 1.85 | A | 0.614 |
| Brompheniraminemaleate | 0 | 2920 | 38 | 3.738 | 5.13 | 0.13 | I | 0.328 |
| Brucine | 14 | 3852 | 56 | 3.912 | 5.525 | 2.17 | A | 0.742 |
| Bumetanide | 0 | 4396 | 55 | 4.143 | 5.613 | 1.22 | I | 0.485 |
| Bupivacaine | 0 | 3472 | 45 | 3.714 | 4.812 | 0.38 | I | 0.348 |
| Bupropion | 0 | 2636 | 27 | 3.526 | 4.575 | -1.18 | I | 0.125 |
| Buspirone | 0 | 5362 | 72 | 4.007 | 4.97 | 1.86 | A | 0.576 |
| Butacaine | 0 | 2556 | 31 | 3.611 | 4.605 | 0.01 | I | 0.324 |
| Butamben | 0 | 1760 | 20 | 3.332 | 4.477 | -1.43 | I | 0.125 |
| Butoconazole | 0 | 4022 | 53 | 4.06 | 5.231 | 1.83 | A | 0.659 |
| Caffeine | 0 | 3242 | 42 | 3.526 | 4.754 | -0.65 | I | 0.170 |
| Candesartan | 0 | 8432 | 128 | 4.673 | 6.252 | 5.37 | A | 0.949 |
| Canrenoic acid | 0 | 7406 | 92 | 4.043 | 5.268 | 0.23 | I | 0.128 |
| Canrenone | 0 | 7754 | 104 | 4.06 | 5.361 | 1.13 | I | 0.245 |
| Capobenic acid | 0 | 3234 | 41 | 3.784 | 5.011 | 0.41 | I | 0.370 |
| Capsaicin | 0 | 2688 | 31 | 3.714 | 4.7 | 0.22 | I | 0.364 |
| Carbachol | 0 | 1098 | 6 | 2.708 | 1.946 | -1.6 | I | 0.128 |
| Carbamazepine | 0 | 3608 | 54 | 3.892 | 5.602 | 1.1 | I | 0.514 |
| Carbenicillin | 0 | 6692 | 82 | 4.078 | 5.187 | 0.65 | I | 0.215 |
| Carbinoxamine | 0 | 2990 | 39 | 3.761 | 5.13 | 0.27 | I | 0.355 |
| Carisoprodol | 0 | 2218 | 21 | 3.296 | 3.332 | -0.48 | I | 0.245 |
| Cefaclor | 0 | 5908 | 68 | 4.025 | 5.204 | -0.06 | I | 0.144 |
| Cefadroxil | 0 | 6166 | 70 | 4.078 | 5.278 | -0.1 | I | 0.131 |
| Cefamandole | 0 | 7136 | 87 | 4.277 | 5.442 | 1.25 | I | 0.305 |
| Cefdinir | 0 | 6252 | 77 | 4.127 | 5.257 | 1.03 | I | 0.312 |
| Cefditorin | 0 | 8824 | 105 | 4.543 | 5.649 | 1.65 | A | 0.286 |
| Cefonicid | 0 | 7972 | 96 | 4.454 | 5.565 | 1.71 | A | 0.353 |
| Cefoperazone | 0 | 9952 | 124 | 4.605 | 5.743 | 2.23 | A | 0.340 |
| Cefotaxime | 0 | 6664 | 83 | 4.22 | 5.333 | 1.48 | I | 0.387 |
| Cefprozil | 0 | 6378 | 75 | 4.143 | 5.429 | 0.31 | I | 0.176 |
| Cefsulodin | 0 | 8526 | 103 | 4.533 | 5.79 | 1.67 | A | 0.308 |
| Ceftibuten | 0 | 6106 | 73 | 4.143 | 5.204 | 0.96 | I | 0.308 |
| Ceftriaxone | 0 | 8218 | 102 | 4.443 | 5.617 | 1.88 | A | 0.376 |
| Cephalexin | 0 | 5908 | 68 | 4.025 | 5.204 | -0.06 | I | 0.144 |
| Cephalothin | 0 | 5816 | 71 | 4.06 | 5.063 | 0.99 | I | 0.332 |
| Cephradine | 0 | 5908 | 68 | 3.989 | 5.056 | -0.04 | I | 0.148 |
| Cetirizine | 0 | 4362 | 60 | 4.025 | 5.323 | 1.73 | A | 0.613 |
| Chenodiol | 0 | 7402 | 99 | 3.951 | 4.934 | 1.19 | I | 0.278 |
| Chloramphenicol | 0 | 3084 | 33 | 3.689 | 4.754 | -0.54 | I | 0.191 |
| Chlorcyclizine | 0 | 3762 | 52 | 3.85 | 5.273 | 0.82 | I | 0.433 |
| Chlormadinoneacetate | 0 | 8282 | 109 | 4.159 | 5.442 | 1.24 | I | 0.237 |
| Chlorocresol | 0 | 1362 | 13 | 3.091 | 4.205 | -2.64 | I | 0.046 |
| Chloroguanide | 0 | 2210 | 26 | 3.555 | 4.477 | -0.12 | I | 0.318 |
| Chlorotrianisene | 0 | 4782 | 64 | 4.22 | 5.872 | 1.72 | A | 0.581 |
| Chloroxine | 0 | 2708 | 34 | 3.584 | 5.159 | -1 | I | 0.143 |
| Chlorpheniramine | 0 | 2920 | 38 | 3.738 | 5.13 | 0.13 | I | 0.328 |
| Chlorpropamide | 0 | 2670 | 28 | 3.714 | 4.71 | -0.17 | I | 0.280 |
| Chlorprothixene | 0 | 4118 | 58 | 3.97 | 5.707 | 0.94 | I | 0.436 |
| Chlortetracycline | 0 | 9872 | 134 | 4.443 | 6.04 | 2.23 | A | 0.347 |
| Chlorthalidone | 0 | 5456 | 69 | 4.174 | 5.704 | 1.05 | I | 0.370 |
| Chlorzoxazone | 0 | 2020 | 28 | 3.401 | 4.844 | -1.06 | I | 0.162 |
| Cholecalciferol | 0 | 5552 | 72 | 3.951 | 4.913 | 1.23 | I | 0.406 |
| Choline | 0 | 772 | 3 | 2.197 | 0 | -1.22 | I | 0.190 |
| Ciclopirox | 0 | 2744 | 35 | 3.434 | 4.533 | -0.81 | I | 0.167 |
| Cilostazol | 0 | 4700 | 67 | 4.043 | 5.252 | 2.25 | A | 0.707 |
| Cimetidine | 0 | 1898 | 24 | 3.466 | 3.829 | 0.79 | I | 0.558 |
| Cinchonine | 0 | 5146 | 71 | 3.932 | 5.481 | 0.82 | I | 0.336 |
| Ciprofloxacin | 0 | 5534 | 74 | 4.06 | 5.609 | 1.03 | I | 0.360 |
| Citalopram | 0 | 4888 | 67 | 4.043 | 5.609 | 1.26 | I | 0.460 |
| Clarithromycin | 0 | 11352 | 131 | 4.477 | 5.13 | 0.63 | I | 0.065 |
| Clavulanate | 0 | 3502 | 42 | 3.401 | 4.369 | -1.29 | I | 0.091 |
| Clemastine | 0 | 4492 | 56 | 3.951 | 5.252 | 0.58 | I | 0.326 |
| Clofazimine | 0 | 6918 | 100 | 4.5 | 6.277 | 3.43 | A | 0.806 |
| Clofibrate | 0 | 2584 | 25 | 3.497 | 4.5 | -1.4 | I | 0.104 |
| Clomiphenecitrate | 0 | 4662 | 64 | 4.205 | 5.787 | 2.01 | A | 0.658 |
| Clomipramine | 0 | 4004 | 58 | 3.932 | 5.476 | 1.33 | I | 0.541 |
| Clonidine | 0 | 2286 | 30 | 3.497 | 4.635 | -0.35 | I | 0.265 |
| Clopidogrel | 0 | 4054 | 58 | 3.892 | 5.112 | 1.6 | A | 0.604 |
| Clopidol | 0 | 2094 | 20 | 3.332 | 4.489 | -2.12 | I | 0.061 |
| Clotrimazole | 0 | 5492 | 84 | 4.19 | 5.894 | 2.81 | A | 0.772 |
| Cloxyquin | 0 | 2370 | 30 | 3.497 | 5.063 | -1.26 | I | 0.125 |
| Coenzymeb12 | 0 | 26688 | 396 | 5.529 | 7.01 | 9.63 | N.C. | 0.869 |
| Colistimethate | 0 | 15670 | 172 | 5.328 | 5.642 | 2.06 | A | 0.076 |
| Colistin | 0 | 11510 | 146 | 4.868 | 5.485 | 4.21 | A | 0.704 |
| Cotinine | 0 | 2256 | 30 | 3.367 | 4.511 | -0.9 | I | 0.175 |
| Cresol | 0 | 1426 | 13 | 3.091 | 4.111 | -2.61 | I | 0.046 |
| Cromolyn | 0 | 6854 | 93 | 4.454 | 5.984 | 2.82 | A | 0.696 |
| Crotamiton | 0 | 2226 | 28 | 3.466 | 4.615 | -0.67 | I | 0.211 |
| Cyclizine | 0 | 3504 | 50 | 3.784 | 5.198 | 0.78 | I | 0.441 |
| Cyclobenzaprine | 0 | 3552 | 54 | 3.932 | 5.69 | 1.32 | I | 0.571 |
| Cycloserine | 0 | 898 | 9 | 2.485 | 1.946 | -2.18 | I | 0.080 |
| Cyclosporine | 0 | 14350 | 177 | 4.984 | 5.645 | 3.23 | A | 0.281 |
| Cyclothiazide | 0 | 6326 | 82 | 4.263 | 5.609 | 1.81 | A | 0.494 |
| Cyproheptadine | 0 | 4510 | 68 | 4.06 | 5.808 | 1.92 | A | 0.647 |
| Cyproterone | 0 | 8980 | 113 | 4.143 | 5.472 | 0.24 | I | 0.085 |
| Dantrolene | 0 | 3990 | 54 | 4.06 | 5.338 | 1.84 | A | 0.665 |
| Dapsone | 0 | 3372 | 36 | 3.912 | 5.303 | -0.25 | I | 0.225 |
| Debrisoquinsulfate | 0 | 2344 | 29 | 3.401 | 4.673 | -1.27 | I | 0.124 |
| Decoquinate | 0 | 4546 | 63 | 4.06 | 5.624 | 1.48 | I | 0.539 |
| Demeclocycline | 0 | 9162 | 127 | 4.407 | 5.984 | 2.57 | A | 0.477 |
| Denatoniumbenzoate | 0 | 4146 | 50 | 3.97 | 5.159 | 0.73 | I | 0.383 |
| Desipramine | 0 | 3552 | 54 | 3.829 | 5.425 | 1.13 | I | 0.524 |
| Dexlansoprazole | 0 | 4714 | 59 | 4.127 | 5.541 | 1.15 | I | 0.446 |
| Dexpanthenol | 0 | 1942 | 14 | 2.996 | 2.565 | -1.45 | I | 0.118 |
| Dexpropranolol | 0 | 2952 | 38 | 3.689 | 5.165 | -0.3 | I | 0.239 |
| Dextromethorphan | 0 | 5498 | 77 | 3.807 | 5.394 | 0.3 | I | 0.215 |
| Dibenzothiophene | 0 | 2746 | 42 | 3.638 | 5.371 | -0.01 | I | 0.309 |
| Dibucaine | 0 | 3824 | 52 | 3.912 | 5.472 | 0.75 | I | 0.410 |
| Dicumarol | 0 | 5684 | 78 | 4.248 | 5.905 | 1.95 | A | 0.574 |
| Dicyclomine | 0 | 3814 | 47 | 3.497 | 4.078 | -0.13 | I | 0.225 |
| Diethylcarbamazine | 0 | 2060 | 23 | 3.045 | 3.296 | -1.4 | I | 0.120 |
| Diethylstilbestrol | 0 | 3370 | 44 | 3.892 | 5.416 | 0.53 | I | 0.389 |
| Diethyltoluamide | 0 | 2068 | 24 | 3.367 | 4.511 | -1.34 | I | 0.125 |
| Digitoxin | 0 | 14058 | 187 | 4.625 | 5.595 | 3.02 | A | 0.258 |
| Digoxin | 0 | 14500 | 194 | 4.644 | 5.638 | 3.14 | A | 0.256 |
| Dihydroergotamine | 0 | 11736 | 176 | 4.654 | 6.131 | 5.42 | A | 0.883 |
| Dihydrostreptomycin | 0 | 8656 | 113 | 4.22 | 5.017 | 2.15 | A | 0.409 |
| Diloxanide | 0 | 2184 | 22 | 3.401 | 4.489 | -1.6 | I | 0.097 |
| Diperodon | 0 | 4058 | 55 | 4.06 | 5.112 | 2.23 | A | 0.741 |
| Dipyridamole | 0 | 6606 | 100 | 4.234 | 5.927 | 2.99 | A | 0.746 |
| Dipyrone | 0 | 4224 | 56 | 3.989 | 5.088 | 1.63 | A | 0.600 |
| Dirithromycin | 0 | 12410 | 153 | 4.554 | 5.283 | 1.75 | A | 0.135 |
| Disopyramide | 0 | 4748 | 59 | 3.97 | 5.298 | 0.52 | I | 0.297 |
| Disulfiram | 0 | 1862 | 18 | 3.135 | 2.89 | -0.43 | I | 0.275 |
| D-Lactitol | 0 | 4038 | 46 | 3.497 | 3.989 | -0.56 | I | 0.150 |
| Dobutamine | 0 | 3230 | 37 | 3.871 | 5.075 | 0.3 | I | 0.346 |
| Docosanol | 0 | 1424 | 19 | 3.091 | 2.944 | 0.22 | I | 0.451 |
| Domperidone | 0 | 6264 | 94 | 4.263 | 5.684 | 3.45 | A | 0.837 |
| Donepezil | 0 | 5322 | 76 | 4.143 | 5.557 | 2.34 | A | 0.688 |
| Dopamine | 0 | 1566 | 17 | 3.178 | 4.369 | -2.24 | I | 0.063 |
| Doxazosin | 0 | 7054 | 97 | 4.382 | 5.945 | 2.58 | A | 0.630 |
| Doxepin | 0 | 3738 | 55 | 3.932 | 5.583 | 1.26 | I | 0.545 |
| Doxylamine | 0 | 3474 | 42 | 3.761 | 5.124 | -0.27 | I | 0.217 |
| Droperidol | 0 | 5258 | 74 | 4.205 | 5.595 | 2.51 | A | 0.729 |
| Duloxetine | 0 | 3550 | 52 | 3.892 | 5.308 | 1.45 | I | 0.603 |
| Dyphylline | 0 | 3766 | 50 | 3.664 | 4.92 | -0.02 | I | 0.247 |
| Edoxudine | 0 | 3386 | 45 | 3.555 | 4.477 | 0.13 | I | 0.299 |
| Edrophonium | 0 | 2124 | 19 | 3.258 | 4.394 | -2.62 | I | 0.038 |
| Eletriptan | 0 | 5304 | 70 | 4.248 | 5.591 | 2.15 | A | 0.648 |
| Enalapril | 0 | 4148 | 54 | 3.932 | 4.754 | 1.73 | A | 0.627 |
| Enilconazole | 0 | 2888 | 40 | 3.714 | 4.844 | 0.81 | I | 0.493 |
| Enoxacin | 0 | 4882 | 67 | 3.989 | 5.576 | 0.99 | I | 0.394 |
| Enrofloxacin | 0 | 5918 | 78 | 4.111 | 5.67 | 1.03 | I | 0.333 |
| Equilin | 0 | 5390 | 73 | 3.932 | 5.416 | 0.71 | I | 0.299 |
| Ergonovine | 0 | 5502 | 81 | 4.06 | 5.645 | 1.99 | A | 0.598 |
| Ergotamine | 0 | 11736 | 176 | 4.682 | 6.194 | 5.49 | A | 0.890 |
| Erythromycin | 0 | 11154 | 128 | 4.466 | 5.1 | 0.6 | I | 0.067 |
| Erythrosine | 0 | 8350 | 121 | 4.454 | 6.33 | 3.07 | A | 0.656 |
| Escitalopram | 0 | 4888 | 67 | 4.043 | 5.609 | 1.26 | I | 0.460 |
| Estradiol | 0 | 5390 | 73 | 3.829 | 5.209 | 0.42 | I | 0.242 |
| Estrone | 0 | 5390 | 73 | 3.871 | 5.242 | 0.63 | I | 0.282 |
| Estropipate | 0 | 6218 | 81 | 4.127 | 5.38 | 1.43 | I | 0.408 |
| Ethambutol | 0 | 1234 | 12 | 2.708 | 2.303 | -1.66 | I | 0.117 |
| Ethamivan | 0 | 2524 | 29 | 3.497 | 4.727 | -1.13 | I | 0.135 |
| Ethinylestradiol | 0 | 6282 | 81 | 3.951 | 5.303 | 0.34 | I | 0.184 |
| Ethionamide | 0 | 1550 | 17 | 3.219 | 4.443 | -2.07 | I | 0.074 |
| Ethosuximide | 0 | 1936 | 19 | 2.996 | 3.219 | -1.87 | I | 0.080 |
| Ethoxzolamide | 0 | 3118 | 38 | 3.807 | 5.209 | 0.03 | I | 0.295 |
| Ethynodioldiacetate | 0 | 7182 | 98 | 4.06 | 5.159 | 1.79 | A | 0.428 |
| Etidronate | 0 | 2756 | 9 | 3.219 | 0 | 2.02 | A | 0.772 |
| Etomidate | 0 | 2930 | 41 | 3.689 | 4.89 | 0.63 | I | 0.444 |
| Eucatropine | 0 | 4100 | 45 | 3.738 | 4.654 | -0.45 | I | 0.162 |
| Eugenol | 0 | 1700 | 20 | 3.258 | 4.511 | -1.84 | I | 0.089 |
| Evansblue | 0 | 14078 | 174 | 5.22 | 6.899 | 2.69 | A | 0.198 |
| Ezetimibe | 0 | 6774 | 80 | 4.304 | 5.753 | 0.65 | I | 0.212 |
| Famotidine | 0 | 2660 | 31 | 3.85 | 4.277 | 1.86 | A | 0.747 |
| Febuxostat | 0 | 3774 | 50 | 3.97 | 5.389 | 1.08 | I | 0.495 |
| Felbamate | 0 | 2086 | 25 | 3.497 | 4.443 | -0.31 | I | 0.285 |
| Fenbendazole | 0 | 3562 | 51 | 3.97 | 5.438 | 1.55 | A | 0.627 |
| Fenoterol | 0 | 3434 | 42 | 3.892 | 5.124 | 0.63 | I | 0.408 |
| Fexofenadine | 0 | 7556 | 89 | 4.407 | 5.737 | 0.99 | I | 0.231 |
| Fipronil | 0 | 5874 | 70 | 4.174 | 5.635 | 0.47 | I | 0.225 |
| Florfenicol | 0 | 3594 | 35 | 3.871 | 4.852 | -0.32 | I | 0.204 |
| Fluconazole | 0 | 4136 | 57 | 3.951 | 5.081 | 1.72 | A | 0.627 |
| Flucytosine | 0 | 1362 | 13 | 3.045 | 3.892 | -2.39 | I | 0.058 |
| Fludrocortisone | 0 | 8888 | 109 | 4.078 | 5.22 | -0.1 | I | 0.064 |
| Flumequine | 0 | 4610 | 64 | 3.912 | 5.576 | 0.64 | I | 0.332 |
| Flunarizine | 0 | 5178 | 71 | 4.248 | 5.666 | 2.41 | A | 0.712 |
| Fluorescein | 0 | 6870 | 105 | 4.304 | 6.182 | 3.15 | A | 0.761 |
| Fluoxetine | 0 | 3618 | 42 | 3.85 | 5.153 | -0.05 | I | 0.249 |
| Fluphenazine | 0 | 6102 | 80 | 4.174 | 5.72 | 1.24 | I | 0.370 |
| Flurothyl | 0 | 1520 | 7 | 2.773 | 1.946 | -1.9 | I | 0.088 |
| Fluvastatin | 0 | 5524 | 82 | 4.248 | 5.897 | 2.83 | A | 0.773 |
| Fluvoxamine | 0 | 3076 | 34 | 3.689 | 4.875 | -0.6 | I | 0.184 |
| Folicacid | 0 | 5536 | 70 | 4.331 | 5.69 | 2.04 | A | 0.605 |
| Fomepizole | 0 | 592 | 7 | 2.565 | 1.946 | -1.34 | I | 0.180 |
| Fosfomycincalcium | 0 | 1764 | 10 | 2.833 | 1.609 | -1.02 | I | 0.177 |
| Furazolidone | 0 | 2446 | 32 | 3.584 | 4.477 | 0.41 | I | 0.426 |
| Furosemide | 0 | 3924 | 47 | 4.043 | 5.242 | 1.07 | I | 0.483 |
| Fusidicacid | 0 | 9944 | 135 | 4.331 | 5.497 | 2.46 | A | 0.394 |
| Galanthamine | 0 | 5412 | 82 | 3.912 | 5.595 | 1.47 | I | 0.475 |
| Gallaminetriethiodide | 0 | 6024 | 59 | 4.06 | 4.875 | -0.75 | I | 0.076 |
| Gemfibrozil | 0 | 2904 | 25 | 3.611 | 4.511 | -1.35 | I | 0.100 |
| Gemifloxacinmesylate | 0 | 6262 | 87 | 4.205 | 5.746 | 2.03 | A | 0.553 |
| Gentamicin | 0 | 7022 | 85 | 3.97 | 4.663 | 0.62 | I | 0.194 |
| Gluceptate | 0 | 2176 | 18 | 3.091 | 2.833 | -1.23 | I | 0.134 |
| Gluconolactone | 0 | 2228 | 23 | 2.996 | 3.401 | -2.22 | I | 0.054 |
| Gramicidin | 0 | 22970 | 310 | 5.617 | 6.727 | 6.32 | A | 0.414 |
| Guaifenesin | 0 | 1816 | 21 | 3.258 | 4.382 | -1.71 | I | 0.096 |
| Guanethidine | 0 | 1348 | 17 | 2.944 | 2.996 | -0.91 | I | 0.213 |
| Guanfacine | 0 | 2230 | 26 | 3.526 | 4.543 | -0.45 | I | 0.249 |
| Halazone | 0 | 2838 | 24 | 3.689 | 4.745 | -1.27 | I | 0.110 |
| Haloperidol | 0 | 4788 | 55 | 4.06 | 5.293 | 0.46 | I | 0.283 |
| Hetacillin | 0 | 7984 | 104 | 4.143 | 5.398 | 1.12 | I | 0.232 |
| Hexachlorophene | 0 | 4472 | 52 | 4.025 | 5.476 | 0.15 | I | 0.240 |
| Hexylresorcinol | 0 | 1784 | 21 | 3.296 | 4.431 | -1.49 | I | 0.118 |
| Histamine | 0 | 796 | 11 | 2.708 | 2.708 | -1.61 | I | 0.136 |
| Homatropine | 0 | 3824 | 51 | 3.714 | 4.745 | 0.62 | I | 0.378 |
| Hydralazine | 0 | 2166 | 29 | 3.434 | 4.99 | -1.25 | I | 0.132 |
| Hydrastine | 0 | 6760 | 105 | 4.263 | 5.948 | 3.52 | A | 0.826 |
| Hydrochlorothiazide | 0 | 4152 | 45 | 3.989 | 5.308 | -0.11 | I | 0.212 |
| Hydroflumethiazide | 0 | 5070 | 54 | 4.094 | 5.485 | -0.36 | I | 0.138 |
| Hydroquinone | 0 | 1032 | 10 | 2.944 | 4.078 | -3.09 | I | 0.032 |
| Hydroxyamphetamine | 0 | 1430 | 16 | 3.135 | 4.29 | -2.23 | I | 0.066 |
| Hydroxychloroquine | 0 | 3548 | 47 | 3.829 | 5.247 | 0.48 | I | 0.365 |
| Hydroxyzinepamoate | 0 | 10840 | 154 | 4.898 | 6.569 | 4.98 | A | 0.862 |
| Hyoscyamine | 0 | 3966 | 55 | 3.738 | 4.787 | 0.96 | I | 0.452 |
| Imipramine | 0 | 3738 | 55 | 3.871 | 5.429 | 1.15 | I | 0.515 |
| Inositol | 0 | 2496 | 24 | 2.944 | 3.219 | -2.63 | I | 0.034 |
| Iodipamide | 0 | 6286 | 80 | 4.394 | 5.697 | 2.29 | A | 0.613 |
| Iodoquinol | 0 | 2708 | 34 | 3.584 | 5.159 | -1 | I | 0.143 |
| Iopanicacid | 0 | 3010 | 33 | 3.638 | 4.812 | -0.81 | I | 0.156 |
| Isoniazid | 0 | 1284 | 14 | 3.091 | 4.205 | -2.35 | I | 0.062 |
| Isopropamide | 0 | 5458 | 60 | 4.025 | 5.313 | -0.45 | I | 0.116 |
| Isoproterenol | 0 | 2206 | 23 | 3.401 | 4.543 | -1.6 | I | 0.096 |
| Isosorbide | 0 | 1958 | 28 | 2.833 | 3.258 | -1.77 | I | 0.088 |
| Isosorbidemononitrate | 0 | 2364 | 35 | 3.135 | 3.664 | -0.43 | I | 0.247 |
| Kanamycinasulfate | 0 | 7102 | 88 | 3.97 | 4.71 | 0.79 | I | 0.219 |
| Ketanserin | 0 | 5732 | 75 | 4.22 | 5.517 | 1.93 | A | 0.566 |
| Ketoconazole | 0 | 7100 | 98 | 4.394 | 5.628 | 3.24 | A | 0.766 |
| Ketotifen | 0 | 4642 | 72 | 4.043 | 5.727 | 2.23 | A | 0.708 |
| Labetalol | 0 | 3686 | 44 | 3.951 | 5.193 | 0.65 | I | 0.396 |
| Lactulose | 0 | 4960 | 60 | 3.611 | 4.277 | -0.27 | I | 0.153 |
| Levamisole | 0 | 2600 | 40 | 3.497 | 4.779 | 0.14 | I | 0.351 |
| Levobunolol | 0 | 3712 | 43 | 3.761 | 4.99 | -0.38 | I | 0.188 |
| Levocarnitine | 0 | 1364 | 11 | 2.833 | 1.946 | -0.66 | I | 0.257 |
| Levodopa | 0 | 2128 | 21 | 3.434 | 4.522 | -1.47 | I | 0.110 |
| Levofloxacin | 0 | 6330 | 90 | 4.143 | 5.817 | 1.79 | A | 0.489 |
| Levonordefrin | 0 | 2130 | 21 | 3.332 | 4.511 | -2.1 | I | 0.062 |
| Lincomycin | 0 | 5040 | 66 | 3.761 | 4.431 | 1.07 | I | 0.403 |
| Lindane | 0 | 2496 | 24 | 2.944 | 3.219 | -2.63 | I | 0.034 |
| Lobendazole | 0 | 2348 | 34 | 3.555 | 4.875 | 0.02 | I | 0.340 |
| Loperamide | 0 | 7222 | 91 | 4.357 | 5.768 | 1.57 | A | 0.371 |
| Loratadine | 0 | 5364 | 79 | 4.19 | 5.808 | 2.53 | A | 0.726 |
| Losartan | 0 | 5486 | 83 | 4.304 | 5.799 | 3.56 | A | 0.878 |
| Lovastatin | 0 | 5526 | 74 | 3.989 | 4.977 | 1.69 | A | 0.520 |
| Loxapinesuccinate | 0 | 4688 | 68 | 4.06 | 5.737 | 1.68 | A | 0.580 |
| Mafenide | 0 | 2052 | 18 | 3.497 | 4.554 | -1.39 | I | 0.120 |
| Malathion | 0 | 2520 | 25 | 3.434 | 3.689 | -0.28 | I | 0.266 |
| Maprotiline | 0 | 5636 | 81 | 3.951 | 5.79 | 0.79 | I | 0.301 |
| Mebendazole | 0 | 3948 | 55 | 4.043 | 5.553 | 1.59 | A | 0.609 |
| Mebeverine | 0 | 4596 | 57 | 4.111 | 5.347 | 1.35 | I | 0.503 |
| Meclizine | 0 | 5054 | 71 | 4.19 | 5.602 | 2.41 | A | 0.719 |
| Meclocycline | 0 | 9604 | 136 | 4.454 | 6.089 | 3.03 | A | 0.560 |
| Mefexamide | 0 | 2384 | 27 | 3.555 | 4.533 | -0.42 | I | 0.246 |
| Memantine | 0 | 4626 | 57 | 3.332 | 4.205 | -1.63 | I | 0.049 |
| Menadione | 0 | 2708 | 34 | 3.584 | 5.153 | -0.99 | I | 0.144 |
| Menthol | 0 | 1762 | 19 | 2.773 | 2.944 | -2.45 | I | 0.049 |
| Mepenzolate | 0 | 5234 | 63 | 4.025 | 5.333 | 0.38 | I | 0.242 |
| Mephenterminesulfate | 0 | 1784 | 17 | 3.178 | 4.19 | -2.37 | I | 0.053 |
| Mesna | 0 | 772 | 3 | 2.773 | 0 | 2.38 | A | 0.896 |
| Metaproterenol | 0 | 2158 | 25 | 3.401 | 4.522 | -1.19 | I | 0.140 |
| Metaraminol | 0 | 1808 | 19 | 3.219 | 4.344 | -2.15 | I | 0.065 |
| Metaxalone | 0 | 2470 | 33 | 3.555 | 4.554 | 0.19 | I | 0.371 |
| Metformin | 0 | 912 | 6 | 2.708 | 1.946 | -1.23 | I | 0.183 |
| Methacholine | 0 | 1364 | 11 | 2.833 | 1.946 | -0.66 | I | 0.257 |
| Methacycline | 0 | 9258 | 131 | 4.419 | 6.011 | 2.95 | A | 0.565 |
| Methapyrilene | 0 | 2470 | 34 | 3.611 | 4.635 | 0.54 | I | 0.455 |
| Methenamine | 0 | 2688 | 36 | 2.944 | 3.892 | -2.52 | I | 0.035 |
| Methocarbamol | 0 | 2150 | 25 | 3.466 | 4.443 | -0.63 | I | 0.221 |
| Methoxsalen | 0 | 3516 | 52 | 3.784 | 5.476 | 0.55 | I | 0.385 |
| Methscopolamine | 0 | 5980 | 76 | 3.912 | 5.037 | 0.47 | I | 0.219 |
| Methylatropine | 0 | 4652 | 59 | 3.807 | 4.836 | 0.48 | I | 0.295 |
| Methyldopa | 0 | 2702 | 23 | 3.526 | 4.564 | -1.84 | I | 0.067 |
| Methyleneblue | 0 | 4188 | 54 | 3.97 | 5.778 | 0.13 | I | 0.252 |
| Methylphenidate | 0 | 2728 | 38 | 3.497 | 4.663 | -0.19 | I | 0.272 |
| Methylthiouracil | 0 | 1314 | 15 | 2.996 | 3.367 | -1.43 | I | 0.140 |
| Methysergidemaleate | 0 | 5958 | 88 | 4.111 | 5.73 | 2.21 | A | 0.619 |
| Metolazone | 0 | 5612 | 72 | 4.22 | 5.7 | 1.45 | I | 0.454 |
| Metronidazole | 0 | 1832 | 24 | 3.219 | 4.078 | -1.05 | I | 0.170 |
| Mianserin | 0 | 4394 | 67 | 3.912 | 5.557 | 1.52 | A | 0.560 |
| Miconazolenitrate | 0 | 4298 | 57 | 4.094 | 5.333 | 1.86 | A | 0.648 |
| Midodrine | 0 | 2600 | 31 | 3.555 | 4.762 | -0.7 | I | 0.189 |
| Minaprine | 0 | 3556 | 47 | 3.871 | 5.283 | 0.66 | I | 0.409 |
| Minocycline | 0 | 9136 | 126 | 4.419 | 5.971 | 2.58 | A | 0.481 |
| Molsidomine | 0 | 2464 | 35 | 3.466 | 4.248 | 0.44 | I | 0.432 |
| Monensin | 0 | 11610 | 149 | 4.431 | 5.268 | 2.06 | A | 0.212 |
| Montelukast | 0 | 8206 | 98 | 4.605 | 6.052 | 1.63 | A | 0.319 |
| Moxifloxacin | 0 | 7090 | 103 | 4.234 | 5.82 | 2.62 | A | 0.637 |
| Mupirocin | 0 | 5392 | 61 | 4.025 | 4.419 | 1.35 | I | 0.446 |
| Nadide | 0 | 9388 | 125 | 4.615 | 5.829 | 3.41 | A | 0.664 |
| Nafcillin | 0 | 7724 | 100 | 4.248 | 5.72 | 1.2 | I | 0.260 |
| Nafronyl | 0 | 4432 | 61 | 4.007 | 5.347 | 1.57 | A | 0.571 |
| Nalbuphine | 0 | 8914 | 124 | 4.127 | 5.753 | 1.29 | I | 0.215 |
| Nalidixicacid | 0 | 3508 | 46 | 3.761 | 5.318 | -0.12 | I | 0.241 |
| Naltrexone | 0 | 8548 | 122 | 4.143 | 5.808 | 1.76 | A | 0.327 |
| Natamycin | 0 | 8624 | 107 | 4.454 | 5.215 | 2.51 | A | 0.502 |
| Nateglinide | 0 | 3600 | 43 | 3.784 | 4.71 | 0.47 | I | 0.358 |
| Nefopam | 0 | 3338 | 49 | 3.784 | 5.283 | 0.83 | I | 0.465 |
| Neomycin | 0 | 9046 | 118 | 4.22 | 5.011 | 2.06 | A | 0.362 |
| Neostigmine | 0 | 2700 | 25 | 3.526 | 4.635 | -1.69 | I | 0.078 |
| Niacin | 0 | 1158 | 12 | 3.045 | 4.143 | -2.55 | I | 0.053 |
| Niacinamide | 0 | 1158 | 12 | 3.045 | 4.143 | -2.55 | I | 0.053 |
| Nicardipine | 0 | 6310 | 87 | 4.394 | 5.79 | 3.04 | A | 0.770 |
| Nicergoline | 20 | 7682 | 111 | 4.331 | 5.892 | 4.48 | A | 0.905 |
| Niclosamide | 0 | 3768 | 45 | 3.989 | 5.394 | 0.51 | I | 0.359 |
| Nicotineditartrate | 0 | 1942 | 27 | 3.219 | 4.369 | -1.36 | I | 0.127 |
| Nicotinylalcoholtartrate | 0 | 900 | 10 | 2.833 | 3.932 | -3.27 | I | 0.028 |
| Nifedipine | 0 | 4976 | 70 | 4.094 | 5.557 | 1.9 | A | 0.612 |
| Nilutamide | 0 | 5090 | 60 | 3.97 | 5.313 | -0.05 | I | 0.177 |
| Nimodipine | 0 | 5378 | 75 | 4.19 | 5.598 | 2.31 | A | 0.679 |
| Nisoldipine | 0 | 5310 | 74 | 4.159 | 5.591 | 2.13 | A | 0.643 |
| Nithiamide | 0 | 1624 | 20 | 3.296 | 3.989 | -0.55 | I | 0.264 |
| Nitrendipine | 0 | 4966 | 69 | 4.111 | 5.561 | 1.88 | A | 0.609 |
| Nitrofurantoin | 0 | 2704 | 36 | 3.689 | 4.625 | 0.85 | I | 0.516 |
| Nitrofurazone | 0 | 1756 | 21 | 3.434 | 4.248 | -0.26 | I | 0.316 |
| Nitromide | 0 | 2466 | 27 | 3.611 | 5.017 | -1.07 | I | 0.144 |
| Nizatidine | 0 | 2426 | 32 | 3.638 | 4.127 | 1.39 | I | 0.665 |
| Nomifensine | 0 | 3688 | 52 | 3.807 | 5.366 | 0.54 | I | 0.371 |
| Nonoxyno | 0 | 1930 | 25 | 3.401 | 4.5 | -0.7 | I | 0.221 |
| Norepinephrine | 0 | 1872 | 19 | 3.258 | 4.466 | -2.24 | I | 0.058 |
| Norethindrone | 0 | 6282 | 81 | 3.871 | 4.963 | 0.42 | I | 0.197 |
| Norethynodrel | 0 | 6282 | 81 | 3.892 | 5.03 | 0.43 | I | 0.199 |
| Norgestimate | 0 | 7132 | 100 | 4.043 | 5.231 | 1.94 | A | 0.467 |
| Norgestrel | 0 | 6504 | 87 | 3.892 | 5.017 | 0.84 | I | 0.259 |
| Noscapine | 0 | 7328 | 116 | 4.317 | 6.028 | 4.09 | A | 0.877 |
| Novobiocin | 0 | 9382 | 121 | 4.635 | 6.04 | 2.63 | A | 0.477 |
| Nystatin | 0 | 10550 | 130 | 4.691 | 5.338 | 3.08 | A | 0.505 |
| Octisalate | 0 | 2348 | 28 | 3.497 | 4.522 | -0.56 | I | 0.224 |
| Ofloxacin | 0 | 6330 | 90 | 4.143 | 5.817 | 1.79 | A | 0.489 |
| Omeprazole | 0 | 4688 | 63 | 4.127 | 5.645 | 1.57 | A | 0.553 |
| Orlistat | 0 | 4766 | 54 | 3.871 | 4.263 | 0.95 | I | 0.393 |
| Ornidazole | 0 | 2144 | 27 | 3.332 | 4.205 | -0.78 | I | 0.198 |
| Orphenadrinecitrate | 0 | 3070 | 41 | 3.761 | 5.118 | 0.41 | I | 0.381 |
| Oseltamivir | 0 | 3352 | 43 | 3.611 | 4.466 | 0.3 | I | 0.337 |
| Ouabain | 0 | 12364 | 165 | 4.407 | 5.493 | 2.21 | A | 0.201 |
| Oxantel | 0 | 2458 | 30 | 3.584 | 4.836 | -0.5 | I | 0.229 |
| Oxcarbazepine | 0 | 3938 | 59 | 3.932 | 5.565 | 1.44 | I | 0.574 |
| Oxibendazole | 0 | 2810 | 40 | 3.689 | 5.094 | 0.38 | I | 0.393 |
| Oxiconazolenitrate | 0 | 4368 | 58 | 4.159 | 5.434 | 2.09 | A | 0.695 |
| Oxolinicacid | 0 | 4238 | 60 | 3.892 | 5.472 | 0.89 | I | 0.415 |
| Oxybenzone | 0 | 2916 | 37 | 3.761 | 5.22 | -0.01 | I | 0.299 |
| Oxybutynin | 0 | 4338 | 53 | 3.892 | 4.97 | 0.59 | I | 0.338 |
| Oxymetazoline | 0 | 3872 | 46 | 3.807 | 5.094 | -0.18 | I | 0.213 |
| Oxyphencyclimine | 0 | 4842 | 60 | 3.912 | 5.011 | 0.6 | I | 0.307 |
| Oxyquinoline | 0 | 2032 | 26 | 3.401 | 4.949 | -1.53 | I | 0.107 |
| Oxytetracycline | 0 | 9968 | 138 | 4.431 | 6.038 | 2.52 | A | 0.407 |
| Pancuronium | 0 | 11112 | 150 | 4.357 | 5.416 | 2.48 | A | 0.321 |
| Pantoprazole | 0 | 4764 | 67 | 4.143 | 5.642 | 2.08 | A | 0.666 |
| Papaverine | 0 | 4786 | 66 | 4.127 | 5.796 | 1.53 | A | 0.535 |
| Parachlorophenol | 0 | 1032 | 10 | 2.944 | 4.078 | -3.09 | I | 0.032 |
| Paramethadione | 0 | 2322 | 23 | 3.091 | 3.401 | -1.81 | I | 0.077 |
| Pararosaniline | 0 | 4020 | 54 | 4.094 | 5.784 | 1.23 | I | 0.516 |
| Paromomycin | 0 | 9046 | 118 | 4.22 | 5.011 | 2.06 | A | 0.362 |
| Paroxetine | 0 | 4482 | 63 | 4.007 | 5.371 | 1.7 | A | 0.599 |
| Pefloxacine | 0 | 5140 | 69 | 4.025 | 5.553 | 1.01 | I | 0.382 |
| Penfluridol | 0 | 7338 | 87 | 4.419 | 5.802 | 1.12 | I | 0.266 |
| Penicillamine | 0 | 1408 | 6 | 2.708 | 0 | 1.11 | I | 0.668 |
| Penicilling | 0 | 5898 | 71 | 3.951 | 5.05 | 0.17 | I | 0.176 |
| Penicillinv | 0 | 5960 | 71 | 3.97 | 5.043 | 0.17 | I | 0.174 |
| Penteticacid | 0 | 3032 | 37 | 3.784 | 3.85 | 2.25 | A | 0.797 |
| Pentylenetetrazol | 0 | 1438 | 22 | 2.996 | 3.738 | -1.35 | I | 0.145 |
| Pergolidemesylate | 0 | 5018 | 79 | 3.892 | 5.505 | 1.88 | A | 0.603 |
| Perindoprilerbumine | 0 | 4522 | 64 | 3.784 | 4.575 | 1.73 | A | 0.603 |
| Perphenazine | 0 | 5208 | 74 | 4.078 | 5.638 | 1.75 | A | 0.558 |
| Phenacemide | 0 | 1562 | 19 | 3.332 | 4.234 | -0.76 | I | 0.229 |
| Phenacetin | 0 | 1634 | 20 | 3.296 | 4.466 | -1.39 | I | 0.134 |
| Phenelzine | 0 | 1048 | 13 | 2.944 | 4.06 | -2.68 | I | 0.048 |
| Phenindione | 0 | 3594 | 54 | 3.85 | 5.468 | 1.1 | I | 0.514 |
| Pheniramine | 0 | 2662 | 36 | 3.664 | 5.043 | 0.06 | I | 0.329 |
| Phenolphthalein | 0 | 4500 | 62 | 4.143 | 5.727 | 1.77 | A | 0.614 |
| Phenoxybenzamine | 0 | 2840 | 37 | 3.738 | 4.905 | 0.54 | I | 0.429 |
| Phentermine | 0 | 1602 | 16 | 3.135 | 4.143 | -2.33 | I | 0.058 |
| Phenyl | 0 | 2788 | 35 | 3.761 | 5.088 | 0.2 | I | 0.352 |
| Phenylephrine | 0 | 1628 | 19 | 3.178 | 4.331 | -2.02 | I | 0.076 |
| Phenylethylalcohol | 0 | 978 | 12 | 2.89 | 4.007 | -2.93 | I | 0.039 |
| Phenylmercuricacetate | 0 | 1234 | 14 | 3.135 | 4.159 | -1.89 | I | 0.095 |
| Phthalylsulfathiazole | 0 | 4998 | 63 | 4.304 | 5.591 | 2.15 | A | 0.668 |
| Physostigminesalicylate | 0 | 4844 | 70 | 3.829 | 5.308 | 0.93 | I | 0.384 |
| Phytonadione | 0 | 5152 | 64 | 4.174 | 5.421 | 1.46 | I | 0.491 |
| Pilocarpine | 0 | 2520 | 35 | 3.401 | 4.007 | 0.34 | I | 0.402 |
| Pimozide | 0 | 6560 | 94 | 4.382 | 5.823 | 3.37 | A | 0.812 |
| Pinacidil | 0 | 2636 | 26 | 3.611 | 4.691 | -0.99 | I | 0.148 |
| Pindolol | 0 | 2754 | 37 | 3.611 | 4.868 | -0.02 | I | 0.306 |
| Pioglitazone | 0 | 3990 | 53 | 4.043 | 5.273 | 1.71 | A | 0.635 |
| Piperazine | 0 | 516 | 6 | 1.946 | 1.946 | -5.2 | I | 0.005 |
| Piperidolate | 0 | 4020 | 57 | 3.932 | 5.263 | 1.52 | A | 0.588 |
| Piperine | 0 | 3374 | 46 | 3.829 | 5.106 | 0.93 | I | 0.488 |
| Pirenzepine | 0 | 5222 | 77 | 4.127 | 5.68 | 2.36 | A | 0.700 |
| Polymyxinb | 0 | 12090 | 156 | 4.956 | 5.714 | 4.58 | A | 0.744 |
| Potassiump-Aminobenzoate | 0 | 1416 | 14 | 3.178 | 4.344 | -2.3 | I | 0.062 |
| Pramoxine | 0 | 2604 | 34 | 3.555 | 4.615 | -0.05 | I | 0.309 |
| Pravastatin | 0 | 5138 | 67 | 3.97 | 4.883 | 1.54 | A | 0.513 |
| Praziquantel | 0 | 4992 | 70 | 3.912 | 5.22 | 1.31 | I | 0.465 |
| Prazosin | 0 | 5686 | 79 | 4.22 | 5.743 | 2.18 | A | 0.630 |
| Prilocaine | 0 | 2144 | 24 | 3.434 | 4.489 | -1.04 | I | 0.160 |
| Primaquine | 0 | 3104 | 43 | 3.714 | 5.333 | -0.05 | I | 0.279 |
| Probenecid | 0 | 3270 | 35 | 3.784 | 4.89 | -0.28 | I | 0.226 |
| Procainamide | 0 | 2190 | 24 | 3.466 | 4.511 | -0.97 | I | 0.167 |
| Prochlorperazine | 0 | 5004 | 70 | 4.043 | 5.624 | 1.41 | I | 0.490 |
| Procyclidine | 0 | 3850 | 50 | 3.664 | 4.736 | 0.13 | I | 0.271 |
| Proglumide | 0 | 3192 | 40 | 3.784 | 4.71 | 0.87 | I | 0.485 |
| Promethazine | 0 | 4088 | 56 | 3.871 | 5.517 | 0.43 | I | 0.320 |
| Propafenone | 0 | 3388 | 44 | 3.912 | 5.118 | 1.13 | I | 0.536 |
| Propylthiouracil | 0 | 1518 | 19 | 3.091 | 3.714 | -1.29 | I | 0.150 |
| Pyridoxine | 0 | 2032 | 23 | 3.296 | 4.522 | -1.87 | I | 0.079 |
| Pyrimethamine | 0 | 3152 | 42 | 3.784 | 5.361 | 0.11 | I | 0.309 |
| Pyrithione | 0 | 1096 | 10 | 2.833 | 3.664 | -3.21 | I | 0.029 |
| Quetiapine | 0 | 4836 | 72 | 4.094 | 5.697 | 2.22 | A | 0.692 |
| Quinacrine | 0 | 5144 | 71 | 4.143 | 5.852 | 1.5 | A | 0.502 |
| Quinidine | 0 | 5538 | 76 | 4.007 | 5.591 | 1 | I | 0.352 |
| Quipazine | 0 | 2988 | 41 | 3.638 | 5.22 | -0.37 | I | 0.224 |
| Racephedrine | 0 | 1668 | 18 | 3.135 | 4.22 | -2.32 | I | 0.057 |
| Ramipril | 0 | 5224 | 75 | 4.06 | 5.017 | 2.8 | A | 0.783 |
| Ranitidine | 0 | 2426 | 32 | 3.638 | 4.143 | 1.36 | I | 0.659 |
| Reserpine | 0 | 10050 | 150 | 4.605 | 6.12 | 4.95 | A | 0.884 |
| Resorcinol | 0 | 1040 | 11 | 2.944 | 3.97 | -2.79 | I | 0.043 |
| Retinol | 0 | 3226 | 34 | 3.761 | 4.625 | -0.02 | I | 0.277 |
| Ribavirin | 0 | 3164 | 43 | 3.526 | 4.407 | 0.24 | I | 0.337 |
| Riboflavin | 0 | 5806 | 76 | 4.159 | 5.765 | 1.11 | I | 0.361 |
| Rimantadine | 0 | 3870 | 48 | 3.219 | 4.205 | -2.06 | I | 0.040 |
| Risedronate | 0 | 3894 | 31 | 3.714 | 4.466 | -1.79 | I | 0.051 |
| Ritanserin | 0 | 6852 | 96 | 4.454 | 5.951 | 3.29 | A | 0.786 |
| Ritodrine | 0 | 3216 | 37 | 3.829 | 5.056 | 0.1 | I | 0.302 |
| Rizatriptan | 0 | 3396 | 50 | 3.85 | 5.215 | 1.38 | I | 0.597 |
| Rolipram | 0 | 3444 | 50 | 3.738 | 5.004 | 0.95 | I | 0.486 |
| Rolitetracycline | 0 | 10424 | 143 | 4.511 | 6.04 | 2.79 | A | 0.441 |
| Ronidazole | 0 | 2080 | 25 | 3.401 | 4.19 | -0.47 | I | 0.255 |
| Ropinirole | 0 | 2960 | 43 | 3.638 | 4.949 | 0.42 | I | 0.392 |
| Rosiglitazone | 0 | 3990 | 53 | 4.025 | 5.209 | 1.71 | A | 0.634 |
| Roxithromycin | 0 | 11724 | 139 | 4.554 | 5.231 | 1.29 | I | 0.108 |
| Rutoside | 0 | 9746 | 126 | 4.575 | 6.004 | 2.28 | A | 0.365 |
| Saccharin | 0 | 2746 | 35 | 3.638 | 5.056 | -0.43 | I | 0.227 |
| Salicin | 0 | 3726 | 46 | 3.638 | 4.754 | -0.36 | I | 0.190 |
| Salicylamide | 0 | 1496 | 16 | 3.178 | 4.263 | -2.05 | I | 0.077 |
| Salicylanilide | 0 | 2522 | 32 | 3.689 | 4.997 | 0.03 | I | 0.331 |
| Salsalate | 0 | 3252 | 41 | 3.871 | 5.187 | 0.61 | I | 0.418 |
| Scopolamine | 0 | 5262 | 70 | 3.85 | 4.97 | 0.81 | I | 0.327 |
| Selamectin | 0 | 12486 | 173 | 4.682 | 5.814 | 4.23 | A | 0.647 |
| Selegiline | 0 | 1744 | 20 | 3.296 | 4.277 | -1.29 | I | 0.143 |
| Sennosidea | 0 | 15458 | 222 | 5.03 | 6.777 | 5.53 | A | 0.740 |
| Sertraline | 0 | 4136 | 56 | 3.892 | 5.451 | 0.58 | I | 0.350 |
| Sibutramine | 0 | 4396 | 47 | 3.638 | 4.852 | -1.74 | I | 0.047 |
| Sildenafilcitrate | 0 | 7164 | 98 | 4.407 | 5.922 | 2.69 | A | 0.649 |
| Sodium Phenylacetate | 0 | 1172 | 14 | 3.091 | 4.143 | -2.02 | I | 0.086 |
| Sodium Salicylate | 0 | 1496 | 16 | 3.178 | 4.263 | -2.05 | I | 0.077 |
| Sodiumdehydrocholate | 0 | 7836 | 105 | 4.094 | 5.193 | 1.6 | A | 0.337 |
| Sodiumnitroprusside | 0 | 4812 | 15 | 3.526 | 0 | 0.65 | I | 0.321 |
| Sodiumoxybate | 0 | 420 | 3 | 2.197 | 1.386 | -2.89 | I | 0.047 |
| Solifenacinsuccinate | 0 | 6038 | 86 | 4.127 | 5.598 | 2.11 | A | 0.587 |
| Sorbitol | 0 | 1534 | 12 | 2.708 | 2.197 | -2.08 | I | 0.074 |
| Sparteinesulfate | 0 | 4116 | 60 | 3.401 | 4.443 | -0.18 | I | 0.202 |
| Spectinomycin | 0 | 5974 | 77 | 3.738 | 4.71 | 0.09 | I | 0.161 |
| Spiperone | 0 | 5820 | 77 | 4.174 | 5.416 | 1.91 | A | 0.556 |
| Spironolactone | 0 | 8514 | 117 | 4.159 | 5.347 | 2.03 | A | 0.392 |
| Strychnine | 0 | 8370 | 141 | 4.174 | 5.858 | 4.83 | A | 0.917 |
| Succinylsulfathiazole | 0 | 3700 | 44 | 4.06 | 5.075 | 1.5 | A | 0.605 |
| Sucralose | 0 | 5040 | 62 | 3.611 | 4.304 | -0.2 | I | 0.159 |
| Sulfacetamide | 0 | 2404 | 24 | 3.638 | 4.625 | -0.51 | I | 0.229 |
| Sulfachlorpyridazine | 0 | 3290 | 36 | 3.932 | 5.226 | 0.17 | I | 0.312 |
| Sulfameter | 0 | 3416 | 38 | 3.951 | 5.257 | 0.26 | I | 0.323 |
| Sulfamethazine | 0 | 3572 | 41 | 3.989 | 5.257 | 0.59 | I | 0.390 |
| Sulfamethizole | 0 | 3100 | 36 | 3.871 | 4.942 | 0.65 | I | 0.438 |
| Sulfamethoxypyridazine | 0 | 3416 | 38 | 3.951 | 5.257 | 0.26 | I | 0.323 |
| Sulfamonomethoxine | 0 | 3424 | 39 | 3.951 | 5.247 | 0.39 | I | 0.354 |
| Sulfanilate | 0 | 4380 | 43 | 4.19 | 5.303 | 0.43 | I | 0.300 |
| Sulfanitran | 0 | 4072 | 46 | 4.127 | 5.403 | 0.89 | I | 0.427 |
| Sulfapyridine | 0 | 3032 | 34 | 3.871 | 5.147 | 0.16 | I | 0.328 |
| Sulfaquinoxaline | 0 | 4218 | 51 | 4.127 | 5.649 | 0.86 | I | 0.410 |
| Sulfasalazine | 0 | 5108 | 61 | 4.357 | 5.781 | 1.67 | A | 0.545 |
| Sulfinpyrazone | 0 | 5528 | 82 | 4.277 | 5.707 | 3.33 | A | 0.849 |
| Sulfisoxazole | 0 | 3478 | 39 | 3.932 | 5.043 | 0.52 | I | 0.379 |
| Suloctidil | 0 | 2822 | 34 | 3.611 | 4.654 | -0.2 | I | 0.265 |
| Sulpiride | 0 | 4144 | 49 | 3.97 | 5.112 | 0.67 | I | 0.370 |
| Suramin | 0 | 19462 | 239 | 5.576 | 7.157 | 2.61 | A | 0.046 |
| Tacrine | 0 | 3306 | 46 | 3.689 | 5.416 | -0.34 | I | 0.214 |
| Tannic | 0 | 25620 | 323 | 5.762 | 7.234 | 2.84 | A | 0.010 |
| Taurine | 0 | 772 | 3 | 2.773 | 0 | 2.38 | A | 0.896 |
| Teicoplanin | 0 | 772 | 3 | 2.773 | 0 | 2.38 | A | 0.896 |
| Telithromycin | 0 | 12084 | 161 | 4.727 | 5.749 | 3.78 | A | 0.568 |
| Temefos | 0 | 4716 | 50 | 4.127 | 5.342 | 0.26 | I | 0.247 |
| Terazosin | 0 | 5686 | 79 | 4.143 | 5.649 | 1.86 | A | 0.553 |
| Terbinafine | 0 | 3522 | 42 | 3.932 | 5.338 | 0.33 | I | 0.334 |
| Terbutalinehemisulfate | 0 | 2580 | 26 | 3.497 | 4.543 | -1.33 | I | 0.111 |
| Terfenadine | 0 | 7028 | 85 | 4.344 | 5.697 | 1.18 | I | 0.296 |
| Tetracaine | 0 | 2290 | 27 | 3.526 | 4.635 | -0.59 | I | 0.221 |
| Tetrahydrozoline | 0 | 2894 | 43 | 3.555 | 4.868 | 0.17 | I | 0.339 |
| Tetramizole | 0 | 2600 | 40 | 3.497 | 4.779 | 0.14 | I | 0.351 |
| Thalidomide | 0 | 4198 | 61 | 3.871 | 5.252 | 1.35 | I | 0.533 |
| Theophylline | 0 | 2912 | 37 | 3.466 | 4.605 | -0.79 | I | 0.163 |
| Thiabendazole | 0 | 2600 | 40 | 3.664 | 5.112 | 0.62 | I | 0.465 |
| Thiamphenicol | 0 | 3594 | 35 | 3.871 | 4.852 | -0.32 | I | 0.204 |
| Thiamylal | 0 | 3450 | 41 | 3.466 | 4.143 | -0.53 | I | 0.178 |
| Thiothixene | 0 | 6426 | 80 | 4.331 | 5.9 | 1.27 | I | 0.354 |
| Thiram | 0 | 1342 | 8 | 2.944 | 2.639 | -1.52 | I | 0.128 |
| Thonzylamine | 0 | 3036 | 39 | 3.784 | 5.094 | 0.39 | I | 0.379 |
| Tiapride | 0 | 3564 | 38 | 3.892 | 5.043 | -0.04 | I | 0.254 |
| Ticarcillin | 0 | 6510 | 81 | 4.025 | 4.92 | 1 | I | 0.291 |
| Ticlopidine | 0 | 3118 | 43 | 3.714 | 4.868 | 0.72 | I | 0.454 |
| Tilmicosin | 0 | 11172 | 146 | 4.615 | 5.371 | 3.5 | A | 0.563 |
| Tilorone | 0 | 5228 | 74 | 4.159 | 5.838 | 1.87 | A | 0.587 |
| Timolol | 0 | 3284 | 40 | 3.611 | 4.248 | 0.39 | I | 0.363 |
| Tioconazole | 0 | 3834 | 53 | 3.989 | 5.017 | 2.12 | A | 0.733 |
| Tobramycin | 0 | 6700 | 84 | 3.932 | 4.673 | 0.87 | I | 0.254 |
| Tolazamide | 0 | 3380 | 40 | 3.871 | 4.836 | 0.82 | I | 0.460 |
| Tolazoline | 0 | 1618 | 23 | 3.296 | 4.357 | -0.76 | I | 0.226 |
| Tolbutamide | 0 | 2740 | 29 | 3.738 | 4.718 | -0.03 | I | 0.303 |
| Toltrazuril | 0 | 5862 | 72 | 4.248 | 5.576 | 1.33 | I | 0.409 |
| Topiramate | 0 | 5772 | 73 | 3.892 | 4.673 | 0.97 | I | 0.330 |
| Tramadol | 0 | 3696 | 45 | 3.611 | 4.852 | -0.78 | I | 0.136 |
| Tranexamicacid | 0 | 1542 | 16 | 2.833 | 3.045 | -2.22 | I | 0.065 |
| Tranylcypromine | 0 | 1890 | 19 | 3.135 | 4.277 | -2.72 | I | 0.037 |
| Trazodone | 0 | 4858 | 68 | 4.06 | 5.347 | 2.01 | A | 0.646 |
| Trichlormethiazide | 0 | 4818 | 54 | 4.078 | 5.425 | 0.15 | I | 0.223 |
| Triclosan | 0 | 2992 | 36 | 3.784 | 5.182 | -0.09 | I | 0.278 |
| Trientine | 0 | 514 | 6 | 2.197 | 1.792 | -3.36 | I | 0.029 |
| Trifluoperazine | 0 | 5898 | 76 | 4.143 | 5.677 | 0.98 | I | 0.324 |
| Trifluridine | 0 | 4170 | 51 | 3.689 | 4.585 | 0.04 | I | 0.237 |
| Trihexyphenidyl | 0 | 4032 | 51 | 3.689 | 4.787 | -0.03 | I | 0.231 |
| Trimethadione | 0 | 2124 | 20 | 3.045 | 3.219 | -1.8 | I | 0.081 |
| Trimethobenzamide | 0 | 4370 | 56 | 4.078 | 5.451 | 1.28 | I | 0.502 |
| Trimethoprim | 0 | 3732 | 49 | 3.912 | 5.375 | 0.69 | I | 0.401 |
| Trimetozine | 0 | 3522 | 47 | 3.714 | 5.088 | 0.08 | I | 0.281 |
| Trioxsalen | 0 | 3978 | 57 | 3.892 | 5.553 | 0.86 | I | 0.427 |
| Tripelennaminecitrate | 0 | 2652 | 35 | 3.689 | 4.963 | 0.24 | I | 0.370 |
| Trisodiumethylenediaminetetra | 0 | 2182 | 26 | 3.497 | 3.497 | 1.25 | I | 0.650 |
| Troleandomycin | 0 | 11476 | 146 | 4.605 | 5.318 | 2.92 | A | 0.398 |
| Tropicamide | 0 | 3228 | 43 | 3.807 | 5.043 | 0.78 | I | 0.461 |
| Tuaminoheptane | 0 | 490 | 4 | 2.079 | 1.386 | -3.63 | I | 0.022 |
| Tylosintartrate | 0 | 12322 | 158 | 4.682 | 5.464 | 3.1 | A | 0.383 |
| Tyrothricin | 0 | 14436 | 196 | 5.193 | 6.256 | 5.92 | A | 0.850 |
| Undecylenic | 0 | 654 | 8 | 2.398 | 2.079 | -2.6 | I | 0.058 |
| Urea | 0 | 162 | 0 | 1.792 | 0 | -2.95 | I | 0.048 |
| Valacyclovir | 0 | 3860 | 49 | 3.85 | 4.868 | 0.91 | I | 0.447 |
| Valsartan | 0 | 5500 | 78 | 4.277 | 5.724 | 2.81 | A | 0.771 |
| Vancomycin | 0 | 21584 | 296 | 5.434 | 6.872 | 5.78 | A | 0.381 |
| Vardenafil | 0 | 7290 | 100 | 4.419 | 5.883 | 2.86 | A | 0.677 |
| Vecuronium | 0 | 10434 | 145 | 4.317 | 5.384 | 2.95 | A | 0.481 |
| Venlafaxine | 0 | 3662 | 45 | 3.638 | 4.844 | -0.53 | I | 0.169 |
| Verapamil | 0 | 5820 | 70 | 4.205 | 5.501 | 1 | I | 0.334 |
| Vidarabine | 0 | 4110 | 60 | 3.761 | 5.165 | 0.85 | I | 0.415 |
| Warfarin | 0 | 4558 | 65 | 4.078 | 5.663 | 1.77 | A | 0.610 |
| Yohimbine | 0 | 6424 | 97 | 4.094 | 5.565 | 2.69 | A | 0.696 |
| Zidovudine | 0 | 3464 | 47 | 3.689 | 4.635 | 0.82 | I | 0.453 |
| Zolmitriptan | 0 | 3646 | 53 | 3.85 | 5.182 | 1.35 | I | 0.572 |

DF: discriminant function value for each compound

CLASS: classification of the model for ach compound

P.(Activ): probability of a compounds for being active

T(O…Br): topological distance between oxygen and bromide

SRW08: self-returning walk count of order 08

MPC04; molecular path count of order 04

piPC02: molecular multiple path count of order 02

piPC05: molecular multiple path count of order 05
