# Supplementary material for: Novel Cancer Chemotherapy Hits by Molecular Topology: Dual Akt and Beta-Catenin Inhibitors
Source: PLoS One. 2015 Apr 24;10(4):e0124244. doi: 10.1371/journal.pone.0124244 (PMC4409212; doi:10.1371/journal.pone.0124244)
Supplement: S4 Table — (DOCX) [file pone.0124244.s004.docx]

**S4 Table. Compounds used in the *test set* and corresponding values of the DF_2_ to Akt inhibitors.**

| **COMPOUNDS** | **T(O..Br)** | **SRW08** | **MPC04** | **piPC02** | **piPC05** | **DF** | **CLASS** | **P. (Activ.)** |
| --- | --- | --- | --- | --- | --- | --- | --- | --- |
| **ACTIVE GROUP** | | | | | | | | |
| Akt inhibitor IV [95] | 0 | 7498 | 116 | 4.575 | 6.28 | 4.93 | A | 0.940 |
| AZD5363 [96] | 0 | 6062 | 79 | 4.234 | 5.572 | 1.81 | A | 0.513 |
| GDC-0068 [97] | 0 | 6326 | 89 | 4.248 | 5.613 | 2.67 | A | 0.697 |
| API-59CJ-OMe [98] | 0 | 5442 | 81 | 4.174 | 6.08 | 2.08 | A | 0.623 |
| PHT-427 [99] | 0 | 3684 | 46 | 4.025 | 5.011 | 1.7 | A | 0.652 |
| Sunitinib [100] | 0 | 5406 | 77 | 4.234 | 5.67 | 2.68 | A | 0.753 |
| **INACTIVE GROUP** | | | | | | | | |
| Acepromazinemaleate | 0 | 4502 | 62 | 4.007 | 5.62 | 1.1 | I | 0.449 |
| Acetohexamide | 0 | 3806 | 43 | 3.951 | 4.963 | 0.66 | I | 0.391 |
| Acetohydroxamicacid | 0 | 272 | 0 | 1.946 | 0 | -2.2 | I | 0.092 |
| Adenine | 0 | 1834 | 25 | 3.296 | 4.654 | -1.43 | I | 0.124 |
| Adenosine | 0 | 4110 | 60 | 3.761 | 5.165 | 0.85 | I | 0.415 |
| Alpha-Tochopherol | 0 | 5540 | 64 | 4.06 | 5.242 | 0.28 | I | 0.209 |
| Alrestatin | 0 | 4422 | 63 | 3.951 | 5.69 | 0.93 | I | 0.412 |
| Amifostine | 0 | 1122 | 8 | 2.833 | 2.197 | -1.02 | I | 0.206 |
| Aminacrine | 0 | 3306 | 46 | 3.784 | 5.631 | -0.11 | I | 0.255 |
| Aminopentamide | 0 | 4294 | 54 | 3.892 | 5.323 | 0.21 | I | 0.262 |
| Ampicillin | 0 | 6276 | 74 | 3.989 | 5.1 | -0.03 | I | 0.136 |
| Antazoline | 0 | 3036 | 44 | 3.829 | 5.069 | 1.4 | I | 0.626 |
| Antipyrine | 0 | 2594 | 36 | 3.526 | 4.779 | -0.21 | I | 0.275 |
| Aspartame | 0 | 2838 | 33 | 3.714 | 4.595 | 0.38 | I | 0.390 |
| Astemizole | 0 | 6204 | 88 | 4.382 | 5.838 | 3.23 | A | 0.807 |
| Benzoicacid | 0 | 1158 | 12 | 3.045 | 4.143 | -2.55 | I | 0.053 |
| Biperiden | 0 | 4904 | 68 | 3.85 | 5.043 | 1.12 | I | 0.426 |
| Bismuthsubsalicylate | 0 | 0 | 23 | 2.89 | 4.29 | 0.05 | I | 0.513 |
| Bretyliumtosylate | 0 | 2114 | 20 | 3.296 | 4.382 | -2.21 | I | 0.056 |
| Butylparaben | 0 | 1760 | 20 | 3.332 | 4.477 | -1.43 | I | 0.125 |
| Camphor | 0 | 3540 | 36 | 3.178 | 3.761 | -2.54 | I | 0.027 |
| Captopril | 0 | 2208 | 27 | 3.178 | 3.526 | -0.71 | I | 0.206 |
| Carbadox | 0 | 3364 | 43 | 3.97 | 5.666 | 0.46 | I | 0.374 |
| Carbidopa | 0 | 2900 | 26 | 3.555 | 4.595 | -1.7 | I | 0.073 |
| Carvedilol | 0 | 5314 | 78 | 4.248 | 5.841 | 2.8 | A | 0.778 |
| Cefazolin | 0 | 6512 | 83 | 4.205 | 5.226 | 1.87 | A | 0.495 |
| Cefmetazole | 0 | 7256 | 86 | 4.159 | 5.215 | 0.53 | I | 0.170 |
| Cefoxitin | 0 | 6996 | 83 | 4.127 | 5.176 | 0.5 | I | 0.178 |
| Cefpodoxime | 0 | 6408 | 81 | 4.143 | 5.288 | 1.31 | I | 0.366 |
| Ceftazidime | 0 | 8420 | 98 | 4.477 | 5.663 | 1.06 | I | 0.200 |
| Cephapirin | 0 | 6060 | 72 | 4.127 | 5.288 | 0.67 | I | 0.252 |
| Cetylpyridinium | 0 | 1888 | 25 | 3.434 | 4.248 | 0.02 | I | 0.372 |
| Chlorhexidine | 0 | 4358 | 54 | 4.234 | 5.215 | 2.41 | A | 0.758 |
| Chloroquine | 0 | 3470 | 45 | 3.807 | 5.293 | 0.14 | I | 0.297 |
| Chlorothiazide | 0 | 4152 | 45 | 4.025 | 5.468 | -0.15 | I | 0.204 |
| Chloroxylenol | 0 | 1756 | 16 | 3.219 | 4.317 | -2.41 | I | 0.051 |
| Chlorpromazine | 0 | 4118 | 58 | 3.912 | 5.565 | 0.82 | I | 0.407 |
| Cholesterol | 0 | 7118 | 94 | 3.951 | 4.99 | 0.98 | I | 0.252 |
| Cinnarizine | 0 | 4662 | 67 | 4.159 | 5.565 | 2.51 | A | 0.761 |
| Cinoxacin | 0 | 4238 | 60 | 3.892 | 5.472 | 0.89 | I | 0.415 |
| Citicoline | 0 | 5996 | 67 | 4.143 | 4.796 | 1.06 | I | 0.335 |
| Clidinium | 0 | 6262 | 76 | 4.094 | 5.451 | 0.33 | I | 0.184 |
| Clindamycin | 0 | 5040 | 66 | 3.761 | 4.431 | 1.07 | I | 0.403 |
| Clorsulon | 0 | 4312 | 44 | 4.111 | 5.394 | 0.05 | I | 0.231 |
| Cloxacillin | 0 | 7744 | 100 | 4.277 | 5.565 | 1.61 | A | 0.345 |
| Colforsin | 0 | 9566 | 119 | 4.094 | 5.215 | 0.02 | I | 0.060 |
| Cycloheximide | 0 | 3666 | 46 | 3.611 | 4.143 | 0.63 | I | 0.393 |
| Cysteine | 0 | 640 | 2 | 2.303 | 0 | -0.43 | I | 0.350 |
| Danazol | 0 | 7802 | 102 | 4.094 | 5.403 | 0.9 | I | 0.203 |
| Danthron | 0 | 4416 | 62 | 3.97 | 5.727 | 0.86 | I | 0.396 |
| Decamethonium | 0 | 2010 | 14 | 3.135 | 2.639 | -0.84 | I | 0.193 |
| Dehydrocholicacid | 0 | 7836 | 105 | 4.094 | 5.193 | 1.6 | A | 0.337 |
| Desoxycorticosterone | 0 | 6714 | 89 | 3.932 | 5.043 | 0.89 | I | 0.258 |
| Dexchlorpheniramine | 0 | 2920 | 38 | 3.738 | 5.13 | 0.13 | I | 0.328 |
| Diazoxide | 0 | 3186 | 38 | 3.761 | 5.268 | -0.49 | I | 0.195 |
| Dicloxacillin | 0 | 8090 | 105 | 4.317 | 5.613 | 1.77 | A | 0.358 |
| Dienestrol | 0 | 3370 | 44 | 3.932 | 5.384 | 0.83 | I | 0.464 |
| Diltiazem | 0 | 5226 | 75 | 4.159 | 5.565 | 2.48 | A | 0.724 |
| Dimenhydrinate | 0 | 2732 | 37 | 3.689 | 5.043 | 0.21 | I | 0.359 |
| Dioxybenzone | 0 | 3254 | 41 | 3.829 | 5.293 | 0.17 | I | 0.314 |
| Diphenhydramine | 0 | 2732 | 37 | 3.689 | 5.043 | 0.21 | I | 0.359 |
| Diphenylpyraline | 0 | 3486 | 48 | 3.807 | 5.165 | 0.74 | I | 0.433 |
| Doxycycline | 0 | 9258 | 131 | 4.394 | 5.981 | 2.85 | A | 0.540 |
| Dyclonine | 0 | 2848 | 36 | 3.638 | 4.754 | 0.02 | I | 0.309 |
| Econazole | 0 | 3960 | 53 | 4.043 | 5.273 | 1.77 | A | 0.650 |
| Ergocalciferol | 0 | 5850 | 73 | 4.025 | 5.024 | 1.05 | I | 0.342 |
| Estriol | 0 | 5720 | 78 | 3.871 | 5.22 | 0.69 | I | 0.275 |
| Ethacrynicacid | 0 | 3134 | 35 | 3.761 | 4.963 | -0.28 | I | 0.234 |
| Ethopropazine | 0 | 4348 | 61 | 3.912 | 5.489 | 0.9 | I | 0.411 |
| Ethylparaben | 0 | 1542 | 16 | 3.219 | 4.394 | -2.11 | I | 0.072 |
| Famciclovir | 0 | 3464 | 46 | 3.871 | 5.075 | 1.07 | I | 0.515 |
| Fampridine | 0 | 1096 | 10 | 2.944 | 3.97 | -3.04 | I | 0.034 |
| Finasteride | 0 | 7416 | 95 | 4.025 | 5.056 | 0.87 | I | 0.217 |
| Flumazenil | 0 | 4516 | 67 | 4.007 | 5.572 | 1.84 | A | 0.629 |
| Foscarnet | 0 | 1008 | 0 | 2.708 | 0 | 1.09 | I | 0.689 |
| Gabapentin | 0 | 1960 | 21 | 2.944 | 3.178 | -1.9 | I | 0.078 |
| Gatifloxacin | 0 | 6376 | 89 | 4.159 | 5.762 | 1.76 | A | 0.477 |
| Glipizide | 0 | 5222 | 62 | 4.29 | 5.412 | 1.79 | A | 0.567 |
| Glucosamine | 0 | 2228 | 23 | 2.89 | 3.219 | -2.57 | I | 0.038 |
| Glycopyrrolate | 0 | 4838 | 61 | 3.85 | 4.883 | 0.57 | I | 0.303 |
| Guanabenzacetate | 0 | 1972 | 22 | 3.466 | 4.595 | -0.95 | I | 0.179 |
| Homosalate | 0 | 3588 | 43 | 3.689 | 4.682 | -0.06 | I | 0.250 |
| Hycanthone | 0 | 4906 | 69 | 4.06 | 5.72 | 1.41 | I | 0.497 |
| Indapamide | 0 | 5072 | 65 | 4.19 | 5.561 | 1.62 | A | 0.536 |
| Irbesartan | 0 | 6556 | 98 | 4.357 | 5.846 | 3.73 | A | 0.861 |
| Isoetharinemesylate | 0 | 2662 | 30 | 3.497 | 4.635 | -1.11 | I | 0.132 |
| Isoxsuprine | 0 | 3278 | 39 | 3.829 | 5.004 | 0.34 | I | 0.351 |
| Itraconazole | 0 | 10010 | 142 | 4.727 | 6.073 | 4.77 | A | 0.866 |
| Josamycin | 0 | 10284 | 137 | 4.575 | 5.288 | 3.93 | A | 0.721 |
| Lamotrigine | 0 | 3074 | 38 | 3.761 | 5.323 | -0.36 | I | 0.222 |
| Lansoprazole | 0 | 4714 | 59 | 4.127 | 5.537 | 1.16 | I | 0.447 |
| Leucovorin | 0 | 6096 | 80 | 4.317 | 5.525 | 2.48 | A | 0.670 |
| Levalbuterol | 0 | 2762 | 27 | 3.526 | 4.644 | -1.55 | I | 0.087 |
| Levothyroxine | 0 | 4434 | 52 | 4.078 | 5.416 | 0.66 | I | 0.348 |
| Lidocaine | 0 | 2474 | 29 | 3.526 | 4.595 | -0.62 | I | 0.208 |
| Liothyronine | 0 | 4096 | 48 | 4.025 | 5.371 | 0.53 | I | 0.341 |
| Lithiumcitrate | 0 | 1972 | 17 | 3.178 | 3.219 | -1.08 | I | 0.161 |
| Lomefloxacin | 0 | 5582 | 78 | 4.078 | 5.638 | 1.55 | A | 0.481 |
| Mecamylamine | 0 | 3706 | 39 | 3.135 | 3.664 | -2.56 | I | 0.026 |
| Medroxyprogesteroneacetate | 0 | 8282 | 109 | 4.111 | 5.273 | 1.23 | I | 0.235 |
| Mefloquine | 0 | 5884 | 71 | 4.078 | 5.684 | -0.1 | I | 0.141 |
| Mepivacaine | 0 | 3190 | 39 | 3.638 | 4.727 | -0.21 | I | 0.244 |
| Mestranol | 0 | 6408 | 83 | 3.97 | 5.342 | 0.41 | I | 0.190 |
| Methazolamid | 0 | 2456 | 26 | 3.611 | 4.431 | -0.18 | I | 0.290 |
| Methicillin | 0 | 6844 | 85 | 4.078 | 5.323 | 0.52 | I | 0.187 |
| Methimazole | 0 | 898 | 9 | 2.639 | 2.565 | -2.28 | I | 0.073 |
| Methoxamine | 0 | 2406 | 28 | 3.401 | 4.727 | -1.63 | I | 0.089 |
| Methylergonovine | 0 | 5628 | 83 | 4.078 | 5.656 | 2.11 | A | 0.617 |
| Metoclopramide | 0 | 3000 | 35 | 3.664 | 4.875 | -0.46 | I | 0.208 |
| Mexiletine | 0 | 1902 | 21 | 3.296 | 4.382 | -1.64 | I | 0.100 |
| Miglitol | 0 | 2448 | 29 | 2.996 | 3.466 | -1.95 | I | 0.065 |
| Minoxidil | 0 | 2728 | 33 | 3.584 | 4.875 | -0.69 | I | 0.184 |
| Modafinil | 0 | 3000 | 42 | 3.807 | 5.153 | 0.91 | I | 0.510 |
| Monobenzone | 0 | 2120 | 26 | 3.584 | 4.883 | -0.45 | I | 0.256 |
| Morantelcitrate | 0 | 2324 | 29 | 3.497 | 4.431 | -0.22 | I | 0.290 |
| Moxalactam | 0 | 8990 | 109 | 4.431 | 5.635 | 1.19 | I | 0.194 |
| Nadolol | 0 | 3970 | 45 | 3.761 | 4.97 | -0.59 | I | 0.149 |
| Naloxone | 0 | 7984 | 118 | 4.094 | 5.796 | 2.05 | A | 0.434 |
| Naphazoline | 0 | 2884 | 42 | 3.714 | 5.247 | 0.4 | I | 0.393 |
| Netilmicin | 0 | 6850 | 86 | 4.007 | 4.804 | 1.09 | I | 0.289 |
| Norfloxacin | 0 | 4882 | 67 | 3.989 | 5.537 | 1.06 | I | 0.410 |
| Nortriptyline | 0 | 3552 | 54 | 3.892 | 5.576 | 1.26 | I | 0.557 |
| Nylidrin | 0 | 3278 | 39 | 3.829 | 5.004 | 0.34 | I | 0.351 |
| Olmesartan | 0 | 6608 | 95 | 4.419 | 5.953 | 3.42 | A | 0.817 |
| Oxacillin | 0 | 7406 | 96 | 4.234 | 5.493 | 1.59 | A | 0.363 |
| Oxethazaine | 0 | 5874 | 65 | 4.22 | 5.142 | 0.92 | I | 0.312 |
| Oxfendazole | 0 | 3948 | 55 | 4.043 | 5.553 | 1.59 | A | 0.609 |
| Oxidopamine | 0 | 1904 | 21 | 3.296 | 4.554 | -1.94 | I | 0.076 |
| Pargyline | 0 | 1368 | 17 | 3.178 | 4.174 | -1.51 | I | 0.130 |
| Pentoxifylline | 0 | 3858 | 52 | 3.761 | 4.89 | 0.73 | I | 0.403 |
| Perhexiline | 0 | 3228 | 46 | 3.367 | 4.078 | 0.09 | I | 0.301 |
| Phenazopyridine | 0 | 2458 | 30 | 3.714 | 5.142 | -0.21 | I | 0.284 |
| Phenformin | 0 | 1694 | 20 | 3.401 | 4.174 | -0.35 | I | 0.300 |
| Phenylpropanolamine | 0 | 1542 | 16 | 3.091 | 4.174 | -2.54 | I | 0.048 |
| Phenytoin | 0 | 4178 | 60 | 3.892 | 5.416 | 1.11 | I | 0.473 |
| Pipamperone | 0 | 5090 | 61 | 3.97 | 4.963 | 0.68 | I | 0.310 |
| Piperacillin | 0 | 8834 | 109 | 4.369 | 5.442 | 1.45 | I | 0.246 |
| Piracetam | 0 | 1304 | 16 | 2.89 | 3.045 | -1.38 | I | 0.146 |
| Pirenperone | 0 | 5732 | 75 | 4.234 | 5.602 | 1.87 | A | 0.552 |
| Pizotyline | 0 | 4312 | 67 | 3.97 | 5.602 | 1.97 | A | 0.671 |
| Pralidoxime | 0 | 1308 | 15 | 3.135 | 4.344 | -2.22 | I | 0.069 |
| Pregabalin | 0 | 1060 | 11 | 2.708 | 2.398 | -1.61 | I | 0.128 |
| Primidone | 0 | 3538 | 43 | 3.584 | 4.762 | -0.75 | I | 0.144 |
| Probucol | 0 | 8224 | 87 | 4.394 | 5.765 | -0.74 | I | 0.042 |
| Procaine | 0 | 2190 | 24 | 3.466 | 4.511 | -0.97 | I | 0.167 |
| Propantheline | 0 | 5730 | 70 | 4.111 | 5.617 | 0.4 | I | 0.220 |
| Propofol | 0 | 2226 | 25 | 3.332 | 4.533 | -1.78 | I | 0.081 |
| Propranolol | 0 | 2952 | 38 | 3.689 | 5.182 | -0.33 | I | 0.234 |
| Proscillaridin | 0 | 10714 | 137 | 4.382 | 5.565 | 1.39 | I | 0.152 |
| Protryptyline | 0 | 3552 | 54 | 3.871 | 5.591 | 1.1 | I | 0.518 |
| Pyrantel | 0 | 2010 | 26 | 3.401 | 4.304 | -0.38 | I | 0.275 |
| Pyrazinamide | 0 | 1158 | 12 | 3.045 | 4.143 | -2.55 | I | 0.053 |
| Pyridostigmine | 0 | 1806 | 19 | 3.332 | 4.369 | -1.48 | I | 0.119 |
| Pyrilamine | 0 | 3036 | 39 | 3.784 | 5.094 | 0.39 | I | 0.379 |
| Quinapril | 0 | 5540 | 73 | 4.22 | 5.425 | 2.2 | A | 0.643 |
| Quinine | 0 | 5538 | 76 | 4.007 | 5.545 | 1.08 | I | 0.370 |
| Rabeprazole | 0 | 4246 | 59 | 4.078 | 5.533 | 1.8 | A | 0.637 |
| Raloxifene | 0 | 6568 | 95 | 4.443 | 6.08 | 3.43 | A | 0.821 |
| Ranolazine | 0 | 5058 | 65 | 4.159 | 5.298 | 1.9 | A | 0.606 |
| Retinylpalmitate | 0 | 4588 | 52 | 4.174 | 5.017 | 1.64 | A | 0.575 |
| Rifampin | 0 | 12558 | 167 | 4.868 | 6.395 | 3.43 | A | 0.447 |
| Roxarsone | 0 | 2656 | 25 | 3.584 | 4.812 | -1.54 | I | 0.090 |
| Sarafloxacin | 0 | 6050 | 84 | 4.263 | 5.924 | 2.1 | A | 0.585 |
| Simvastatin | 0 | 6092 | 75 | 4.043 | 4.99 | 1.01 | I | 0.318 |
| Sisomicin | 0 | 6638 | 81 | 3.97 | 4.736 | 0.71 | I | 0.229 |
| Sulconazolenitrate | 0 | 3960 | 53 | 4.043 | 5.273 | 1.77 | A | 0.650 |
| Sulfabenzamide | 0 | 3416 | 38 | 3.97 | 5.209 | 0.46 | I | 0.369 |
| Sulfadiazine | 0 | 3032 | 34 | 3.871 | 5.147 | 0.16 | I | 0.328 |
| Sulfadimethoxine | 0 | 3824 | 45 | 4.025 | 5.357 | 0.69 | I | 0.396 |
| Sulfadoxine | 0 | 3968 | 47 | 4.025 | 5.38 | 0.64 | I | 0.374 |
| Sulfamerazine | 0 | 3298 | 37 | 3.932 | 5.204 | 0.33 | I | 0.347 |
| Sulfamethoxazole | 0 | 3100 | 36 | 3.871 | 4.956 | 0.63 | I | 0.432 |
| Sulfathiazole | 0 | 2850 | 33 | 3.807 | 4.86 | 0.48 | I | 0.414 |
| Tacrolimus | 0 | 10976 | 142 | 4.625 | 5.485 | 3.21 | A | 0.506 |
| Tetracycline | 0 | 9518 | 128 | 4.407 | 5.986 | 1.99 | A | 0.315 |
| Tetroquinone | 0 | 2496 | 24 | 3.434 | 4.443 | -1.66 | I | 0.084 |
| Thiamine | 0 | 3054 | 40 | 3.761 | 4.956 | 0.58 | I | 0.424 |
| Thimerosal | 0 | 1778 | 22 | 3.296 | 4.477 | -1.42 | I | 0.126 |
| Thiopental | 0 | 3364 | 38 | 3.434 | 4.127 | -0.94 | I | 0.128 |
| Thioridazine | 0 | 5132 | 72 | 4.043 | 5.649 | 1.39 | I | 0.474 |
| Tinidazole | 0 | 2692 | 28 | 3.664 | 4.248 | 0.27 | I | 0.373 |
| Tolnaftate | 0 | 4020 | 52 | 4.043 | 5.545 | 1.05 | I | 0.470 |
| Tranilast | 0 | 3838 | 49 | 4.043 | 5.451 | 1.16 | I | 0.512 |
| Triacetin | 0 | 1456 | 15 | 3.178 | 3.135 | -0.18 | I | 0.354 |
| Triamterene | 0 | 3946 | 54 | 3.989 | 5.72 | 0.83 | I | 0.422 |
| Trichlorfon | 0 | 2314 | 14 | 3.045 | 1.946 | -0.83 | I | 0.182 |
| Trilostane | 0 | 8424 | 106 | 4.007 | 5.242 | -0.07 | I | 0.075 |
| Trimipramine | 0 | 3996 | 59 | 3.912 | 5.425 | 1.44 | I | 0.570 |
| Triprolidine | 0 | 3366 | 47 | 3.892 | 5.333 | 1.09 | I | 0.528 |
| Tryptophan | 0 | 2592 | 34 | 3.584 | 4.883 | -0.3 | I | 0.259 |
| Tubocurarine | 0 | 10674 | 149 | 4.754 | 6.422 | 3.98 | A | 0.706 |
| Tyloxapol | 0 | 2842 | 27 | 3.466 | 4.5 | -1.84 | I | 0.065 |
| Ursodiol | 0 | 7402 | 99 | 3.951 | 4.934 | 1.19 | I | 0.278 |
| Valproate | 0 | 930 | 9 | 2.565 | 2.197 | -2.18 | I | 0.080 |
| Vesamicol | 0 | 3426 | 46 | 3.584 | 4.673 | 0.04 | I | 0.277 |
| Vigabatrin | 0 | 724 | 5 | 2.565 | 2.197 | -2.31 | I | 0.074 |
| Xylazine | 0 | 2468 | 31 | 3.526 | 4.736 | -0.57 | I | 0.216 |
| Xylometazoline | 0 | 3454 | 40 | 3.738 | 4.99 | -0.42 | I | 0.194 |
| Zoxazolamine | 0 | 2020 | 28 | 3.401 | 4.844 | -1.06 | I | 0.162 |

DF: discriminant function value for each compound

CLASS: classification of the model for ach compound

P.(Activ): probability of a compounds for being active

T(O…Br): topological distance between oxygen and bromide

SRW08: self-returning walk count of order 08

MPC04: molecular path count of order 04

piPC02; molecular multiple path count of order 02

piPC05: molecular multiple path count of order 05
