# Supplementary material for: Novel Cancer Chemotherapy Hits by Molecular Topology: Dual Akt and Beta-Catenin Inhibitors
Source: PLoS One. 2015 Apr 24;10(4):e0124244. doi: 10.1371/journal.pone.0124244 (PMC4409212; doi:10.1371/journal.pone.0124244)
Supplement: S5 Table — (DOCX) [file pone.0124244.s005.docx]

**S5 Table. Compounds used in the *training set* and corresponding values of the DF_3_ to β-catenin natural inhibitors.**

| **COMPOUNDS** | **Dz** | **S2K** | | **PCR** | | **X2sol** | | **JGI4** | | **DF** | | **CLASS** | | | **P. (Activ.)** |
| --- | --- | --- | --- | --- | --- | --- | --- | --- | --- | --- | --- | --- | --- | --- | --- |
| **ACTIVE GROUP** | | | | | | | | | | | | | | | |
| 10-Hydroxycampothecin [102] | 57 | 4.087 | | 1.441 | | 11.966 | | 0.055 | | 1.18 | | A | | 0.235 | |
| Agelastatine [103] | 44.75 | 2.881 | | 1.157 | | 10.396 | | 0.054 | | 0.58 | | A | | 0.359 | |
| Artenimol [104] | 45 | 2.168 | | 1 | | 9.702 | | 0.064 | | -1.67 | | I | | 0.842 | |
| Auraptene [105] | 47 | 4.938 | | 1.44 | | 9.478 | | 0.038 | | 1.02 | | A | | 0.265 | |
| Bergapten [106] | 36 | 2.65 | | 1.367 | | 7.005 | | 0.053 | | -0.27 | | I | | 0.566 | |
| Calphostin [107] | 130 | 11.693 | | 1.594 | | 25.567 | | 0.047 | | 1.48 | | A | | 0.187 | |
| Camptothecin [108] | 57 | 4.087 | | 1.441 | | 11.966 | | 0.055 | | 1.18 | | A | | 0.235 | |
| Cardamonin [109] | 44 | 4.855 | | 1.511 | | 8.358 | | 0.044 | | 1.30 | | A | | 0.215 | |
| Curcumin [110] | 60 | 7.293 | | 1.596 | | 11.25 | | 0.048 | | 2.90 | | A | | 0.052 | |
| Deguelin [111] | 64 | 4.179 | | 1.414 | | 13.478 | | 0.06 | | 1.00 | | A | | 0.269 | |
| Diallyltrisulfide [112] | 18 | 6.169 | | 1.144 | | 4.845 | | 0.016 | | 3.68 | | A | | 0.025 | |
| EGCG [113] | 81 | 6.466 | | 1.386 | | 16.573 | | 0.067 | | 1.87 | | A | | 0.134 | |
| Ellagic acid [114] | 52 | 3.689 | | 1.36 | | 10.38 | | 0.072 | | 1.11 | | A | | 0.249 | |
| Emodin [115] | 45 | 3.194 | | 1.392 | | 9.308 | | 0.064 | | 1.33 | | A | | 0.210 | |
| Esculetin [116] | 30 | 2.418 | | 1.336 | | 5.864 | | 0.085 | | 2.52 | | A | | 0.075 | |
| Gallic acid [117] | 29 | 2.522 | | 1.238 | | 5.289 | | 0.084 | | 1.44 | | A | | 0.192 | |
| Gossypol [118] | 84 | 5.767 | | 1.426 | | 17.005 | | 0.058 | | 0.00 | | A | | 0.500 | |
| Hecogenin [119] | 66 | 3.598 | | 1.008 | | 15.099 | | 0.066 | | 0.07 | | A | | 0.484 | |
| Honokiol [120] | 42 | 4.242 | | 1.459 | | 8.208 | | 0.045 | | 0.94 | | A | | 0.281 | |
| Indole-3-Carbinol [121] | 23.5 | 1.945 | | 1.337 | | 4.454 | | 0.029 | | -2.02 | | I | | 0.884 | |
| Indomethacin [122] | 54.83 | 5.152 | | 1.37 | | 11.432 | | 0.05 | | 1.64 | | A | | 0.162 | |
| Isoflavone [123] | 36 | 3.488 | | 1.502 | | 7.207 | | 0.041 | | 0.93 | | A | | 0.283 | |
| Isoliquiritigenin [124] | 42 | 4.757 | | 1.489 | | 8.24 | | 0.046 | | 1.86 | | A | | 0.134 | |
| Juglone [125] | 29 | 2.324 | | 1.387 | | 5.681 | | 0.056 | | 0.54 | | A | | 0.368 | |
| Kirenol [126] | 52 | 3.181 | | 1.054 | | 11.229 | | 0.054 | | -1.44 | | I | | 0.809 | |
| Murrayafolinea [127] | 33.5 | 2.121 | | 1.419 | | 7.017 | | 0.048 | | 0.12 | | A | | 0.469 | |
| Naringin [128] | 96 | 9.358 | | 1.242 | | 18.449 | | 0.049 | | -0.41 | | I | | 0.601 | |
| PFK118-310 [129] | 32 | 1.83 | | 1.31 | | 6.167 | | 0.076 | | 0.59 | | A | | 0.357 | |
| Protocatechualdehyde [130] | 23 | 2.171 | | 1.301 | | 4.042 | | 0.089 | | 2.34 | | A | | 0.088 | |
| Quercetin [131] | 51 | 4.453 | | 1.386 | | 10.037 | | 0.061 | | 1.16 | | A | | 0.240 | |
| Resveratrol [132] | 37 | 4.107 | | 1.459 | | 7.487 | | 0.055 | | 2.54 | | A | | 0.073 | |
| Rotenone [111] | 64 | 4.36 | | 1.319 | | 13.046 | | 0.057 | | -0.32 | | I | | 0.581 | |
| Silibinin [133] | 80 | 7.409 | | 1.371 | | 15.625 | | 0.053 | | 0.64 | | A | | 0.345 | |
| Sitosterol [134] | 61 | 3.657 | | 1.067 | | 13.732 | | 0.05 | | -0.87 | | I | | 0.706 | |
| Sulforaphane [135] | 21.5 | 5.219 | | 1.226 | | 4.688 | | 0.027 | | 2.21 | | A | | 0.099 | |
| Tanshinone [136] | 45 | 2.849 | | 1.415 | | 9.569 | | 0.057 | | 0.93 | | A | | 0.283 | |
| Theophylline [137] | 30 | 1.52 | | 1.195 | | 5.695 | | 0.074 | | -0.57 | | I | | 0.638 | |
| Toxoflavin [138] | 32.5 | 1.871 | | 1.31 | | 6.167 | | 0.076 | | 0.42 | | A | | 0.396 | |
| Uvaol [139] | 66 | 2.864 | | 1.059 | | 15.546 | | 0.067 | | 0.33 | | A | | 0.418 | |
| Vincristine [140] | 132 | 9.653 | | 1.254 | | 27.249 | | 0.047 | | -1.14 | | I | | 0.758 | |
| **INACTIVE GROUP** | | | | | | | | | | | | | | | |
| 1r,9s-Hydrastine | 62.5 | | 4.572 | | 1.33 | | 12.414 | | 0.046 | | -1.27 | | I | 0.782 | |
| 4-O-Methylphloracetophenone | 30 | | 2.155 | | 1.261 | | 5.522 | | 0.047 | | -1.85 | | I | 0.864 | |
| Abscisic Acid (Cis,Trans; +/-) | 42 | | 2.551 | | 1.283 | | 9.021 | | 0.052 | | -0.25 | | I | 0.563 | |
| Acetosyringone | 32 | | 2.241 | | 1.23 | | 5.671 | | 0.064 | | -1.19 | | I | 0.767 | |
| Ajmaline | 51 | | 2.581 | | 1.135 | | 11.358 | | 0.054 | | -1.00 | | I | 0.732 | |
| Albizziine | 24.5 | | 3.142 | | 1.09 | | 4.192 | | 0.053 | | -1.10 | | I | 0.750 | |
| Anisodamine | 48.5 | | 4.523 | | 1.157 | | 9.517 | | 0.037 | | -1.88 | | I | 0.868 | |
| Apiole | 36 | | 2.666 | | 1.241 | | 6.284 | | 0.048 | | -2.61 | | I | 0.931 | |
| Asarinin (-) | 58 | | 4.886 | | 1.289 | | 11.859 | | 0.031 | | -1.34 | | I | 0.793 | |
| Asarylaldehyde | 32 | | 2.627 | | 1.278 | | 5.145 | | 0.06 | | -1.62 | | I | 0.835 | |
| Asiatic acid | 75 | | 3.78 | | 1.065 | | 16.787 | | 0.066 | | -0.45 | | I | 0.611 | |
| Azadirachtin | 118 | | 6.688 | | 1.065 | | 24.273 | | 0.056 | | -3.96 | | I | 0.981 | |
| Beta-Escin | 182 | | 14.11 | | 1.06 | | 36.355 | | 0.059 | | -2.63 | | I | 0.933 | |
| Biochanin A | 47 | | 4.147 | | 1.43 | | 9.181 | | 0.048 | | 0.34 | | A | 0.415 | |
| Capsaicin | 47.5 | | 6.303 | | 1.291 | | 8.893 | | 0.041 | | 0.77 | | A | 0.317 | |
| Chlorogenic acid | 59 | | 6.136 | | 1.261 | | 11.365 | | 0.065 | | 1.43 | | A | 0.194 | |
| Cinchonidine | 46 | | 3.862 | | 1.352 | | 9.563 | | 0.043 | | 0.18 | | A | 0.457 | |
| Citropten | 34 | | 2.583 | | 1.337 | | 6.256 | | 0.056 | | -0.66 | | I | 0.660 | |
| Colchiceine | 62.5 | | 5.214 | | 1.359 | | 11.948 | | 0.048 | | -0.94 | | I | 0.720 | |
| Colchicine | 64.5 | | 5.299 | | 1.347 | | 12.139 | | 0.046 | | -1.62 | | I | 0.835 | |
| Colforsin | 65 | | 2.786 | | 1.038 | | 14.35 | | 0.072 | | -1.02 | | I | 0.735 | |
| Crustecdysone | 75 | | 4.2 | | 1.068 | | 17.219 | | 0.067 | | 0.85 | | A | 0.301 | |
| Cryptotanshinone | 47 | | 2.573 | | 1.244 | | 10.484 | | 0.058 | | 0.27 | | A | 0.435 | |
| D,L-Threo-3-Hydroxyaspartic acid | 25.5 | | 2.675 | | 1.084 | | 4.206 | | 0.04 | | -3.12 | | I | 0.958 | |
| Deltaline | 71.5 | | 3.294 | | 1 | | 15.392 | | 0.065 | | -2.29 | | I | 0.908 | |
| Dihydrocelastryl Diacetate | 84 | | 4.375 | | 1.237 | | 19.238 | | 0.069 | | 1.72 | | A | 0.152 | |
| Dihydromyristicin | 31 | | 2.389 | | 1.216 | | 5.649 | | 0.045 | | -2.23 | | I | 0.903 | |
| Diprotin A | 53.5 | | 5.048 | | 1.04 | | 9.869 | | 0.042 | | -3.15 | | I | 0.959 | |
| Eugenol.Mol | 26 | | 2.252 | | 1.31 | | 4.613 | | 0.052 | | -0.81 | | I | 0.691 | |
| Eupatorin | 57 | | 4.7 | | 1.318 | | 10.6 | | 0.054 | | -1.18 | | I | 0.765 | |
| Eupatoriochromene | 35 | | 1.853 | | 1.31 | | 7.784 | | 0.072 | | 1.66 | | A | 0.160 | |
| Evernic acid | 55 | | 4.704 | | 1.312 | | 10.604 | | 0.04 | | -1.47 | | I | 0.813 | |
| Frequentin | 40 | | 5.046 | | 1.251 | | 6.966 | | 0.051 | | -0.11 | | I | 0.527 | |
| Gambogic acid amide | 100 | | 6.08 | | 1.279 | | 22.267 | | 0.058 | | 1.22 | | A | 0.228 | |
| Griseofulvin | 54.33 | | 3.722 | | 1.266 | | 10.694 | | 0.063 | | -0.64 | | I | 0.655 | |
| Hydroquinidine | 51 | | 4.138 | | 1.305 | | 10.366 | | 0.046 | | -0.38 | | I | 0.593 | |
| Hydroxyprogesterone | 51 | | 2.73 | | 1.089 | | 11.603 | | 0.057 | | -0.47 | | I | 0.617 | |
| Ichthynone | 67 | | 4.607 | | 1.425 | | 13.821 | | 0.057 | | 0.61 | | A | 0.352 | |
| Isoginkgetin | 94 | | 8.626 | | 1.498 | | 18.912 | | 0.044 | | 1.54 | | A | 0.177 | |
| Kainic acid | 34.5 | | 2.692 | | 1.077 | | 6.686 | | 0.056 | | -1.66 | | I | 0.841 | |
| Khellin | 43 | | 2.745 | | 1.373 | | 8.198 | | 0.056 | | -0.92 | | I | 0.715 | |
| Kinetin | 35.5 | | 3.373 | | 1.398 | | 6.774 | | 0.026 | | -1.54 | | I | 0.824 | |
| Lappaconitine | 93 | | 5.796 | | 1.13 | | 19.358 | | 0.059 | | -1.72 | | I | 0.849 | |
| Ligustilide | 30 | | 2.633 | | 1.265 | | 5.727 | | 0.025 | | -2.68 | | I | 0.936 | |
| Madecassic acid | 78 | | 3.97 | | 1.063 | | 17.354 | | 0.066 | | -0.59 | | I | 0.645 | |
| Norstictic Acid | 63 | | 4.505 | | 1.341 | | 12.343 | | 0.052 | | -1.14 | | I | 0.757 | |
| Ouabain | 94 | | 6.754 | | 1.044 | | 19.458 | | 0.058 | | -1.48 | | I | 0.814 | |
| Pachyrrhizin | 56 | | 4.525 | | 1.506 | | 11.31 | | 0.051 | | 1.13 | | A | 0.244 | |
| Pachyrrhizone | 61 | | 4.548 | | 1.39 | | 12.346 | | 0.054 | | 0.24 | | A | 0.441 | |
| Paeonol | 27 | | 1.965 | | 1.288 | | 4.972 | | 0.051 | | -1.20 | | I | 0.769 | |
| Physcion | 47 | | 3.267 | | 1.368 | | 9.477 | | 0.064 | | 0.68 | | A | 0.336 | |
| Pimpinellin | 41 | | 2.926 | | 1.328 | | 7.649 | | 0.053 | | -1.27 | | I | 0.780 | |
| Piplartine | 51.5 | | 5.268 | | 1.37 | | 9.226 | | 0.044 | | -0.88 | | I | 0.707 | |
| Plectocomine Methyl Ether | 32 | | 2.301 | | 1.25 | | 6.455 | | 0.034 | | -2.11 | | I | 0.892 | |
| Protoporphyrin Ix | 90 | | 7.197 | | 1.49 | | 18.817 | | 0.03 | | 0.25 | | A | 0.440 | |
| Pseudo-Anisatin | 48 | | 2.125 | | 1.031 | | 10.403 | | 0.064 | | -1.67 | | I | 0.841 | |
| Pteryxin | 63 | | 4.426 | | 1.289 | | 12.948 | | 0.056 | | -0.24 | | I | 0.561 | |
| Pyridoxine | 27.5 | | 2.343 | | 1.296 | | 4.654 | | 0.065 | | -0.32 | | I | 0.580 | |
| Rutin.Mol | 102 | | 9.734 | | 1.311 | | 19.538 | | 0.055 | | 0.16 | | A | 0.461 | |
| Safrole.Mol | 26 | | 2.112 | | 1.282 | | 4.919 | | 0.041 | | -1.52 | | I | 0.821 | |
| Salicin | 47 | | 5.145 | | 1.197 | | 8.19 | | 0.053 | | -1.15 | | I | 0.761 | |
| Salsolidine | 32.5 | | 2.088 | | 1.254 | | 6.14 | | 0.064 | | -0.67 | | I | 0.662 | |
| Salsoline | 30.5 | | 2.012 | | 1.253 | | 5.949 | | 0.069 | | 0.18 | | A | 0.457 | |
| Salvinorin A | 70 | | 4.893 | | 1.093 | | 14.333 | | 0.054 | | -1.80 | | I | 0.859 | |
| Salvinorin B | 63 | | 4.266 | | 1.098 | | 12.92 | | 0.057 | | -1.59 | | I | 0.830 | |
| Strychnine | 53 | | 3.302 | | 1.189 | | 12.052 | | 0.041 | | -0.58 | | I | 0.641 | |
| Tetrahydropalmatine | 56.5 | | 4.183 | | 1.311 | | 10.946 | | 0.055 | | -0.97 | | I | 0.725 | |
| Theobromine | 30 | | 1.52 | | 1.198 | | 5.845 | | 0.065 | | -1.02 | | I | 0.735 | |
| Tryptamine | 41 | | 3.128 | | 1.461 | | 8.516 | | 0.048 | | 0.82 | | A | 0.307 | |
| Umbelliferone | 27 | | 2.222 | | 1.334 | | 5.357 | | 0.075 | | 1.93 | | A | 0.127 | |
| Vincamine | 56 | | 3.606 | | 1.195 | | 11.842 | | 0.051 | | -1.01 | | I | 0.733 | |

DF: discriminant function value for each compound

CLASS: classification of the model for ach compound

P.(Activ): probability of a compounds for being active

Dz: Pogliani index; S2K, 2-path Kier alpha-modified shape index

PCR: ratio of multiple path count over path count

X2sol: solvation connectivity index chi-2

JGI4: mean topological charge index of order 4.
