# Supplementary material for: Novel Cancer Chemotherapy Hits by Molecular Topology: Dual Akt and Beta-Catenin Inhibitors
Source: PLoS One. 2015 Apr 24;10(4):e0124244. doi: 10.1371/journal.pone.0124244 (PMC4409212; doi:10.1371/journal.pone.0124244)
Supplement: S6 Table — (DOCX) [file pone.0124244.s006.docx]

**S6 Table. Compounds used in the *test set* and corresponding values of the DF_3_ to**

**β-catenin natural inhibitors.**

| **COMPOUNDS** | **Dz** | **S2K** | **PCR** | **X2sol** | **JGI4** | **DF** | **CLASS** | **P. (Activ.)** |
| --- | --- | --- | --- | --- | --- | --- | --- | --- |
| **ACTIVE GROUP** | | | | | | | | |
| 4-Methylesculetin [116] | 32 | 2.212 | 1.312 | 6.404 | 0.078 | 1.60 | A | 0.831 |
| Apigenin [141] | 45 | 4.064 | 1.402 | 9.12 | 0.049 | 0.91 | A | 0.712 |
| Aspirin [142] | 30 | 2.922 | 1.337 | 5.582 | 0.044 | -0.61 | I | 0.351 |
| Caffeic acid [143] | 30 | 3.329 | 1.348 | 5.605 | 0.069 | 1.96 | A | 0.877 |
| Celastrol [144] | 70 | 3.318 | 1.23 | 16.392 | 0.074 | 2.16 | A | 0.896 |
| Celecoxib [145] | 60 | 5.249 | 1.391 | 12.289 | 0.051 | 1.16 | A | 0.761 |
| CGP049090 [146] | 88 | 6.304 | 1.419 | 17.945 | 0.05 | -0.22 | I | 0.445 |
| Diosgenin [147] | 63 | 3.407 | 1.06 | 14.64 | 0.062 | 0.38 | A | 0.593 |
| FH535 [148] | 49.7 | 5.299 | 1.4 | 12.31 | 0.061 | 6.52 | N.C. | 0.999 |
| Fisetin [149] | 48 | 4.258 | 1.395 | 9.488 | 0.063 | 1.53 | A | 0.821 |
| Hydnocarpin [150] | 77 | 7.217 | 1.414 | 15.216 | 0.049 | 0.98 | A | 0.726 |
| ICG-001 [152] | 88 | 9.825 | 1.316 | 18.034 | 0.042 | 2.78 | A | 0.941 |
| ICRT-14 [152] | 57.5 | 5.392 | 1.447 | 12.553 | 0.036 | 1.98 | A | 0.878 |
| IWP-2 [153] | 66 | 7.287 | 1.429 | 15.656 | 0.041 | 5.93 | N.C. | 0.997 |
| JW55 [154] | 70 | 7.955 | 1.306 | 13.603 | 0.029 | -0.07 | I | 0.482 |
| Monocrotaline [153] | 52.5 | 3.209 | 1.082 | 10.936 | 0.037 | -3.28 | I | 0.036 |
| Paclitaxel [155] | 138.5 | 11.711 | 1.22 | 28.58 | 0.051 | 0.78 | A | 0.686 |
| PKF118-744 [156] | 53 | 4.131 | 1.396 | 10.493 | 0.059 | 0.57 | A | 0.639 |
| PKF222-815 [157] | 140 | 11.329 | 1.584 | 27.896 | 0.046 | 0.44 | A | 0.608 |
| PNU-74654 [158] | 52 | 6.127 | 1.577 | 10.141 | 0.028 | 1.42 | A | 0.805 |
| Shikonin [159] | 47 | 3.899 | 1.38 | 9.371 | 0.058 | 0.85 | A | 0.700 |
| Sulindac [160] | 54.5 | 5.073 | 1.443 | 11.547 | 0.045 | 1.94 | A | 0.874 |
| XAV-939 [161] | 48.5 | 4.663 | 1.305 | 8.435 | 0.043 | -2.09 | I | 0.110 |
| ZTM000990 [162] | 115.5 | 8.266 | 1.308 | 22.333 | 0.056 | -2.70 | I | 0.063 |
| **INACTIVE GROUP** | | | | | | | | |
| 1-Methylxanthine | 28 | 1.641 | 1.214 | 5.286 | 0.073 | -0.20 | I | 0.450 |
| Anethole | 23 | 2.097 | 1.318 | 4.084 | 0.048 | -0.84 | I | 0.301 |
| Bicuculline (+) | 60.5 | 4.507 | 1.305 | 12.529 | 0.042 | -0.78 | I | 0.313 |
| Chrysanthemic acid, ethylester | 30 | 1.046 | 1.103 | 6.349 | 0.06 | -1.74 | I | 0.149 |
| Crassin acetate | 59 | 4.82 | 1.185 | 12.316 | 0.043 | -0.79 | I | 0.312 |
| Dihydrojasmonic acid, methyl ester | 35 | 3.949 | 1.053 | 6.223 | 0.036 | -2.90 | I | 0.052 |
| Inosine | 45 | 3.743 | 1.237 | 8.263 | 0.044 | -2.30 | I | 0.091 |
| Melatonin | 37 | 3.278 | 1.292 | 7.173 | 0.036 | -1.50 | I | 0.182 |
| Penicillic acid | 28 | 1.252 | 1.159 | 5.311 | 0.06 | -1.99 | I | 0.120 |
| Pomiferin | 68 | 4.925 | 1.397 | 14.598 | 0.055 | 1.49 | A | 0.816 |
| Pyrromycin | 95.5 | 7.077 | 1.272 | 19.183 | 0.054 | -1.09 | I | 0.252 |
| Quassin | 62 | 3.026 | 1.128 | 12.892 | 0.067 | -1.68 | I | 0.157 |
| Reserpine | 98 | 8.103 | 1.252 | 18.855 | 0.047 | -2.17 | I | 0.102 |
| Rutilantinone | 71 | 5.264 | 1.362 | 14.082 | 0.058 | -0.21 | I | 0.447 |
| Strophanthidin | 64 | 4.269 | 1.072 | 13.867 | 0.056 | -0.70 | I | 0.330 |
| Trigonelline | 22.5 | 1.742 | 1.29 | 4.276 | 0.066 | 0.53 | A | 0.630 |
| Vindoline | 73 | 4.36 | 1.233 | 14.92 | 0.055 | -1.80 | I | 0.142 |

DF: discriminant function value for each compound

CLASS: classification of the model for ach compound

P.(Activ): probability of a compounds for being active

Dz: Pogliani index; S2K, 2-path Kier alpha-modified shape index

PCR: ratio of multiple path count over path count

X2sol: solvation connectivity index chi-2

JGI4: mean topological charge index of order 4.
