# Supplementary material for: Novel Cancer Chemotherapy Hits by Molecular Topology: Dual Akt and Beta-Catenin Inhibitors
Source: PLoS One. 2015 Apr 24;10(4):e0124244. doi: 10.1371/journal.pone.0124244 (PMC4409212; doi:10.1371/journal.pone.0124244)
Supplement: S7 Table — (DOCX) [file pone.0124244.s007.docx]

**S7 Table. Compounds used in the *training set* and corresponding values of the DF_4_ to β-catenin** **inhibitors.**

| **COMPOUNDS** | **SCBO** | **NN** | **ZM1** | **GGI4** | **DF** | **CLASS** | **P. (ACTIV.)** |
| --- | --- | --- | --- | --- | --- | --- | --- |
| **ACTIVE GROUP** | | | | | | | |
| 10-Hydroxycampothecin [102] | 39 | 2 | 154 | 2.53 | 0.33 | A | 0.571 |
| 2,4-Diaminoquinazoline [163] | 18 | 4 | 62 | 0.71 | -0.81 | I | 0.305 |
| 4-Methylesculetin [116] | 20 | 0 | 74 | 1.25 | 0.60 | A | 0.640 |
| Agelastatine [103] | 27 | 4 | 122 | 1.73 | -1.83 | I | 0.134 |
| Apigenin [141] | 30 | 0 | 108 | 1.43 | 0.80 | A | 0.683 |
| Artenimol [104] | 23 | 0 | 122 | 2.45 | -0.85 | I | 0.291 |
| Aspirin [142] | 18 | 0 | 60 | 0.66 | 0.46 | A | 0.609 |
| Auraptene [164] | 30 | 0 | 106 | 0.87 | 0.29 | A | 0.566 |
| Azaserine [165] | 15 | 3 | 48 | 0.32 | -0.68 | I | 0.335 |
| Bergapten [166] | 24 | 0 | 88 | 1.21 | 0.52 | A | 0.621 |
| Caffeic acid [143] | 18 | 0 | 60 | 0.69 | 0.49 | A | 0.618 |
| Calphostin [167] | 83 | 0 | 318 | 6.36 | 4.23 | A | 0.984 |
| Camptothecin [108] | 39 | 2 | 154 | 2.53 | 0.33 | A | 0.571 |
| Celecoxib [145] | 38 | 3 | 142 | 1.92 | -0.03 | I | 0.483 |
| CGP049090 [146] | 55 | 0 | 226 | 5.27 | 2.75 | A | 0.936 |
| Curcumin [168] | 38 | 0 | 130 | 1.55 | 1.31 | A | 0.783 |
| Dexamethasone [169] | 35 | 0 | 168 | 3.82 | 0.34 | A | 0.572 |
| Diallyltrisulfide [112] | 10 | 0 | 30 | 0.08 | 0.00 | A | 0.499 |
| Ellagic acid [170] | 33 | 0 | 130 | 3.18 | 1.94 | A | 0.870 |
| Fenoprofen [171] | 26 | 0 | 88 | 0.65 | 0.38 | A | 0.588 |
| FH535 [148] | 32 | 2 | 114 | 1.96 | 0.89 | A | 0.703 |
| Fisetin [149] | 31 | 0 | 114 | 1.9 | 1.15 | A | 0.754 |
| Gallic acid [117] | 16 | 0 | 58 | 0.92 | 0.40 | A | 0.596 |
| Gossypol [172] | 53 | 0 | 210 | 5.48 | 3.69 | A | 0.974 |
| Hecogenin [119] | 37 | 0 | 192 | 4.11 | -0.63 | I | 0.336 |
| Honokiol [173] | 29 | 0 | 98 | 1.23 | 1.06 | A | 0.737 |
| Hydnocarpin [150] | 49 | 0 | 188 | 2.88 | 1.30 | A | 0.778 |
| Ibuprofen [174] | 19 | 0 | 70 | 0.74 | 0.05 | A | 0.509 |
| ICG-001 [152] | 60 | 4 | 222 | 2.81 | 0.30 | A | 0.560 |
| ICRT-14 [152] | 41 | 3 | 146 | 1.53 | -0.02 | I | 0.487 |
| Indole-3-Carbinol [175] | 16 | 1 | 56 | 0.26 | -0.51 | I | 0.373 |
| Indomethacin [122] | 36 | 1 | 132 | 2.05 | 0.94 | A | 0.713 |
| Isoflavone [123] | 27 | 0 | 90 | 0.9 | 0.77 | A | 0.678 |
| Isoliquiritigenin [124] | 28 | 0 | 94 | 0.92 | 0.74 | A | 0.672 |
| IWP-2 [153] | 46 | 4 | 170 | 1.92 | -0.39 | I | 0.394 |
| Juglone [176] | 20 | 0 | 68 | 0.9 | 0.64 | A | 0.651 |
| JW55 [154] | 45 | 2 | 166 | 1.32 | -0.46 | I | 0.377 |
| Kirenol [126] | 27 | 0 | 136 | 2.55 | -0.77 | I | 0.308 |
| LGK-974 [146] | 46 | 6 | 156 | 1.21 | -0.75 | I | 0.313 |
| Melphalan [177] | 23 | 2 | 86 | 0.82 | -0.62 | I | 0.345 |
| Mesalamine [178] | 15 | 1 | 52 | 0.65 | -0.01 | I | 0.495 |
| Monocrotaline [179] | 28 | 1 | 130 | 1.58 | -1.49 | I | 0.179 |
| Murrayafoline A [127] | 24 | 1 | 88 | 1.06 | 0.05 | A | 0.508 |
| Mycophenolatemofetil [180] | 39 | 1 | 156 | 2.1 | -0.03 | I | 0.482 |
| Nabumetone [181] | 24 | 0 | 84 | 1.07 | 0.65 | A | 0.652 |
| Naproxen [182] | 24 | 0 | 86 | 1.11 | 0.55 | A | 0.628 |
| Naringin [128] | 52 | 0 | 224 | 3.46 | 0.04 | A | 0.495 |
| Nocodazole [183] | 31 | 3 | 110 | 0.81 | -0.69 | I | 0.328 |
| NSC668036 [184] | 36 | 2 | 150 | 2 | -0.75 | I | 0.312 |
| Paclitaxel [155] | 84 | 1 | 346 | 7.65 | 3.58 | A | 0.970 |
| PFK118-310 [129] | 20 | 4 | 74 | 1.29 | -0.53 | I | 0.368 |
| Phenylbutazone [185] | 33 | 2 | 118 | 1.81 | 0.66 | A | 0.653 |
| Piroxicam [186] | 35 | 3 | 124 | 2.25 | 0.94 | A | 0.713 |
| PKF118-744 [156] | 35 | 0 | 130 | 2.76 | 1.95 | A | 0.872 |
| PKF222-815 [157] | 87 | 0 | 344 | 7.04 | 4.08 | A | 0.982 |
| PNU-74654 [158] | 36 | 2 | 120 | 0.78 | 0.08 | A | 0.513 |
| Protocatechualdehyde [130] | 14 | 0 | 46 | 0.53 | 0.34 | A | 0.582 |
| Pyrvinium [187] | 43 | 3 | 156 | 1.98 | 0.26 | A | 0.556 |
| Quercetin [188] | 32 | 0 | 120 | 2.13 | 1.22 | A | 0.766 |
| Rotenone [189] | 41 | 0 | 166 | 3.06 | 1.13 | A | 0.747 |
| Shikonin [159] | 29 | 0 | 108 | 2.04 | 1.25 | A | 0.772 |
| Sitosterol [134] | 34 | 0 | 168 | 2.65 | -1.28 | I | 0.210 |
| Sorafenib [190] | 45 | 4 | 164 | 1.72 | -0.43 | I | 0.384 |
| Sulforaphane [135] | 12 | 1 | 36 | 0.16 | -0.14 | I | 0.463 |
| Sulindac [160] | 37 | 0 | 132 | 1.68 | 1.07 | A | 0.737 |
| Tanshinone [191] | 33 | 0 | 122 | 2.21 | 1.41 | A | 0.799 |
| Theophylline [192] | 18 | 4 | 70 | 1.19 | -0.85 | I | 0.297 |
| Topotecan [189] | 44 | 3 | 180 | 3.32 | 0.26 | A | 0.553 |
| Toxoflavin [193] | 20 | 5 | 74 | 1.29 | -0.82 | I | 0.303 |
| Uvaol [139] | 37 | 0 | 196 | 5.06 | 0.18 | A | 0.532 |
| Vincristine [140] | 80 | 4 | 354 | 8.24 | 1.77 | A | 0.842 |
| XAV-939 [161] | 29 | 2 | 114 | 1.21 | -0.75 | I | 0.315 |
| ZTM000990 [162] | 67 | 1 | 286 | 7.29 | 3.34 | A | 0.963 |
| **INACTIVE GROUP** | | | | | | | |
| Acarbose | 48 | 1 | 234 | 4.5 | -0.81 | I | 0.294 |
| Acebutolol | 29 | 2 | 110 | 1.29 | -0.35 | I | 0.406 |
| Aceclidine | 14 | 1 | 62 | 0.46 | -1.23 | I | 0.223 |
| Acedapsone | 34 | 2 | 116 | 1.24 | 0.41 | A | 0.595 |
| Acepromazinemaleate | 32 | 2 | 120 | 1.47 | -0.13 | I | 0.459 |
| Acetaminophen | 15 | 1 | 50 | 0.5 | -0.03 | I | 0.489 |
| Acetanilide | 14 | 1 | 44 | 0.31 | -0.06 | I | 0.483 |
| Acetarsol | 20 | 1 | 74 | 1.05 | 0.08 | A | 0.515 |
| Acetohexamide | 30 | 2 | 110 | 0.97 | -0.47 | I | 0.379 |
| Acetohydroxamic acid | 5 | 1 | 16 | 0 | -0.60 | I | 0.353 |
| Acetriazoic acid | 21 | 1 | 78 | 1.4 | 0.43 | A | 0.601 |
| Acetylcholine | 10 | 1 | 42 | 0.24 | -1.00 | I | 0.266 |
| Acetylcysteine | 11 | 1 | 40 | 0.48 | -0.32 | I | 0.418 |
| Acyclovir | 21 | 5 | 80 | 0.93 | -1.43 | I | 0.190 |
| Adenosine | 25 | 5 | 104 | 1.19 | -1.91 | I | 0.126 |
| Adenosinephosphate | 30 | 5 | 126 | 1.51 | -1.92 | I | 0.125 |
| Adiphenine | 31 | 1 | 108 | 0.78 | 0.01 | A | 0.495 |
| Aklomide | 18 | 2 | 62 | 0.73 | -0.19 | I | 0.448 |
| Alaproclate | 21 | 1 | 82 | 1.06 | -0.26 | I | 0.431 |
| Albuterol | 20 | 1 | 82 | 1.01 | -0.57 | I | 0.357 |
| Alclometazonedipropionate | 35 | 0 | 166 | 3.79 | 0.45 | A | 0.599 |
| Alfluzosin | 36 | 5 | 142 | 2.06 | -0.96 | I | 0.270 |
| Algesteroneacetophenide | 44 | 0 | 202 | 4.04 | 0.33 | A | 0.569 |
| Aliskirenhemifumarate | 44 | 3 | 184 | 2.37 | -1.13 | I | 0.235 |
| Allopurinol | 15 | 4 | 52 | 0.33 | -1.25 | I | 0.220 |
| Alpha-tochopherol | 35 | 0 | 154 | 2.11 | -0.59 | I | 0.346 |
| Alprenolol | 22 | 1 | 80 | 0.7 | -0.27 | I | 0.428 |
| Alrestatin | 29 | 1 | 104 | 1.85 | 1.04 | A | 0.734 |
| Althiazide | 31 | 3 | 116 | 1.8 | 0.00 | A | 0.493 |
| Altrenogest | 31 | 0 | 134 | 1.98 | -0.26 | I | 0.425 |
| Alverinecitrate | 28 | 1 | 96 | 0.22 | -0.50 | I | 0.371 |
| Amantadine | 13 | 1 | 68 | 0.33 | -2.09 | I | 0.109 |
| Amcinonide | 46 | 0 | 220 | 5 | 0.60 | A | 0.631 |
| Amikacin | 43 | 5 | 206 | 3.55 | -2.26 | I | 0.090 |
| Amiloride | 20 | 7 | 72 | 1 | -1.58 | I | 0.168 |
| Aminacrine | 24 | 2 | 82 | 1.08 | 0.23 | A | 0.553 |
| Aminocaproic acid | 9 | 1 | 32 | 0.16 | -0.60 | I | 0.352 |
| Aminohippuric acid | 19 | 2 | 64 | 0.54 | -0.31 | I | 0.419 |
| Aminolevulinic acid | 10 | 1 | 34 | 0.32 | -0.31 | I | 0.421 |
| Aminopentamide | 30 | 2 | 110 | 1.46 | 0.09 | A | 0.517 |
| Aminosalicylate | 15 | 1 | 52 | 0.49 | -0.19 | I | 0.449 |
| Amiodarone | 41 | 1 | 156 | 1.99 | 0.35 | A | 0.577 |
| Amiprilose | 27 | 1 | 144 | 2.39 | -1.85 | I | 0.131 |
| Amitraz | 31 | 3 | 108 | 1.15 | -0.15 | I | 0.455 |
| Amitriptyline | 30 | 1 | 108 | 0.99 | -0.01 | I | 0.491 |
| Amlodipine | 36 | 2 | 136 | 2.42 | 0.78 | A | 0.678 |
| Amodiaquine | 35 | 3 | 128 | 1.48 | -0.25 | I | 0.430 |
| Amoxicillin | 33 | 3 | 138 | 2.31 | -0.54 | I | 0.359 |
| Amphotericinb | 76 | 1 | 318 | 3.71 | -0.92 | I | 0.268 |
| Ampicillin | 32 | 3 | 132 | 2.12 | -0.57 | I | 0.354 |
| Ampyzinesulfate | 12 | 3 | 40 | 0.19 | -0.99 | I | 0.270 |
| Amsacrine | 43 | 3 | 152 | 1.97 | 0.55 | A | 0.626 |
| Anagrelide | 23 | 3 | 90 | 1.12 | -0.86 | I | 0.292 |
| Aniracetam | 22 | 1 | 80 | 0.74 | -0.23 | I | 0.439 |
| Antazoline | 29 | 3 | 100 | 0.54 | -0.77 | I | 0.312 |
| Anthralin | 26 | 0 | 94 | 1.46 | 0.87 | A | 0.699 |
| Antipyrine | 20 | 2 | 72 | 0.6 | -0.59 | I | 0.353 |
| Apomorphine | 29 | 1 | 116 | 2.02 | 0.33 | A | 0.575 |
| Apramycin | 40 | 5 | 200 | 3.59 | -2.52 | I | 0.071 |
| Aripiprazole | 40 | 3 | 156 | 1.54 | -1.01 | I | 0.259 |
| Armodafinil | 28 | 1 | 92 | 0.7 | 0.35 | A | 0.582 |
| Arsanilic acid | 15 | 1 | 54 | 0.42 | -0.42 | I | 0.392 |
| Ascorbic acid | 14 | 0 | 58 | 0.65 | -0.42 | I | 0.392 |
| Ascorbylpalmitate | 32 | 0 | 128 | 0.89 | -0.82 | I | 0.298 |
| Astemizole | 48 | 4 | 180 | 1.69 | -0.90 | I | 0.280 |
| Atenolol | 23 | 2 | 86 | 0.94 | -0.48 | I | 0.377 |
| Atovaquone | 38 | 0 | 142 | 2.05 | 1.00 | A | 0.724 |
| Atropineoxide | 29 | 1 | 118 | 1.01 | -0.99 | I | 0.265 |
| Atropinesulfate | 27 | 1 | 110 | 0.82 | -1.12 | I | 0.241 |
| Avobenzone | 32 | 0 | 116 | 1.08 | 0.30 | A | 0.568 |
| Azacitidine | 21 | 4 | 88 | 1.19 | -1.44 | I | 0.188 |
| Azaperone | 33 | 3 | 120 | 0.6 | -1.18 | I | 0.229 |
| Azathioprine | 28 | 7 | 102 | 0.98 | -1.83 | I | 0.136 |
| Azelaic acid | 14 | 0 | 50 | 0.24 | -0.30 | I | 0.423 |
| Azelastine | 38 | 3 | 144 | 1.77 | -0.36 | I | 0.402 |
| Azithromycin | 55 | 2 | 274 | 5.12 | -1.61 | I | 0.156 |
| Aztreonam | 37 | 5 | 148 | 1.61 | -1.68 | I | 0.152 |
| Bacampicillin | 41 | 3 | 168 | 2.44 | -0.61 | I | 0.342 |
| Bacitracin | 123 | 17 | 484 | 7.2 | -2.06 | I | 0.102 |
| Beclomethasone | 45 | 0 | 204 | 4.86 | 1.38 | A | 0.790 |
| Bekanamycin | 35 | 5 | 174 | 3.11 | -2.40 | I | 0.080 |
| Benazepril | 42 | 2 | 154 | 1.84 | 0.28 | A | 0.561 |
| Bendroflumethiazide | 39 | 3 | 150 | 2.79 | 0.63 | A | 0.645 |
| Benurestat | 20 | 2 | 68 | 0.54 | -0.36 | I | 0.408 |
| Benzbromarone | 32 | 0 | 118 | 1.79 | 0.97 | A | 0.720 |
| Benzethonium | 37 | 1 | 150 | 1.33 | -0.98 | I | 0.265 |
| Benzocaine | 16 | 1 | 54 | 0.38 | -0.22 | I | 0.443 |
| Benzoic acid | 13 | 0 | 40 | 0.19 | 0.14 | A | 0.533 |
| Benzoylpas | 28 | 1 | 94 | 0.88 | 0.41 | A | 0.596 |
| Benzoylperoxide | 27 | 0 | 86 | 0.38 | 0.47 | A | 0.610 |
| Benzthiazide | 39 | 3 | 140 | 1.79 | 0.22 | A | 0.547 |
| Benzydamine | 32 | 3 | 116 | 0.66 | -1.07 | I | 0.251 |
| Benzylbenzoat | 24 | 0 | 76 | 0.34 | 0.41 | A | 0.597 |
| Bepheniumhydroxynapthoate | 26 | 1 | 92 | 0.62 | -0.25 | I | 0.433 |
| Betahistine | 13 | 2 | 42 | 0.15 | -0.64 | I | 0.344 |
| Betaine | 8 | 1 | 34 | 0 | -1.19 | I | 0.232 |
| Betamethasone | 35 | 0 | 168 | 3.82 | 0.34 | A | 0.572 |
| Betamethasone-1,7,2,1-dipropionate | 45 | 0 | 204 | 4.86 | 1.38 | A | 0.790 |
| Betamethasoneacetate | 39 | 0 | 182 | 4.06 | 0.58 | A | 0.629 |
| Beta-Propiolactone | 6 | 0 | 22 | 0 | -0.51 | I | 0.375 |
| Bethanechol | 11 | 2 | 48 | 0.24 | -1.49 | I | 0.182 |
| Bezafibrate | 34 | 1 | 124 | 1.16 | 0.01 | A | 0.495 |
| Bifonazole | 38 | 2 | 126 | 0.59 | -0.07 | I | 0.474 |
| Biotin | 19 | 2 | 80 | 0.53 | -1.52 | I | 0.176 |
| Bisacodyl | 40 | 1 | 136 | 1.36 | 0.86 | A | 0.695 |
| Bismuthsubsalicylate | 13 | 7 | 0 | 1 | 2.04 | A | 0.885 |
| Bithionate | 26 | 0 | 98 | 1.37 | 0.46 | A | 0.607 |
| Bleomycin | 118 | 17 | 488 | 7.15 | -3.68 | I | 0.022 |
| Bretyliumtosylate | 16 | 1 | 62 | 0.82 | -0.31 | I | 0.420 |
| Bromhexine | 22 | 2 | 90 | 1 | -0.97 | I | 0.270 |
| Bromindione | 28 | 0 | 98 | 1.13 | 0.69 | A | 0.660 |
| Bromperidol | 35 | 1 | 134 | 1.02 | -0.65 | I | 0.335 |
| Brompheniraminemaleate | 26 | 2 | 92 | 0.57 | -0.60 | I | 0.350 |
| Brucine | 28 | 0 | 98 | 1.13 | 0.69 | A | 0.660 |
| Budesonide | 39 | 0 | 184 | 4.02 | 0.39 | A | 0.583 |
| Bupropion | 20 | 1 | 78 | 0.86 | -0.44 | I | 0.386 |
| Buspirone | 36 | 5 | 148 | 1.28 | -2.31 | I | 0.087 |
| Busulfan | 17 | 0 | 62 | 0.4 | -0.25 | I | 0.434 |
| Butacaine | 26 | 2 | 96 | 0.62 | -0.84 | I | 0.296 |
| Butylparaben | 18 | 0 | 62 | 0.46 | 0.08 | A | 0.515 |
| Caffeine | 19 | 4 | 76 | 1.39 | -0.81 | I | 0.304 |
| Camphor | 13 | 0 | 66 | 0.11 | -1.90 | I | 0.128 |
| Candesartan | 64 | 6 | 238 | 2.21 | -1.17 | I | 0.227 |
| Canrenoic acid | 33 | 0 | 152 | 2.64 | -0.34 | I | 0.406 |
| Canrenone | 33 | 0 | 154 | 2.53 | -0.62 | I | 0.340 |
| Capobenic acid | 28 | 1 | 104 | 1.44 | 0.31 | A | 0.571 |
| Captopril | 16 | 1 | 66 | 0.89 | -0.53 | I | 0.367 |
| Carbachol | 10 | 2 | 42 | 0.24 | -1.30 | I | 0.213 |
| Carbadox | 29 | 4 | 94 | 1.14 | 0.09 | A | 0.516 |
| Carbenicillin | 35 | 2 | 142 | 2.28 | -0.08 | I | 0.471 |
| Carbenoxolone | 50 | 0 | 242 | 6.17 | 1.32 | A | 0.778 |
| Carbidopa | 20 | 2 | 78 | 0.93 | -0.65 | I | 0.338 |
| Carbinoxamine | 27 | 2 | 96 | 0.57 | -0.64 | I | 0.339 |
| Carisoprodol | 19 | 2 | 78 | 0.96 | -0.88 | I | 0.290 |
| Carprofen | 28 | 1 | 104 | 0.98 | -0.23 | I | 0.437 |
| Carvedilol | 42 | 2 | 154 | 1.17 | -0.49 | I | 0.371 |
| Cefaclor | 33 | 3 | 128 | 1.72 | -0.49 | I | 0.373 |
| Cefadroxil | 34 | 3 | 134 | 1.91 | -0.46 | I | 0.379 |
| Cefamandole | 43 | 6 | 166 | 1.96 | -1.39 | I | 0.193 |
| Cefdinir | 36 | 5 | 138 | 2.01 | -0.72 | I | 0.320 |
| Cefditorin | 55 | 6 | 216 | 2.37 | -1.61 | I | 0.159 |
| Cefoperazone | 60 | 9 | 236 | 3.28 | -1.67 | I | 0.150 |
| Cefotaxime | 40 | 5 | 156 | 2.17 | -0.87 | I | 0.288 |
| Cefoxitin | 37 | 3 | 148 | 2.31 | -0.28 | I | 0.421 |
| Cefpodoxime | 37 | 5 | 146 | 2.09 | -0.97 | I | 0.267 |
| Cefprozil | 37 | 3 | 142 | 2.11 | -0.07 | I | 0.474 |
| Cefsulodin | 52 | 4 | 194 | 2.66 | 0.19 | A | 0.535 |
| Ceftazidime | 51 | 6 | 198 | 2.32 | -1.34 | I | 0.200 |
| Ceftibuten | 37 | 4 | 142 | 1.66 | -0.88 | I | 0.286 |
| Ceftriaxone | 49 | 8 | 192 | 2.73 | -1.50 | I | 0.175 |
| Cefuroxime | 39 | 4 | 150 | 2.04 | -0.52 | I | 0.363 |
| Cephalexin | 33 | 3 | 128 | 1.72 | -0.49 | I | 0.373 |
| Cephalothin | 35 | 2 | 136 | 1.84 | -0.14 | I | 0.455 |
| Cephradine | 32 | 3 | 128 | 1.72 | -0.74 | I | 0.316 |
| Cetirizine | 36 | 2 | 134 | 1.08 | -0.62 | I | 0.341 |
| Cetylpyridinium | 25 | 1 | 90 | 0.15 | -0.90 | I | 0.285 |
| Chenodiol | 32 | 0 | 160 | 3.07 | -0.69 | I | 0.324 |
| Chlorcyclizine | 29 | 2 | 108 | 0.96 | -0.59 | I | 0.350 |
| Chlorhexidine | 45 | 10 | 158 | 1.24 | -2.28 | I | 0.090 |
| Chlormadinoneacetate | 36 | 0 | 164 | 3.64 | 0.68 | A | 0.653 |
| Chlorocresol | 12 | 0 | 42 | 0.41 | 0.00 | I | 0.496 |
| Chloroguanide | 22 | 5 | 78 | 0.58 | -1.43 | I | 0.191 |
| Chloroquine | 28 | 3 | 106 | 0.99 | -0.95 | I | 0.273 |
| Chlorothiazide | 26 | 3 | 94 | 1.41 | -0.07 | I | 0.477 |
| Chlorotrianisene | 39 | 0 | 136 | 1.75 | 1.35 | A | 0.788 |
| Chloroxine | 19 | 1 | 68 | 0.94 | 0.14 | A | 0.531 |
| Chloroxylenol | 13 | 0 | 48 | 0.52 | -0.07 | I | 0.479 |
| Chlorpheniramine | 26 | 2 | 92 | 0.57 | -0.60 | I | 0.350 |
| Chlorpromazine | 29 | 2 | 110 | 1.39 | -0.24 | I | 0.434 |
| Chlorpropamide | 23 | 2 | 80 | 0.82 | -0.17 | I | 0.453 |
| Chlorprothixene | 30 | 1 | 110 | 1.39 | 0.31 | A | 0.570 |
| Chlortetracycline | 44 | 2 | 192 | 5.56 | 2.26 | A | 0.901 |
| Chlorthalidone | 33 | 2 | 124 | 1.36 | -0.31 | I | 0.416 |
| Chlorzoxazone | 16 | 1 | 58 | 0.45 | -0.44 | I | 0.388 |
| Cholesterol | 32 | 0 | 158 | 2.57 | -1.13 | I | 0.236 |
| Choline | 6 | 1 | 28 | 0 | -1.25 | I | 0.222 |
| Cilostazol | 36 | 5 | 140 | 1.19 | -1.81 | I | 0.136 |
| Cimetidine | 22 | 6 | 74 | 0.37 | -1.67 | I | 0.156 |
| Cinnarizine | 41 | 2 | 142 | 0.8 | -0.28 | I | 0.422 |
| Ciprofloxacin | 33 | 3 | 134 | 1.87 | -0.76 | I | 0.311 |
| Citalopram | 34 | 2 | 126 | 1.13 | -0.47 | I | 0.376 |
| Clarithromycin | 56 | 1 | 274 | 5.36 | -0.79 | I | 0.297 |
| Clavulanate | 18 | 1 | 74 | 1.22 | -0.24 | I | 0.435 |
| Clidinium | 36 | 1 | 144 | 1.5 | -0.59 | I | 0.348 |
| Clindamycin | 29 | 2 | 136 | 2.33 | -1.10 | I | 0.243 |
| Clofazimine | 50 | 4 | 180 | 2.59 | 0.65 | A | 0.647 |
| Clomiphenecitrate | 41 | 1 | 142 | 1.28 | 0.58 | A | 0.633 |
| Clomipramine | 30 | 2 | 114 | 1.26 | -0.43 | I | 0.386 |
| Clonidine | 19 | 3 | 70 | 0.73 | -0.84 | I | 0.299 |
| Clopidogrel | 29 | 1 | 110 | 1.31 | -0.04 | I | 0.483 |
| Clopidol | 14 | 1 | 54 | 0.72 | -0.34 | I | 0.413 |
| Clorsulon | 28 | 3 | 104 | 1.88 | 0.24 | A | 0.553 |
| Cloxacillin | 40 | 3 | 164 | 2.69 | -0.28 | I | 0.420 |
| Cloxyquin | 18 | 1 | 62 | 0.67 | 0.02 | A | 0.502 |
| Clozapine | 33 | 4 | 126 | 1.64 | -0.72 | I | 0.321 |
| Colforsin | 34 | 0 | 170 | 5.66 | 2.07 | A | 0.883 |
| Colistimethate | 128 | 16 | 510 | 6.96 | -2.72 | I | 0.054 |
| Colistin | 92 | 16 | 376 | 6.08 | -2.84 | I | 0.051 |
| Cortisone | 33 | 0 | 154 | 3.16 | 0.11 | A | 0.517 |
| Cotinine | 18 | 2 | 66 | 0.44 | -0.83 | I | 0.300 |
| Cromolyn | 49 | 0 | 180 | 2.68 | 1.67 | A | 0.835 |
| Crotamiton | 20 | 1 | 68 | 0.78 | 0.21 | A | 0.549 |
| Cyclizine | 28 | 2 | 102 | 0.76 | -0.62 | I | 0.345 |
| Cyclobenzaprine | 30 | 1 | 102 | 0.99 | 0.44 | A | 0.603 |
| Cyclopentolate | 26 | 1 | 104 | 0.9 | -0.82 | I | 0.299 |
| Cycloserine | 8 | 2 | 32 | 0 | -1.33 | I | 0.207 |
| Cyclosporine | 97 | 11 | 412 | 8 | -0.58 | I | 0.334 |
| Cyclothiazide | 35 | 3 | 142 | 2.04 | -0.65 | I | 0.334 |
| Cyproheptadine | 33 | 1 | 120 | 1.44 | 0.38 | A | 0.586 |
| Cyproterone | 34 | 0 | 164 | 3.08 | -0.47 | I | 0.373 |
| Cysteamine | 3 | 1 | 10 | 0 | -0.66 | I | 0.340 |
| Cysteine | 7 | 1 | 26 | 0 | -0.85 | I | 0.299 |
| Dactinomycin | 115 | 12 | 480 | 8.44 | -0.90 | I | 0.264 |
| Danazol | 34 | 1 | 154 | 2.55 | -0.64 | I | 0.337 |
| Dantrolene | 34 | 4 | 120 | 0.88 | -0.89 | I | 0.285 |
| Dapsone | 26 | 2 | 88 | 0.84 | 0.02 | A | 0.499 |
| Daunorubicin | 51 | 1 | 216 | 4.79 | 1.63 | A | 0.828 |
| Debrisoquinsulfate | 18 | 3 | 66 | 0.72 | -0.81 | I | 0.306 |
| Decoquinate | 37 | 1 | 140 | 1.65 | 0.14 | A | 0.525 |
| Deferoxamine | 43 | 6 | 166 | 1.2 | -2.27 | I | 0.090 |
| Dehydrocholic acid | 36 | 0 | 166 | 3.71 | 0.61 | A | 0.637 |
| Demeclocycline | 43 | 2 | 184 | 5.04 | 1.99 | A | 0.875 |
| Denatoniumbenzoate | 32 | 2 | 118 | 1.48 | 0.03 | A | 0.501 |
| Desipramine | 28 | 2 | 102 | 0.99 | -0.36 | I | 0.406 |
| Desonide | 38 | 0 | 184 | 4.34 | 0.50 | A | 0.610 |
| Desoxycorticosterone | 30 | 0 | 140 | 2.49 | -0.38 | I | 0.397 |
| Dexchlorpheniramine | 26 | 2 | 92 | 0.57 | -0.60 | I | 0.350 |
| Dexlansoprazole | 35 | 3 | 132 | 1.64 | -0.37 | I | 0.402 |
| Dexpanthenol | 14 | 1 | 60 | 0.48 | -1.06 | I | 0.254 |
| Dextromethorphan | 26 | 1 | 116 | 1.74 | -0.75 | I | 0.314 |
| Diazoxide | 21 | 2 | 76 | 1.31 | 0.18 | A | 0.541 |
| Dibenzothiophene | 21 | 0 | 72 | 0.27 | -0.13 | I | 0.463 |
| Dicloxacillin | 41 | 3 | 170 | 2.84 | -0.31 | I | 0.413 |
| Dicumarol | 38 | 0 | 138 | 2.2 | 1.47 | A | 0.807 |
| Dicyclomine | 24 | 1 | 106 | 0.55 | -1.89 | I | 0.128 |
| Dienestrol | 29 | 0 | 98 | 1.16 | 0.98 | A | 0.723 |
| Diethylcarbamazine | 15 | 3 | 64 | 0.54 | -1.62 | I | 0.163 |
| Diethylstilbestrol | 28 | 0 | 98 | 1.16 | 0.73 | A | 0.669 |
| Diethyltoluamide | 18 | 1 | 64 | 0.54 | -0.27 | I | 0.428 |
| Diflorasone | 36 | 0 | 174 | 4.29 | 0.69 | A | 0.654 |
| Diflunisal | 26 | 0 | 92 | 0.99 | 0.47 | A | 0.609 |
| Digoxin | 64 | 0 | 320 | 5.97 | -1.21 | I | 0.214 |
| Dihydroergotamine | 60 | 5 | 258 | 4.71 | -0.49 | I | 0.364 |
| Dihydrostreptomycin | 44 | 7 | 210 | 3.95 | -2.42 | I | 0.078 |
| Diloxanide | 18 | 1 | 66 | 0.7 | -0.24 | I | 0.437 |
| Diltiazem | 39 | 2 | 148 | 2.23 | 0.43 | A | 0.597 |
| Dimenhydrinate | 26 | 1 | 90 | 0.38 | -0.38 | I | 0.402 |
| Diperodon | 39 | 3 | 140 | 1.01 | -0.68 | I | 0.329 |
| Diphenhydramine | 26 | 1 | 90 | 0.38 | -0.38 | I | 0.402 |
| Dipyridamole | 44 | 8 | 182 | 1.91 | -2.98 | I | 0.046 |
| Dipyrone | 29 | 3 | 110 | 1.49 | -0.41 | I | 0.392 |
| Dirithromycin | 62 | 2 | 306 | 5.74 | -1.51 | I | 0.168 |
| Disopyramide | 33 | 3 | 124 | 0.98 | -1.04 | I | 0.255 |
| Disulfiram | 17 | 2 | 66 | 0.32 | -1.23 | I | 0.224 |
| D-Lactitol | 23 | 0 | 110 | 1.76 | -0.75 | I | 0.313 |
| Dobutamine | 29 | 1 | 106 | 0.96 | -0.15 | I | 0.456 |
| Domperidone | 42 | 5 | 166 | 1.5 | -1.88 | I | 0.128 |
| Donepezil | 38 | 1 | 148 | 1.58 | -0.29 | I | 0.418 |
| Dopamine | 14 | 1 | 50 | 0.53 | -0.25 | I | 0.435 |
| Doxepin | 30 | 1 | 108 | 0.99 | -0.01 | I | 0.491 |
| Doxycycline | 43 | 2 | 184 | 5.4 | 2.41 | A | 0.914 |
| Doxylamine | 27 | 2 | 98 | 0.58 | -0.79 | I | 0.308 |
| Droperidol | 40 | 3 | 148 | 1.27 | -0.72 | I | 0.319 |
| Duloxetine | 30 | 1 | 106 | 0.81 | -0.07 | I | 0.476 |
| Econazole | 34 | 2 | 122 | 1.03 | -0.28 | I | 0.422 |
| Edoxudine | 22 | 2 | 92 | 1.26 | -0.82 | I | 0.301 |
| Edrophonium | 15 | 1 | 58 | 0.6 | -0.52 | I | 0.370 |
| Eletriptan | 39 | 2 | 146 | 1.13 | -0.70 | I | 0.324 |
| Enalapril | 34 | 2 | 130 | 1.52 | -0.32 | I | 0.413 |
| Enoxacin | 31 | 4 | 122 | 2.03 | -0.47 | I | 0.377 |
| Enrofloxacin | 35 | 3 | 144 | 2.1 | -0.74 | I | 0.316 |
| Epirubicin | 52 | 1 | 220 | 4.79 | 1.58 | A | 0.821 |
| Equilin | 28 | 0 | 118 | 1.65 | -0.20 | I | 0.441 |
| Ergocalciferol | 35 | 0 | 152 | 1.71 | -0.91 | I | 0.278 |
| Ergonovine | 33 | 3 | 134 | 1.92 | -0.70 | I | 0.325 |
| Ergotamine | 61 | 5 | 258 | 4.71 | -0.24 | I | 0.425 |
| Erythromycin | 55 | 1 | 270 | 5.2 | -0.93 | I | 0.269 |
| Escitalopram | 34 | 2 | 126 | 1.13 | -0.47 | I | 0.376 |
| Estriol | 27 | 0 | 124 | 1.81 | -0.72 | I | 0.320 |
| Estrone | 27 | 0 | 118 | 1.65 | -0.46 | I | 0.380 |
| Estropipate | 33 | 0 | 140 | 1.93 | -0.26 | I | 0.426 |
| Ethacrynic acid | 25 | 0 | 90 | 1.28 | 0.71 | A | 0.665 |
| Ethambutol | 13 | 2 | 54 | 0.24 | -1.43 | I | 0.190 |
| Ethamivan | 20 | 1 | 74 | 0.77 | -0.25 | I | 0.434 |
| Ethionamide | 15 | 2 | 50 | 0.5 | -0.32 | I | 0.417 |
| Ethosuximide | 12 | 1 | 50 | 0.21 | -1.14 | I | 0.240 |
| Ethoxzolamide | 23 | 2 | 84 | 1.18 | -0.05 | I | 0.482 |
| Ethynodioldiacetate | 36 | 0 | 158 | 2.58 | -0.10 | I | 0.464 |
| Etidronate | 12 | 0 | 56 | 0 | -1.53 | I | 0.175 |
| Etomidate | 25 | 2 | 88 | 0.92 | -0.15 | I | 0.458 |
| Eucatropine | 26 | 1 | 108 | 1.27 | -0.70 | I | 0.325 |
| Eugenol | 16 | 0 | 54 | 0.57 | 0.30 | A | 0.570 |
| Evansblue | 89 | 6 | 326 | 5.79 | 2.73 | A | 0.934 |
| Famciclovir | 30 | 5 | 112 | 1.09 | -1.35 | I | 0.201 |
| Famotidine | 26 | 7 | 94 | 0.61 | -2.17 | I | 0.101 |
| Fampridine | 11 | 3 | 36 | 0.22 | -0.91 | I | 0.286 |
| Febuxostat | 31 | 2 | 110 | 1.39 | 0.27 | A | 0.561 |
| Felbamate | 22 | 2 | 76 | 0.43 | -0.58 | I | 0.355 |
| Fenbendazole | 31 | 3 | 108 | 0.68 | -0.69 | I | 0.328 |
| Fenofibrate | 34 | 0 | 126 | 1.68 | 0.76 | A | 0.673 |
| Fenoterol | 29 | 1 | 108 | 1.32 | 0.12 | A | 0.523 |
| Fenretinide | 39 | 1 | 142 | 1.31 | 0.10 | A | 0.515 |
| Fenspiride | 25 | 2 | 100 | 0.85 | -1.13 | I | 0.240 |
| Finasteride | 33 | 2 | 158 | 2.65 | -1.37 | I | 0.196 |
| Florfenicol | 27 | 1 | 102 | 1.06 | -0.23 | I | 0.435 |
| Floxuridine | 21 | 2 | 88 | 1.22 | -0.82 | I | 0.301 |
| Fluconazole | 31 | 6 | 116 | 1.08 | -1.70 | I | 0.150 |
| Flucytosine | 12 | 3 | 42 | 0.41 | -0.88 | I | 0.291 |
| Fludarabine | 26 | 5 | 110 | 1.46 | -1.79 | I | 0.140 |
| Flufenamic | 37 | 3 | 148 | 2.64 | 0.10 | A | 0.514 |
| Flumazenil | 31 | 3 | 118 | 1.85 | -0.09 | I | 0.470 |
| Flumequine | 27 | 1 | 106 | 2.08 | 0.65 | A | 0.651 |
| Flumethasone | 36 | 0 | 174 | 4.29 | 0.69 | A | 0.654 |
| Flumethasonepivalate | 43 | 0 | 206 | 4.61 | 0.43 | A | 0.593 |
| Flunarizine | 43 | 2 | 154 | 1.18 | -0.23 | I | 0.434 |
| Flunisolide | 39 | 0 | 190 | 4.73 | 0.75 | A | 0.668 |
| Flunixin | 29 | 2 | 108 | 1.25 | -0.25 | I | 0.432 |
| Fluocinoloneacetonide | 40 | 0 | 198 | 5.31 | 1.08 | A | 0.736 |
| Fluocinonide | 44 | 0 | 212 | 5.47 | 1.23 | A | 0.764 |
| Fluorescein | 39 | 0 | 148 | 2.23 | 1.01 | A | 0.726 |
| Fluorometholone | 34 | 0 | 164 | 4.05 | 0.65 | A | 0.646 |
| Fluorouracil | 21 | 2 | 88 | 1.22 | -0.82 | I | 0.301 |
| Fluoxetine | 29 | 1 | 108 | 0.81 | -0.46 | I | 0.379 |
| Fluphenazine | 39 | 3 | 160 | 1.81 | -1.25 | I | 0.216 |
| Flurandrenolide | 38 | 0 | 190 | 4.73 | 0.50 | A | 0.610 |
| Flurothyl | 10 | 0 | 50 | 0.24 | -1.31 | I | 0.210 |
| Flutamide | 24 | 2 | 94 | 1.63 | -0.03 | I | 0.487 |
| Fluvastatin | 41 | 1 | 154 | 1.98 | 0.49 | A | 0.611 |
| Fluvoxamine | 26 | 2 | 100 | 0.58 | -1.19 | I | 0.229 |
| Folic acid | 45 | 7 | 162 | 2.16 | -0.64 | I | 0.336 |
| Foscarnet | 8 | 0 | 30 | 0 | -0.60 | I | 0.353 |
| Fosfomycincalcium | 9 | 0 | 42 | 0 | -1.25 | I | 0.222 |
| Furazolidone | 22 | 3 | 80 | 0.66 | -0.90 | I | 0.284 |
| Furosemide | 30 | 2 | 108 | 1.37 | 0.14 | A | 0.529 |
| Gabapentin | 13 | 1 | 56 | 0.62 | -0.85 | I | 0.296 |
| Galanthamine | 28 | 1 | 122 | 2.01 | -0.38 | I | 0.398 |
| Gallaminetriethiodide | 39 | 3 | 168 | 0.93 | -2.87 | I | 0.051 |
| Gatifloxacin | 36 | 3 | 150 | 2.56 | -0.40 | I | 0.392 |
| Gemfibrozil | 22 | 0 | 86 | 0.85 | -0.25 | I | 0.431 |
| Gemifloxacinmesylate | 38 | 5 | 154 | 2.42 | -0.94 | I | 0.273 |
| Gentamicin | 35 | 5 | 174 | 2.76 | -2.80 | I | 0.055 |
| Gluceptate | 15 | 0 | 66 | 0.72 | -0.69 | I | 0.331 |
| Gluconolactone | 13 | 0 | 58 | 0.92 | -0.37 | I | 0.405 |
| Glucosamine | 12 | 1 | 58 | 0.92 | -0.91 | I | 0.283 |
| Glycopyrrolate | 29 | 1 | 124 | 1.08 | -1.35 | I | 0.200 |
| Gramicidin | 175 | 20 | 684 | 8.87 | -2.79 | I | 0.049 |
| Griseofulvin | 32 | 0 | 132 | 3.14 | 1.48 | A | 0.809 |
| Guanabenzacetate | 19 | 4 | 64 | 0.53 | -0.91 | I | 0.285 |
| Guanethidine | 15 | 4 | 60 | 0.12 | -2.10 | I | 0.108 |
| Guanfacine | 20 | 3 | 70 | 0.85 | -0.44 | I | 0.388 |
| Halazone | 21 | 1 | 74 | 0.66 | -0.12 | I | 0.465 |
| Haloperidol | 35 | 1 | 134 | 1.02 | -0.65 | I | 0.335 |
| Hetacillin | 36 | 3 | 158 | 3 | -0.48 | I | 0.372 |
| Hexachlorophene | 28 | 0 | 110 | 1.83 | 0.60 | A | 0.640 |
| Hexylresorcinol | 17 | 0 | 62 | 0.53 | -0.10 | I | 0.472 |
| Histamine | 10 | 3 | 34 | 0.13 | -1.12 | I | 0.244 |
| Homatropine | 26 | 1 | 106 | 0.82 | -1.07 | I | 0.250 |
| Hydralazine | 18 | 4 | 60 | 0.48 | -0.93 | I | 0.281 |
| Hydrastine | 39 | 1 | 160 | 2.46 | 0.08 | A | 0.510 |
| Hydrochlorothiazide | 25 | 3 | 94 | 1.41 | -0.32 | I | 0.414 |
| Hydroflumethiazide | 28 | 3 | 112 | 2.41 | 0.25 | A | 0.554 |
| Hydroxyamphetamine | 14 | 1 | 50 | 0.5 | -0.29 | I | 0.426 |
| Hydroxyurea | 5 | 2 | 16 | 0 | -0.90 | I | 0.289 |
| Hydroxyzinepamoate | 78 | 2 | 288 | 3.59 | 1.41 | A | 0.792 |
| Hyoscyamine | 27 | 1 | 110 | 0.82 | -1.12 | I | 0.241 |
| Ifosfamide | 15 | 2 | 64 | 0.65 | -1.20 | I | 0.229 |
| Imipramine | 29 | 2 | 108 | 0.99 | -0.55 | I | 0.359 |
| Indapamide | 35 | 3 | 130 | 1.41 | -0.49 | I | 0.373 |
| Inositol | 12 | 0 | 60 | 0.91 | -0.78 | I | 0.310 |
| Iodipamide | 45 | 2 | 170 | 2.87 | 1.04 | A | 0.731 |
| Iodoquinol | 19 | 1 | 68 | 0.94 | 0.14 | A | 0.531 |
| Irbesartan | 46 | 6 | 174 | 1.61 | -1.63 | I | 0.158 |
| Isoetharinemesylate | 20 | 1 | 80 | 1.05 | -0.37 | I | 0.403 |
| Isoflupredoneacetate | 38 | 0 | 176 | 3.9 | 0.59 | A | 0.632 |
| Isopropamide | 34 | 2 | 132 | 1.22 | -0.82 | I | 0.299 |
| Isosorbide | 11 | 0 | 54 | 0.33 | -1.25 | I | 0.219 |
| Isosorbidemononitrate | 15 | 1 | 68 | 0.57 | -1.30 | I | 0.211 |
| Isotretinon | 28 | 0 | 104 | 0.88 | -0.04 | I | 0.482 |
| Isoxsuprine | 29 | 1 | 106 | 1.01 | -0.08 | I | 0.473 |
| Itraconazole | 68 | 8 | 268 | 2.35 | -2.83 | I | 0.052 |
| Josamycin | 66 | 1 | 290 | 4.87 | -0.01 | I | 0.477 |
| Kanamycinasulfate | 35 | 4 | 174 | 3.11 | -2.11 | I | 0.104 |
| Ketoconazole | 49 | 4 | 194 | 1.62 | -1.77 | I | 0.139 |
| Ketoprofen | 28 | 0 | 94 | 0.73 | 0.53 | A | 0.624 |
| Ketorolactromethamine | 28 | 1 | 102 | 1.05 | 0.00 | A | 0.494 |
| Ketotifen | 32 | 1 | 122 | 1.7 | 0.28 | A | 0.562 |
| Labetalol | 32 | 2 | 116 | 1.12 | -0.23 | I | 0.435 |
| Lactulose | 24 | 0 | 120 | 1.85 | -1.15 | I | 0.235 |
| Lamotrigine | 23 | 5 | 82 | 1.07 | -0.91 | I | 0.283 |
| Lansoprazole | 35 | 3 | 132 | 1.64 | -0.37 | I | 0.402 |
| Leucovorin | 46 | 7 | 172 | 2.43 | -0.82 | I | 0.296 |
| Levalbuterol | 20 | 1 | 82 | 1.01 | -0.57 | I | 0.357 |
| Levamisole | 20 | 2 | 76 | 0.4 | -1.12 | I | 0.242 |
| Levobunolol | 26 | 1 | 106 | 1.19 | -0.64 | I | 0.338 |
| Levocetirizine | 36 | 2 | 134 | 1.08 | -0.62 | I | 0.341 |
| Levodopa | 18 | 1 | 66 | 0.77 | -0.16 | I | 0.457 |
| Levofloxacin | 35 | 3 | 146 | 2.78 | -0.10 | I | 0.466 |
| Levonordefrin | 16 | 1 | 62 | 0.73 | -0.41 | I | 0.395 |
| Levothyroxine | 32 | 1 | 122 | 1.81 | 0.40 | A | 0.591 |
| Lidocaine | 21 | 2 | 78 | 1.01 | -0.31 | I | 0.419 |
| Lindane | 12 | 0 | 60 | 0.91 | -0.78 | I | 0.310 |
| Liothyronine | 31 | 1 | 116 | 1.7 | 0.47 | A | 0.608 |
| Lisinopril | 36 | 3 | 138 | 1.6 | -0.61 | I | 0.345 |
| Lithiumcitrate | 15 | 0 | 58 | 0.8 | 0.01 | A | 0.498 |
| Lobendazole | 21 | 3 | 74 | 0.38 | -1.03 | I | 0.260 |
| Loperamide | 47 | 2 | 180 | 1.93 | -0.29 | I | 0.417 |
| Loratadine | 38 | 2 | 144 | 1.79 | -0.03 | I | 0.482 |
| Losartan | 43 | 6 | 156 | 1.45 | -1.23 | I | 0.220 |
| Lovastatin | 35 | 0 | 150 | 1.98 | -0.44 | I | 0.381 |
| Loxapinesuccinate | 33 | 3 | 126 | 1.72 | -0.33 | I | 0.410 |
| Malathion | 21 | 0 | 82 | 1.04 | 0.01 | A | 0.497 |
| Maprotiline | 30 | 1 | 118 | 1.43 | -0.24 | I | 0.432 |
| Mebendazole | 33 | 3 | 114 | 0.84 | -0.45 | I | 0.384 |
| Mebeverine | 39 | 1 | 146 | 1.68 | 0.23 | A | 0.547 |
| Mecamylamine | 13 | 1 | 70 | 0.61 | -1.92 | I | 0.126 |
| Mechlorethamine | 7 | 1 | 28 | 0.08 | -0.90 | I | 0.287 |
| Meclocycline | 45 | 2 | 190 | 5.71 | 2.83 | A | 0.941 |
| Meclofenamate | 27 | 1 | 96 | 1.23 | 0.41 | A | 0.595 |
| Medroxyprogesteroneacetate | 35 | 0 | 164 | 3.64 | 0.42 | A | 0.593 |
| Medrysone | 31 | 0 | 148 | 3.27 | 0.18 | A | 0.535 |
| Mefenamic acid | 26 | 1 | 90 | 0.96 | 0.29 | A | 0.566 |
| Mefexamide | 24 | 2 | 88 | 0.62 | -0.75 | I | 0.316 |
| Mefloquine | 33 | 2 | 142 | 2.03 | -0.88 | I | 0.285 |
| Melengestrolacetate | 38 | 0 | 170 | 3.96 | 1.11 | A | 0.743 |
| Meloxicam | 34 | 3 | 126 | 2.18 | 0.45 | A | 0.604 |
| Menadione | 20 | 0 | 68 | 0.94 | 0.69 | A | 0.661 |
| Menthol | 11 | 0 | 52 | 0.49 | -0.92 | I | 0.283 |
| Mepenzolate | 34 | 1 | 132 | 1.43 | -0.28 | I | 0.422 |
| Mephenterminesulfate | 15 | 1 | 56 | 0.47 | -0.52 | I | 0.370 |
| Mepivacaine | 23 | 2 | 90 | 1 | -0.72 | I | 0.323 |
| Mesna | 8 | 0 | 28 | 0 | -0.45 | I | 0.388 |
| Metaproterenol | 18 | 1 | 70 | 0.89 | -0.32 | I | 0.417 |
| Metaraminol | 15 | 1 | 56 | 0.54 | -0.43 | I | 0.390 |
| Metformin | 10 | 5 | 36 | 0.32 | -1.63 | I | 0.163 |
| Methacholine | 11 | 1 | 48 | 0.24 | -1.20 | I | 0.229 |
| Methacycline | 44 | 2 | 184 | 5.4 | 2.66 | A | 0.932 |
| Methapyrilene | 24 | 3 | 86 | 0.47 | -1.07 | I | 0.251 |
| Methazolamid | 19 | 4 | 70 | 1.18 | -0.61 | I | 0.348 |
| Methenamine | 12 | 4 | 60 | 0 | -3.00 | I | 0.047 |
| Methicillin | 34 | 2 | 142 | 2.51 | -0.07 | I | 0.472 |
| Methimazole | 9 | 2 | 32 | 0 | -1.08 | I | 0.252 |
| Methoxamine | 18 | 1 | 70 | 1.05 | -0.13 | I | 0.463 |
| Methoxsalen | 24 | 0 | 88 | 1.29 | 0.61 | A | 0.642 |
| Methscopolamine | 30 | 1 | 132 | 1.35 | -1.39 | I | 0.194 |
| Methylatropine | 28 | 1 | 118 | 1.01 | -1.24 | I | 0.219 |
| Methyldopa | 19 | 1 | 74 | 0.93 | -0.32 | I | 0.417 |
| Methylergonovine | 34 | 3 | 138 | 2 | -0.65 | I | 0.335 |
| Methylphenidate | 22 | 1 | 82 | 0.7 | -0.42 | I | 0.391 |
| Methylthiouracil | 12 | 2 | 42 | 0.33 | -0.68 | I | 0.334 |
| Methysergidemaleate | 35 | 3 | 144 | 2.04 | -0.80 | I | 0.302 |
| Metoclopramide | 24 | 3 | 92 | 1.2 | -0.67 | I | 0.334 |
| Metolazone | 35 | 3 | 132 | 2.34 | 0.45 | A | 0.602 |
| Metoprolol | 22 | 1 | 84 | 0.78 | -0.48 | I | 0.377 |
| Metronidazole | 15 | 3 | 56 | 0.78 | -0.75 | I | 0.318 |
| Mexiletine | 16 | 1 | 60 | 0.53 | -0.49 | I | 0.376 |
| Mianserin | 29 | 2 | 112 | 1.45 | -0.32 | I | 0.413 |
| Midodrine | 22 | 2 | 82 | 1.05 | -0.31 | I | 0.419 |
| Miglitol | 14 | 1 | 66 | 1.12 | -0.77 | I | 0.312 |
| Minaprine | 30 | 4 | 110 | 0.64 | -1.44 | I | 0.187 |
| Minocycline | 44 | 3 | 188 | 4.68 | 1.25 | A | 0.768 |
| Minoxidil | 20 | 5 | 76 | 0.8 | -1.54 | I | 0.174 |
| Mitomycin | 32 | 4 | 142 | 2.9 | -0.71 | I | 0.321 |
| Mitoxantrone | 42 | 4 | 160 | 2.84 | 0.41 | A | 0.590 |
| Molsidomine | 21 | 4 | 82 | 0.44 | -1.85 | I | 0.133 |
| Monensin | 52 | 0 | 264 | 5.13 | -1.03 | I | 0.250 |
| Montelukast | 58 | 1 | 220 | 2.29 | 0.22 | A | 0.540 |
| Morantelcitrate | 20 | 2 | 74 | 0.39 | -0.99 | I | 0.268 |
| Moxalactam | 49 | 6 | 194 | 2.86 | -0.92 | I | 0.275 |
| Moxifloxacin | 39 | 3 | 166 | 2.6 | -0.78 | I | 0.304 |
| Mycophenolic | 30 | 0 | 116 | 2.03 | 0.89 | A | 0.703 |
| Nadolol | 26 | 1 | 112 | 1.58 | -0.64 | I | 0.338 |
| Nafcillin | 40 | 2 | 162 | 2.76 | 0.24 | A | 0.550 |
| Nafronyl | 36 | 1 | 138 | 0.99 | -0.73 | I | 0.317 |
| Nalbuphine | 34 | 1 | 164 | 3.29 | -0.52 | I | 0.361 |
| Nalidixic acid | 24 | 2 | 88 | 1.65 | 0.44 | A | 0.603 |
| Naltrexone | 34 | 1 | 160 | 3.26 | -0.26 | I | 0.425 |
| Naproxol | 22 | 0 | 80 | 1.03 | 0.40 | A | 0.593 |
| Natamycin | 57 | 1 | 242 | 2.92 | -0.96 | I | 0.264 |
| Nateglinide | 29 | 1 | 112 | 1.17 | -0.35 | I | 0.407 |
| Nefopam | 27 | 1 | 98 | 0.77 | -0.27 | I | 0.427 |
| Neomycin | 45 | 6 | 224 | 3.76 | -3.15 | I | 0.039 |
| Neostigmine | 20 | 2 | 78 | 0.88 | -0.72 | I | 0.324 |
| Netilmicin | 36 | 5 | 172 | 2.84 | -2.30 | I | 0.087 |
| Niacin | 13 | 1 | 40 | 0.19 | -0.15 | I | 0.460 |
| Niacinamide | 13 | 2 | 40 | 0.19 | -0.44 | I | 0.389 |
| Nicardipine | 48 | 3 | 176 | 2.61 | 0.76 | A | 0.671 |
| Nicergoline | 43 | 3 | 176 | 2.75 | -0.35 | I | 0.402 |
| Niclosamide | 30 | 2 | 106 | 1.59 | 0.54 | A | 0.627 |
| Nicotineditartrate | 16 | 2 | 60 | 0.28 | -1.08 | I | 0.252 |
| Nicotinylalcoholtartrate | 11 | 1 | 34 | 0.15 | -0.25 | I | 0.435 |
| Nilutamide | 29 | 3 | 120 | 1.84 | -0.76 | I | 0.313 |
| Nimodipine | 39 | 2 | 148 | 2.38 | 0.60 | A | 0.636 |
| Nisoldipine | 37 | 2 | 140 | 2.46 | 0.78 | A | 0.678 |
| Nitrendipine | 35 | 2 | 130 | 2.3 | 0.84 | A | 0.691 |
| Nitrofurantoin | 24 | 4 | 86 | 0.74 | -1.05 | I | 0.256 |
| Nitromide | 21 | 3 | 72 | 1.05 | -0.10 | I | 0.470 |
| Nizatidine | 25 | 5 | 94 | 0.69 | -1.75 | I | 0.146 |
| Nomifensine | 26 | 2 | 96 | 1.21 | -0.16 | I | 0.454 |
| Norepinephrine | 15 | 1 | 56 | 0.65 | -0.31 | I | 0.421 |
| Norethindrone | 29 | 0 | 130 | 1.9 | -0.57 | I | 0.354 |
| Norethynodrel | 29 | 0 | 130 | 1.9 | -0.57 | I | 0.354 |
| Norgestimate | 35 | 1 | 152 | 2.74 | 0.00 | I | 0.489 |
| Nortriptyline | 29 | 1 | 102 | 0.99 | 0.19 | A | 0.541 |
| Noscapine | 41 | 1 | 170 | 2.93 | 0.39 | A | 0.586 |
| Nylidrin | 29 | 1 | 106 | 1.01 | -0.08 | I | 0.473 |
| Nystatin | 75 | 1 | 318 | 3.79 | -1.08 | I | 0.238 |
| Octisalate | 22 | 0 | 80 | 0.62 | -0.07 | I | 0.477 |
| Ofloxacin | 35 | 3 | 146 | 2.78 | -0.10 | I | 0.466 |
| Omeprazole | 34 | 3 | 126 | 1.74 | -0.05 | I | 0.479 |
| Ornidazole | 17 | 3 | 66 | 0.93 | -0.82 | I | 0.303 |
| Orphenadrinecitrate | 27 | 1 | 96 | 0.73 | -0.17 | I | 0.453 |
| Oseltamivir | 25 | 2 | 102 | 1.64 | -0.36 | I | 0.405 |
| Oxacillin | 39 | 3 | 158 | 2.54 | -0.25 | I | 0.427 |
| Oxantel | 22 | 2 | 78 | 0.6 | -0.53 | I | 0.367 |
| Oxcarbazepine | 29 | 2 | 102 | 1.75 | 0.78 | A | 0.680 |
| Oxethazaine | 43 | 3 | 168 | 2.06 | -0.55 | I | 0.357 |
| Oxfendazole | 33 | 3 | 114 | 0.84 | -0.45 | I | 0.384 |
| Oxidopamine | 15 | 1 | 56 | 0.8 | -0.13 | I | 0.464 |
| Oxolinic acid | 27 | 1 | 104 | 1.8 | 0.47 | A | 0.608 |
| Oxybenzone | 25 | 0 | 84 | 0.96 | 0.79 | A | 0.682 |
| Oxymetazoline | 24 | 2 | 100 | 1.71 | -0.39 | I | 0.398 |
| Oxyphenbutazone | 34 | 2 | 124 | 2 | 0.69 | A | 0.658 |
| Oxyphencyclimine | 32 | 2 | 128 | 1.16 | -1.09 | I | 0.245 |
| Oxytetracycline | 44 | 2 | 192 | 5.92 | 2.68 | A | 0.932 |
| Pantoprazole | 36 | 3 | 134 | 1.75 | -0.13 | I | 0.459 |
| Papaverine | 35 | 1 | 128 | 1.87 | 0.79 | A | 0.680 |
| Parachlorophenol | 11 | 0 | 36 | 0.3 | 0.06 | A | 0.514 |
| Paramethadione | 13 | 1 | 56 | 0.29 | -1.24 | I | 0.222 |
| Pararosaniline | 34 | 3 | 114 | 1.15 | 0.16 | A | 0.533 |
| Pargyline | 17 | 1 | 52 | 0.31 | 0.11 | A | 0.523 |
| Paromomycin | 45 | 5 | 224 | 3.76 | -2.86 | I | 0.051 |
| Paroxetine | 33 | 1 | 128 | 0.89 | -0.86 | I | 0.291 |
| Penfluridol | 48 | 1 | 192 | 1.81 | -0.78 | I | 0.303 |
| Penicillamine | 9 | 1 | 40 | 0 | -1.39 | I | 0.198 |
| Penicilling | 31 | 2 | 126 | 2.08 | -0.13 | I | 0.460 |
| Penicillinv | 32 | 2 | 130 | 2.16 | -0.08 | I | 0.471 |
| Pentetic acid | 31 | 3 | 118 | 1.36 | -0.66 | I | 0.335 |
| Pentoxifylline | 26 | 4 | 102 | 1.75 | -0.56 | I | 0.357 |
| Pentylenetetrazol | 13 | 4 | 50 | 0.09 | -1.89 | I | 0.130 |
| Perhexiline | 22 | 1 | 100 | 0.69 | -1.78 | I | 0.141 |
| Perindoprilerbumine | 30 | 2 | 128 | 2.11 | -0.50 | I | 0.370 |
| Perphenazine | 36 | 3 | 142 | 1.61 | -0.89 | I | 0.283 |
| Phenacetin | 17 | 1 | 58 | 0.54 | -0.08 | I | 0.477 |
| Phenazopyridine | 24 | 5 | 78 | 0.6 | -0.89 | I | 0.287 |
| Phenformin | 20 | 5 | 66 | 0.23 | -1.44 | I | 0.189 |
| Phenindione | 27 | 0 | 92 | 0.94 | 0.66 | A | 0.655 |
| Pheniramine | 25 | 2 | 86 | 0.38 | -0.62 | I | 0.345 |
| Phenolphthalein | 36 | 0 | 124 | 1.47 | 1.16 | A | 0.756 |
| Phentermine | 14 | 1 | 52 | 0.47 | -0.47 | I | 0.381 |
| Phentolamine | 30 | 3 | 108 | 0.96 | -0.63 | I | 0.343 |
| Phenylephrine | 15 | 1 | 54 | 0.46 | -0.38 | I | 0.403 |
| Phenylethylalcohol | 12 | 0 | 38 | 0.15 | -0.01 | I | 0.496 |
| Phenylmercuricacetate | 15 | 0 | 48 | 0.31 | 0.19 | A | 0.544 |
| Phenytoin | 29 | 2 | 102 | 1 | -0.09 | I | 0.471 |
| Phthalylsulfathiazole | 41 | 3 | 140 | 1.6 | 0.51 | A | 0.617 |
| Physostigminesalicylate | 26 | 3 | 112 | 1.25 | -1.61 | I | 0.163 |
| Phytonadione | 41 | 0 | 158 | 1.73 | 0.19 | A | 0.536 |
| Pimozide | 48 | 3 | 182 | 1.57 | -0.89 | I | 0.281 |
| Pinacidil | 24 | 5 | 84 | 0.79 | -1.13 | I | 0.241 |
| Pindolol | 23 | 2 | 88 | 0.69 | -0.92 | I | 0.281 |
| Pioglitazone | 35 | 2 | 126 | 0.84 | -0.55 | I | 0.359 |
| Pipamperone | 34 | 3 | 138 | 1.08 | -1.71 | I | 0.148 |
| Piperacillin | 48 | 5 | 196 | 3.25 | -0.58 | I | 0.346 |
| Piperazine | 6 | 2 | 24 | 0 | -1.24 | I | 0.223 |
| Piperidolate | 33 | 1 | 120 | 1 | -0.13 | I | 0.461 |
| Pipobroman | 18 | 2 | 72 | 0.46 | -1.26 | I | 0.218 |
| Piracetam | 12 | 2 | 46 | 0.36 | -0.96 | I | 0.276 |
| Pirenperone | 41 | 3 | 154 | 1.82 | -0.28 | I | 0.421 |
| Podofilox | 41 | 0 | 170 | 2.99 | 0.74 | A | 0.668 |
| Polymyxinb | 99 | 16 | 394 | 6.15 | -2.33 | I | 0.081 |
| Pralidoxime | 14 | 2 | 44 | 0.34 | -0.31 | I | 0.420 |
| Pramoxine | 25 | 1 | 96 | 0.45 | -1.00 | I | 0.265 |
| Praziquantel | 31 | 2 | 126 | 1.42 | -0.89 | I | 0.284 |
| Prazosin | 39 | 5 | 150 | 2.03 | -0.83 | I | 0.296 |
| Prednisolone | 33 | 0 | 154 | 3.16 | 0.11 | A | 0.517 |
| Prednisone | 34 | 0 | 154 | 3.16 | 0.36 | A | 0.580 |
| Pregabalin | 11 | 1 | 44 | 0.48 | -0.62 | I | 0.347 |
| Pregnenolonesuccinate | 37 | 0 | 168 | 2.77 | -0.38 | I | 0.396 |
| Prilocaine | 20 | 2 | 72 | 0.82 | -0.33 | I | 0.413 |
| Primaquine | 25 | 3 | 92 | 1.03 | -0.62 | I | 0.345 |
| Probenecid | 25 | 1 | 90 | 1.06 | 0.16 | A | 0.534 |
| Probucol | 42 | 0 | 192 | 3.28 | -0.31 | I | 0.410 |
| Procainamide | 21 | 3 | 76 | 0.46 | -1.09 | I | 0.249 |
| Procaine | 21 | 2 | 76 | 0.46 | -0.80 | I | 0.307 |
| Procyclidine | 26 | 1 | 108 | 0.62 | -1.45 | I | 0.186 |
| Proglumide | 30 | 2 | 108 | 1.15 | -0.11 | I | 0.466 |
| Promethazine | 28 | 2 | 106 | 1.28 | -0.32 | I | 0.414 |
| Propafenone | 33 | 1 | 116 | 0.81 | -0.05 | I | 0.480 |
| Propofol | 16 | 0 | 62 | 0.65 | -0.21 | I | 0.444 |
| Propranolol | 25 | 1 | 92 | 0.8 | -0.30 | I | 0.420 |
| Propylthiouracil | 14 | 2 | 50 | 0.53 | -0.54 | I | 0.365 |
| Protryptyline | 29 | 1 | 102 | 0.99 | 0.19 | A | 0.541 |
| Puromycin | 45 | 7 | 180 | 2.41 | -1.70 | I | 0.149 |
| Pyrantel | 19 | 2 | 68 | 0.39 | -0.79 | I | 0.309 |
| Pyridostigmine | 17 | 2 | 60 | 0.58 | -0.47 | I | 0.381 |
| Pyridoxine | 15 | 1 | 56 | 0.84 | -0.08 | I | 0.475 |
| Pyrilamine | 28 | 3 | 100 | 0.81 | -0.70 | I | 0.326 |
| Pyrimethamine | 24 | 4 | 86 | 1.19 | -0.53 | I | 0.366 |
| Pyrithione | 11 | 1 | 36 | 0.22 | -0.32 | I | 0.418 |
| Quetiapine | 37 | 3 | 140 | 1.49 | -0.64 | I | 0.338 |
| Quinacrine | 37 | 3 | 142 | 1.86 | -0.35 | I | 0.404 |
| Quinidine | 33 | 2 | 132 | 1.67 | -0.55 | I | 0.359 |
| Quinine | 33 | 2 | 132 | 1.67 | -0.55 | I | 0.359 |
| Quipazine | 23 | 3 | 84 | 0.59 | -1.03 | I | 0.258 |
| Rabeprazole | 35 | 3 | 126 | 1.4 | -0.19 | I | 0.444 |
| Racephedrine | 15 | 1 | 54 | 0.35 | -0.51 | I | 0.373 |
| Raloxifene | 49 | 1 | 182 | 2.06 | 0.51 | A | 0.613 |
| Ramipril | 38 | 2 | 152 | 2.04 | -0.35 | I | 0.404 |
| Ranitidine | 25 | 4 | 94 | 0.69 | -1.46 | I | 0.186 |
| Ranolazine | 40 | 3 | 154 | 1.78 | -0.59 | I | 0.348 |
| Resorcinol | 11 | 0 | 36 | 0.22 | -0.03 | I | 0.490 |
| Retinylpalmitate | 45 | 0 | 168 | 0.96 | -0.43 | I | 0.383 |
| Ribavirin | 21 | 4 | 88 | 1.15 | -1.48 | I | 0.182 |
| Riboflavin | 36 | 4 | 144 | 2.4 | -0.42 | I | 0.388 |
| Rifampin | 75 | 4 | 314 | 6 | 0.91 | A | 0.695 |
| Risedronate | 22 | 1 | 88 | 1.11 | -0.40 | I | 0.396 |
| Ritanserin | 49 | 3 | 184 | 2.1 | -0.17 | I | 0.445 |
| Ritodrine | 28 | 1 | 102 | 0.88 | -0.19 | I | 0.447 |
| Rizatriptan | 28 | 5 | 104 | 0.69 | -1.73 | I | 0.147 |
| Rolitetracycline | 50 | 3 | 218 | 5.46 | 1.42 | A | 0.796 |
| Ronidazole | 18 | 4 | 66 | 0.7 | -1.12 | I | 0.243 |
| Rosiglitazone | 35 | 3 | 126 | 0.73 | -0.97 | I | 0.269 |
| Roxarsone | 19 | 1 | 70 | 0.85 | -0.11 | I | 0.469 |
| Roxithromycin | 62 | 2 | 298 | 5.44 | -1.26 | I | 0.207 |
| Saccharin | 19 | 1 | 66 | 0.28 | -0.47 | I | 0.380 |
| Salicin | 24 | 0 | 100 | 1.42 | -0.14 | I | 0.458 |
| Salicyl | 12 | 0 | 40 | 0.26 | -0.03 | I | 0.490 |
| Salicylanilide | 24 | 1 | 78 | 0.61 | 0.28 | A | 0.566 |
| Salsalate | 28 | 0 | 94 | 0.88 | 0.71 | A | 0.664 |
| Sanguinarines | 39 | 1 | 152 | 2.24 | 0.43 | A | 0.597 |
| Sarafloxacin | 40 | 3 | 152 | 2.21 | 0.07 | A | 0.507 |
| Scopolamine | 29 | 1 | 124 | 1.05 | -1.39 | I | 0.194 |
| Selegiline | 19 | 1 | 62 | 0.55 | 0.14 | A | 0.532 |
| Semustine | 18 | 3 | 72 | 0.82 | -1.13 | I | 0.240 |
| Sennosidea | 85 | 0 | 350 | 6.48 | 2.47 | A | 0.915 |
| Sertraline | 28 | 1 | 106 | 1.2 | -0.12 | I | 0.463 |
| Sibutramine | 23 | 1 | 98 | 1.55 | -0.39 | I | 0.398 |
| Sildenafilcitrate | 45 | 6 | 178 | 2.21 | -1.50 | I | 0.176 |
| Simvastatin | 36 | 0 | 158 | 2.14 | -0.60 | I | 0.344 |
| Sirolimus | 77 | 1 | 328 | 5.04 | 0.13 | A | 0.509 |
| Sisomicin | 34 | 5 | 164 | 2.64 | -2.44 | I | 0.077 |
| Sodium Phenylacetate | 14 | 0 | 44 | 0.31 | 0.24 | A | 0.556 |
| Sodium Salicylate | 14 | 0 | 46 | 0.3 | 0.08 | A | 0.516 |
| Sodium dehydrocholate | 36 | 0 | 166 | 3.71 | 0.61 | A | 0.637 |
| Sodium oxybate | 7 | 0 | 24 | 0.16 | -0.22 | I | 0.444 |
| Solifenacinsuccinate | 38 | 2 | 150 | 1.6 | -0.71 | I | 0.321 |
| Sorbitol | 11 | 0 | 50 | 0.48 | -0.78 | I | 0.311 |
| Spectinomycin | 26 | 2 | 128 | 2.86 | -0.65 | I | 0.335 |
| Spiperone | 40 | 3 | 154 | 1.37 | -1.06 | I | 0.249 |
| Spironolactone | 37 | 0 | 174 | 2.99 | -0.57 | I | 0.351 |
| Strychnine | 36 | 2 | 164 | 2.54 | -1.18 | I | 0.228 |
| Succinylsulfathiazole | 33 | 3 | 114 | 1.22 | -0.01 | I | 0.489 |
| Sulconazolenitrate | 34 | 2 | 122 | 1.03 | -0.28 | I | 0.422 |
| Sulfabenzamide | 29 | 2 | 96 | 0.93 | 0.28 | A | 0.564 |
| Sulfachlorpyridazine | 27 | 4 | 92 | 1.08 | -0.33 | I | 0.412 |
| Sulfadiazine | 26 | 4 | 86 | 0.89 | -0.36 | I | 0.406 |
| Sulfadoxine | 30 | 4 | 106 | 1.44 | -0.21 | I | 0.440 |
| Sulfamerazine | 27 | 4 | 92 | 1 | -0.43 | I | 0.390 |
| Sulfameter | 28 | 4 | 96 | 1.12 | -0.33 | I | 0.412 |
| Sulfamethazine | 28 | 4 | 98 | 1.12 | -0.49 | I | 0.374 |
| Sulfamethoxazole | 25 | 3 | 88 | 0.78 | -0.61 | I | 0.348 |
| Sulfamethoxypyridazine | 28 | 4 | 96 | 1.12 | -0.33 | I | 0.412 |
| Sulfamonomethoxine | 28 | 4 | 96 | 1.04 | -0.43 | I | 0.389 |
| Sulfanilate | 34 | 2 | 118 | 0.92 | -0.11 | I | 0.466 |
| Sulfapyridine | 26 | 3 | 86 | 0.89 | -0.07 | I | 0.478 |
| Sulfaquinoxaline | 33 | 4 | 112 | 1.26 | -0.11 | I | 0.466 |
| Sulfasalazine | 43 | 4 | 144 | 1.63 | 0.46 | A | 0.604 |
| Sulfathiazole | 24 | 3 | 82 | 0.78 | -0.41 | I | 0.394 |
| Sulfisoxazole | 26 | 3 | 94 | 0.94 | -0.62 | I | 0.345 |
| Suloctidil | 26 | 1 | 102 | 0.9 | -0.67 | I | 0.332 |
| Sulpiride | 30 | 3 | 116 | 1.3 | -0.83 | I | 0.298 |
| Sumatriptan | 27 | 3 | 102 | 0.97 | -0.92 | I | 0.280 |
| Suprofen | 26 | 0 | 90 | 0.78 | 0.38 | A | 0.588 |
| Suramin | 132 | 6 | 476 | 7.96 | 4.93 | N.C. | 0.992 |
| Tacrolimus | 67 | 1 | 292 | 4.99 | 0.23 | A | 0.537 |
| Tamoxifencitrate | 40 | 1 | 138 | 1.28 | 0.63 | A | 0.643 |
| Tannic | 172 | 0 | 658 | 11.9 | 7.73 | N.C. | 0.999 |
| Taurine | 8 | 1 | 28 | 0 | -0.74 | I | 0.321 |
| Teicoplanin | 8 | 1 | 28 | 0 | -0.74 | I | 0.321 |
| Telithromycin | 71 | 5 | 308 | 5.55 | -0.47 | I | 0.365 |
| Temefos | 36 | 0 | 134 | 1.4 | 0.34 | A | 0.576 |
| Tenoxicam | 33 | 3 | 120 | 2.3 | 0.79 | A | 0.681 |
| Terazosin | 37 | 5 | 150 | 2.03 | -1.34 | I | 0.201 |
| Terbinafine | 31 | 1 | 108 | 0.88 | 0.12 | A | 0.522 |
| Terbutalinehemisulfate | 19 | 1 | 78 | 1.05 | -0.48 | I | 0.378 |
| Terfenadine | 47 | 1 | 184 | 1.38 | -0.93 | I | 0.273 |
| Testosterone | 31 | 0 | 144 | 2.48 | -0.44 | I | 0.383 |
| Tetracaine | 23 | 2 | 84 | 0.5 | -0.84 | I | 0.297 |
| Tetrahydrozoline | 21 | 2 | 80 | 0.52 | -1.03 | I | 0.259 |
| Tetramizole | 20 | 2 | 76 | 0.4 | -1.12 | I | 0.242 |
| Theophylline | 18 | 4 | 70 | 1.19 | -0.85 | I | 0.297 |
| Thiabendazole | 22 | 3 | 76 | 0.6 | -0.68 | I | 0.333 |
| Thiamine | 24 | 4 | 90 | 0.98 | -1.07 | I | 0.251 |
| Thiamylal | 21 | 2 | 82 | 1.32 | -0.26 | I | 0.431 |
| Thimerosal | 17 | 0 | 58 | 0.5 | 0.17 | A | 0.538 |
| Thioguanine | 16 | 5 | 58 | 0.6 | -1.43 | I | 0.192 |
| Thiopental | 19 | 2 | 78 | 1.08 | -0.74 | I | 0.318 |
| Thioridazine | 34 | 2 | 134 | 1.53 | -0.60 | I | 0.346 |
| Thiothixene | 42 | 3 | 162 | 1.85 | -0.59 | I | 0.347 |
| Thiram | 13 | 2 | 50 | 0.32 | -1.04 | I | 0.258 |
| Thonzylamine | 28 | 4 | 100 | 0.81 | -0.99 | I | 0.265 |
| Tiapride | 28 | 2 | 104 | 1.13 | -0.34 | I | 0.409 |
| Ticarcillin | 33 | 2 | 138 | 2.16 | -0.43 | I | 0.386 |
| Ticlopidine | 24 | 1 | 90 | 0.75 | -0.46 | I | 0.382 |
| Tilmicosin | 68 | 2 | 310 | 5.16 | -0.96 | I | 0.261 |
| Tilorone | 39 | 2 | 150 | 0.98 | -1.18 | I | 0.229 |
| Tinidazole | 21 | 3 | 78 | 0.94 | -0.69 | I | 0.330 |
| Tioconazole | 32 | 2 | 118 | 0.81 | -0.74 | I | 0.316 |
| Tobramycin | 34 | 5 | 168 | 2.92 | -2.43 | I | 0.078 |
| Tolazamide | 28 | 3 | 104 | 0.87 | -0.94 | I | 0.276 |
| Tolazoline | 17 | 2 | 58 | 0.19 | -0.78 | I | 0.311 |
| Tolmetin | 27 | 1 | 96 | 1.15 | 0.31 | A | 0.572 |
| Tolnaftate | 33 | 1 | 114 | 1.22 | 0.57 | A | 0.632 |
| Toltrazuril | 40 | 3 | 154 | 2.35 | 0.08 | A | 0.510 |
| Topiramate | 26 | 1 | 130 | 2.16 | -1.31 | I | 0.206 |
| Toremiphene | 41 | 1 | 142 | 1.28 | 0.58 | A | 0.633 |
| Tramadol | 23 | 1 | 96 | 1.17 | -0.67 | I | 0.333 |
| Trandolapril | 39 | 2 | 156 | 2.18 | -0.23 | I | 0.433 |
| Tranylcypromine | 14 | 1 | 52 | 0.28 | -0.69 | I | 0.331 |
| Trazodone | 36 | 5 | 138 | 0.91 | -2.00 | I | 0.116 |
| Triacetin | 17 | 0 | 62 | 0.72 | 0.12 | A | 0.526 |
| Triamcinolone | 35 | 0 | 168 | 3.82 | 0.34 | A | 0.572 |
| Triamcinolone | 35 | 0 | 168 | 3.82 | 0.34 | A | 0.572 |
| Triamterene | 29 | 7 | 102 | 1.36 | -1.13 | I | 0.239 |
| Trichlormethiazide | 28 | 3 | 110 | 2.08 | 0.02 | A | 0.497 |
| Triclosan | 24 | 0 | 86 | 1.15 | 0.59 | A | 0.639 |
| Trientine | 9 | 4 | 34 | 0.08 | -1.72 | I | 0.151 |
| Trifluoperazine | 37 | 3 | 152 | 1.77 | -1.20 | I | 0.224 |
| Triflupromazine | 32 | 2 | 128 | 1.59 | -0.59 | I | 0.348 |
| Trifluridine | 24 | 2 | 106 | 1.42 | -1.17 | I | 0.231 |
| Trihexyphenidyl | 27 | 1 | 112 | 0.65 | -1.46 | I | 0.184 |
| Trilostane | 31 | 1 | 152 | 2.78 | -0.97 | I | 0.266 |
| Trimethadione | 12 | 1 | 52 | 0 | -1.53 | I | 0.177 |
| Trimethoprim | 28 | 4 | 104 | 1.54 | -0.45 | I | 0.382 |
| Trimetozine | 25 | 1 | 98 | 1.32 | -0.15 | I | 0.457 |
| Trimipramine | 30 | 2 | 114 | 1.15 | -0.56 | I | 0.356 |
| Trioxsalen | 25 | 0 | 96 | 1.64 | 0.67 | A | 0.656 |
| Tripelennaminecitrate | 26 | 3 | 90 | 0.58 | -0.73 | I | 0.321 |
| Triprolidine | 30 | 2 | 106 | 0.54 | -0.67 | I | 0.332 |
| Trisodiumethylenediaminetetra | 23 | 2 | 86 | 0.96 | -0.46 | I | 0.382 |
| Tropicamide | 29 | 2 | 100 | 0.98 | 0.04 | A | 0.504 |
| Tryptophan | 21 | 2 | 76 | 0.46 | -0.80 | I | 0.306 |
| Tuaminoheptane | 7 | 1 | 28 | 0.16 | -0.81 | I | 0.307 |
| Tubocurarine | 63 | 2 | 256 | 4.84 | 1.44 | A | 0.798 |
| Tylosintartrate | 72 | 1 | 328 | 5.83 | -0.23 | I | 0.420 |
| Tyloxapol | 18 | 0 | 76 | 0.74 | -0.65 | I | 0.339 |
| Tyrothricin | 117 | 11 | 444 | 6.17 | -0.03 | I | 0.465 |
| Ursodiol | 32 | 0 | 160 | 3.07 | -0.69 | I | 0.324 |
| Valacyclovir | 29 | 6 | 114 | 1.17 | -1.96 | I | 0.120 |
| Valproate | 10 | 0 | 38 | 0.4 | -0.23 | I | 0.441 |
| Vancomycin | 133 | 9 | 550 | 9.92 | 1.02 | A | 0.706 |
| Vardenafil | 46 | 6 | 182 | 2.25 | -1.50 | I | 0.176 |
| Venlafaxine | 24 | 1 | 100 | 1.36 | -0.50 | I | 0.372 |
| Verapamil | 42 | 2 | 160 | 2.03 | 0.05 | A | 0.503 |
| Vidarabine | 25 | 5 | 104 | 1.19 | -1.91 | I | 0.126 |
| Vigabatrin | 10 | 1 | 34 | 0.32 | -0.31 | I | 0.421 |
| Vinblastine | 78 | 4 | 350 | 8.03 | 1.32 | A | 0.773 |
| Warfarin | 34 | 0 | 120 | 1.89 | 1.44 | A | 0.804 |
| Xylazine | 20 | 2 | 74 | 0.84 | -0.46 | I | 0.383 |
| Yohimbine | 35 | 2 | 150 | 1.99 | -1.02 | I | 0.258 |
| Zidovudine | 25 | 5 | 96 | 1.42 | -1.04 | I | 0.257 |
| Zileuton | 22 | 2 | 80 | 0.91 | -0.33 | I | 0.414 |
| Zolmitriptan | 28 | 3 | 110 | 0.73 | -1.55 | I | 0.172 |
| Zomepirac | 28 | 1 | 102 | 1.35 | 0.36 | A | 0.582 |

DF: discriminant function value for each compound

CLASS: classification of the model for ach compound

P.(Activ): probability of a compounds for being active

SCBO: sum of conventional bond orders (H-depleted)

nN: number of Nitrogen atoms

ZM1: First Zagreb index M1

GGI4: topological charge index of order 4.
