# Supplementary material for: Novel Cancer Chemotherapy Hits by Molecular Topology: Dual Akt and Beta-Catenin Inhibitors
Source: PLoS One. 2015 Apr 24;10(4):e0124244. doi: 10.1371/journal.pone.0124244 (PMC4409212; doi:10.1371/journal.pone.0124244)
Supplement: S8 Table — (DOCX) [file pone.0124244.s008.docx]

**S8 Table. Compounds used in the *test set* and corresponding values of the DF_4_ to β-catenin inhibitors.**

| **COMPOUNDS** | **SCBO** | **nN** | **ZM1** | **GGI4** | **DF** | **CLASS** | **P. (Activ.)** |
| --- | --- | --- | --- | --- | --- | --- | --- |
| **ACTIVE GROUP** | | | | | | | |
| Cardamonin [194] | 29 | 0 | 98 | 1.196 | 1.02 | A | 0.730 |
| Celastrol [144] | 43 | 0 | 202 | 5.32 | 1.56 | A | 0.818 |
| Cisplatin [195] | 4 | 2 | 20 | 0 | -1.45 | I | 0.189 |
| Coumarin [196] | 29 | 0 | 112 | 1.529 | 0.36 | A | 0.581 |
| Deguelin [111] | 41 | 0 | 168 | 3.213 | 1.16 | A | 0.752 |
| Diclofenac [197] | 27 | 1 | 94 | 1.236 | 0.57 | A | 0.633 |
| Diosgenin [147] | 36 | 0 | 186 | 3.514 | -1.11 | I | 0.237 |
| EGCG [113] | 48 | 0 | 196 | 4.28 | 2.07 | A | 0.883 |
| Emodin [198] | 30 | 0 | 112 | 2.436 | 1.66 | A | 0.837 |
| Etodolac [199] | 28 | 1 | 114 | 1.823 | 0.00 | A | 0.493 |
| Fenbufen [200] | 28 | 0 | 92 | 0.493 | 0.40 | A | 0.594 |
| Flurbiprofen [200] | 26 | 0 | 90 | 0.844 | 0.45 | A | 0.605 |
| Hydrocortisone [201] | 32 | 0 | 154 | 3.157 | -0.14 | I | 0.453 |
| Indoprofen [202] | 31 | 1 | 112 | 1.169 | 0.16 | A | 0.532 |
| Mitotane [203] | 25 | 0 | 90 | 1.324 | 0.75 | A | 0.675 |
| Rofecoxib [204] | 34 | 0 | 118 | 1.193 | 0.79 | A | 0.681 |
| **INACTIVE GROUP** | | | | | | | |
| Acetazolamide | 18 | 4 | 64 | 0.775 | -0.88 | I | 0.290 |
| Adenine | 15 | 5 | 52 | 0.333 | -1.55 | I | 0.174 |
| Adrenalinebitartrate | 15 | 1 | 56 | 0.653 | -0.31 | I | 0.421 |
| Albendazole | 24 | 3 | 88 | 0.613 | -1.05 | I | 0.255 |
| Alendronate | 15 | 1 | 68 | 0.56 | -1.31 | I | 0.209 |
| Allantoin | 14 | 4 | 52 | 0.4 | -1.43 | I | 0.191 |
| Altretamine | 18 | 6 | 72 | 1.053 | -1.74 | I | 0.147 |
| Amifostine | 12 | 2 | 48 | 0.24 | -1.24 | I | 0.223 |
| Aminoglutethimide | 23 | 2 | 88 | 0.987 | -0.58 | I | 0.354 |
| Amoxapine | 32 | 3 | 120 | 1.527 | -0.36 | I | 0.404 |
| Amprolium | 25 | 4 | 88 | 1.076 | -0.55 | I | 0.361 |
| Anethole | 15 | 0 | 48 | 0.382 | 0.27 | A | 0.565 |
| Aspartame | 27 | 2 | 96 | 1.111 | -0.02 | I | 0.489 |
| Atomoxetine | 26 | 1 | 90 | 0.693 | -0.02 | I | 0.490 |
| Baclofen | 18 | 1 | 64 | 0.702 | -0.09 | I | 0.474 |
| Benserazide | 22 | 3 | 84 | 0.804 | -1.04 | I | 0.258 |
| Benzoxiquine | 30 | 1 | 98 | 0.747 | 0.46 | A | 0.608 |
| Bepridil | 35 | 2 | 132 | 0.832 | -1.01 | I | 0.260 |
| Beta-Carotene | 52 | 0 | 194 | 1.689 | 0.23 | A | 0.546 |
| Biperiden | 30 | 1 | 126 | 0.979 | -1.37 | I | 0.198 |
| Bisoprololfumarate | 26 | 1 | 102 | 0.862 | -0.72 | I | 0.322 |
| Bromocriptinemesylate | 57 | 5 | 256 | 5.491 | -0.20 | I | 0.433 |
| Bumetanide | 35 | 2 | 124 | 2.013 | 0.96 | A | 0.716 |
| Bupivacaine | 26 | 2 | 102 | 1.116 | -0.72 | I | 0.322 |
| Butamben | 18 | 1 | 62 | 0.462 | -0.22 | I | 0.443 |
| Butoconazole | 35 | 2 | 126 | 1.231 | -0.10 | I | 0.468 |
| Capsaicin | 27 | 1 | 98 | 0.893 | -0.13 | I | 0.461 |
| Carbamazepine | 28 | 2 | 96 | 1.379 | 0.55 | A | 0.627 |
| Carboplatin | 16 | 2 | 74 | 1.271 | -0.98 | I | 0.269 |
| Carmustine | 13 | 3 | 46 | 0.4 | -0.94 | I | 0.279 |
| Cefazolin | 40 | 8 | 156 | 1.884 | -2.07 | I | 0.108 |
| Cefmetazole | 40 | 7 | 156 | 2.321 | -1.27 | I | 0.212 |
| Cefonicid | 49 | 6 | 188 | 2.476 | -0.92 | I | 0.276 |
| Cephapirin | 38 | 3 | 144 | 2.036 | -0.05 | I | 0.480 |
| Chlorambucil | 23 | 1 | 84 | 0.502 | -0.55 | I | 0.361 |
| Chloramphenicol | 25 | 2 | 94 | 1.022 | -0.48 | I | 0.377 |
| Chlorophyllide | 64 | 4 | 248 | 3.53 | 0.20 | A | 0.533 |
| Cholecalciferol | 33 | 0 | 146 | 1.628 | -1.06 | I | 0.249 |
| Ciclopirox | 19 | 1 | 76 | 0.956 | -0.44 | I | 0.387 |
| Cinchonine | 31 | 2 | 122 | 1.36 | -0.66 | I | 0.333 |
| Cinoxacin | 27 | 2 | 104 | 1.795 | 0.17 | A | 0.537 |
| Citicoline | 37 | 4 | 162 | 1.988 | -2.00 | I | 0.115 |
| Clemastine | 32 | 1 | 124 | 0.796 | -0.92 | I | 0.278 |
| Clobetasolpropionate | 40 | 0 | 186 | 4.699 | 1.28 | A | 0.773 |
| Clofibrate | 20 | 0 | 76 | 0.982 | 0.14 | A | 0.529 |
| Clotrimazole | 39 | 2 | 134 | 1.443 | 0.56 | A | 0.629 |
| Coenzymeb12 | 142 | 18 | 620 | 12.488 | -1.60 | I | 0.147 |
| Colchicine | 39 | 1 | 150 | 2.791 | 1.22 | A | 0.765 |
| Cresol | 12 | 0 | 42 | 0.333 | -0.10 | I | 0.473 |
| Cycloheximide | 24 | 1 | 102 | 1.458 | -0.54 | I | 0.363 |
| Cyclophosphamide | 15 | 2 | 64 | 0.542 | -1.33 | I | 0.207 |
| Cytarabine | 21 | 3 | 88 | 1.188 | -1.15 | I | 0.237 |
| Danthron | 28 | 0 | 100 | 1.893 | 1.42 | A | 0.802 |
| Decamethonium | 17 | 2 | 78 | 0.4 | -2.03 | I | 0.114 |
| Deracoxib | 39 | 3 | 144 | 1.866 | 0.01 | A | 0.494 |
| Dexpropranolol | 25 | 1 | 92 | 0.796 | -0.30 | I | 0.420 |
| Dibucaine | 32 | 3 | 118 | 1.227 | -0.56 | I | 0.357 |
| Digitoxin | 63 | 0 | 314 | 5.33 | -1.75 | I | 0.137 |
| Dioxybenzone | 26 | 0 | 90 | 1.156 | 0.81 | A | 0.688 |
| Diphenylpyraline | 29 | 1 | 106 | 0.884 | -0.23 | I | 0.435 |
| Docosanol | 22 | 0 | 86 | 0.08 | -1.15 | I | 0.236 |
| Doxazosin | 46 | 5 | 180 | 2.507 | -0.75 | I | 0.311 |
| Doxorubicin | 52 | 1 | 220 | 4.787 | 1.58 | A | 0.821 |
| Dyclonine | 26 | 1 | 98 | 0.573 | -0.75 | I | 0.314 |
| Dyphylline | 23 | 4 | 94 | 1.632 | -0.87 | I | 0.291 |
| Emetine | 45 | 2 | 190 | 3.231 | -0.04 | I | 0.477 |
| Enilconazole | 26 | 2 | 92 | 0.768 | -0.37 | I | 0.403 |
| Erythrosine | 43 | 0 | 172 | 3.318 | 1.49 | A | 0.808 |
| Estradiol | 26 | 0 | 118 | 1.653 | -0.71 | I | 0.322 |
| Ethinylestradiol | 30 | 0 | 130 | 1.898 | -0.31 | I | 0.414 |
| Ethopropazine | 30 | 2 | 114 | 1.28 | -0.41 | I | 0.392 |
| Ethylparaben | 15 | 0 | 50 | 0.382 | 0.12 | A | 0.527 |
| Etoposide | 55 | 0 | 242 | 4.034 | 0.11 | A | 0.512 |
| Exemestane | 30 | 0 | 132 | 2.467 | 0.20 | A | 0.540 |
| Ezetimibe | 43 | 1 | 160 | 2.142 | 0.73 | A | 0.666 |
| Fexofenadine | 50 | 1 | 194 | 1.542 | -0.74 | I | 0.313 |
| Fipronil | 35 | 4 | 140 | 2.939 | 0.25 | A | 0.553 |
| Firocoxib | 32 | 0 | 128 | 1.312 | -0.33 | I | 0.409 |
| Fludrocortisone | 33 | 0 | 162 | 3.659 | 0.09 | A | 0.512 |
| Fomepizole | 8 | 2 | 26 | 0 | -0.88 | I | 0.291 |
| Fusidic acid | 44 | 0 | 208 | 4.775 | 0.73 | A | 0.662 |
| Glipizide | 43 | 5 | 156 | 1.236 | -1.19 | I | 0.227 |
| Guaifenesin | 17 | 0 | 62 | 0.462 | -0.18 | I | 0.452 |
| Halcinonide | 38 | 0 | 192 | 4.839 | 0.48 | A | 0.605 |
| Homosalate | 24 | 0 | 98 | 1.027 | -0.44 | I | 0.384 |
| Hycanthone | 34 | 2 | 128 | 1.862 | 0.23 | A | 0.549 |
| Hydroquinone | 11 | 0 | 36 | 0.302 | 0.06 | A | 0.514 |
| Hydroxychloroquine | 29 | 3 | 110 | 1.067 | -0.90 | I | 0.283 |
| Iopanic acid | 21 | 1 | 82 | 1.236 | -0.06 | I | 0.481 |
| Isoniazid | 14 | 3 | 44 | 0.191 | -0.78 | I | 0.312 |
| Isoproterenol | 18 | 1 | 70 | 0.813 | -0.41 | I | 0.395 |
| Isoxicam | 34 | 3 | 126 | 2.178 | 0.45 | A | 0.604 |
| Ketanserin | 41 | 3 | 154 | 1.822 | -0.28 | I | 0.421 |
| Levocarnitine | 11 | 1 | 48 | 0.24 | -1.20 | I | 0.229 |
| Lincomycin | 29 | 2 | 136 | 2.331 | -1.10 | I | 0.243 |
| Lomefloxacin | 33 | 3 | 134 | 2.613 | 0.10 | A | 0.517 |
| Mafenide | 17 | 2 | 58 | 0.462 | -0.46 | I | 0.383 |
| Meclizine | 40 | 2 | 146 | 1.258 | -0.30 | I | 0.417 |
| Megestrolacetate | 36 | 0 | 164 | 3.637 | 0.68 | A | 0.653 |
| Memantine | 15 | 1 | 84 | 1 | -2.00 | I | 0.116 |
| Mestranol | 31 | 0 | 134 | 1.938 | -0.31 | I | 0.414 |
| Metaxalone | 21 | 1 | 80 | 0.738 | -0.48 | I | 0.377 |
| Methocarbamol | 21 | 1 | 76 | 0.702 | -0.23 | I | 0.439 |
| Methotrexate | 46 | 8 | 168 | 2.28 | -0.99 | I | 0.263 |
| Methyleneblue | 29 | 3 | 108 | 1.591 | -0.15 | I | 0.457 |
| Methylprednisolone | 43 | 0 | 192 | 4.028 | 0.81 | A | 0.681 |
| Miconazolenitrate | 35 | 2 | 128 | 1.142 | -0.35 | I | 0.405 |
| Modafinil | 28 | 1 | 92 | 0.702 | 0.35 | A | 0.582 |
| Monobenzone | 22 | 0 | 72 | 0.493 | 0.38 | A | 0.589 |
| Mupirocin | 39 | 0 | 168 | 1.779 | -1.01 | I | 0.258 |
| Nadide | 58 | 7 | 242 | 3.151 | -2.19 | I | 0.095 |
| Naloxone | 33 | 1 | 148 | 3.259 | 0.39 | A | 0.586 |
| Naphazoline | 24 | 2 | 84 | 0.511 | -0.58 | I | 0.355 |
| Nifedipine | 34 | 2 | 126 | 2.298 | 0.88 | A | 0.701 |
| Nithiamide | 16 | 3 | 56 | 0.57 | -0.73 | I | 0.322 |
| Nitrofurazone | 19 | 4 | 64 | 0.535 | -0.90 | I | 0.286 |
| Nonoxyno | 22 | 0 | 80 | 0.382 | -0.35 | I | 0.408 |
| Norfloxacin | 31 | 3 | 122 | 2.031 | -0.18 | I | 0.448 |
| Norgestrel | 30 | 0 | 134 | 2.143 | -0.33 | I | 0.410 |
| Novobiocin | 58 | 2 | 234 | 4.48 | 1.41 | A | 0.794 |
| Olmesartan | 47 | 6 | 176 | 2.834 | -0.11 | I | 0.462 |
| Orlistat | 38 | 1 | 154 | 1.462 | -0.88 | I | 0.286 |
| Ouabain | 48 | 0 | 244 | 5.397 | -0.23 | I | 0.425 |
| Oxaprozin | 33 | 1 | 112 | 1.167 | 0.66 | A | 0.653 |
| Oxibendazole | 24 | 3 | 88 | 0.613 | -1.05 | I | 0.255 |
| Oxiconazolenitrate | 37 | 3 | 132 | 1.142 | -0.44 | I | 0.385 |
| Oxybutynin | 33 | 1 | 124 | 1.058 | -0.36 | I | 0.402 |
| Oxyquinoline | 17 | 1 | 56 | 0.436 | -0.05 | I | 0.484 |
| Pancuronium | 48 | 2 | 238 | 4.766 | -1.10 | I | 0.238 |
| Pefloxacine | 32 | 3 | 128 | 2.222 | -0.15 | I | 0.453 |
| Pergolidemesylate | 29 | 2 | 122 | 1.603 | -0.89 | I | 0.284 |
| Phenacemide | 18 | 2 | 58 | 0.311 | -0.38 | I | 0.402 |
| Phenelzine | 13 | 2 | 42 | 0.151 | -0.64 | I | 0.344 |
| Phenoxybenzamine | 28 | 1 | 98 | 0.782 | 0.00 | I | 0.493 |
| Phenyl | 25 | 1 | 84 | 0.804 | 0.31 | A | 0.571 |
| Phenylpropanolamine | 14 | 1 | 50 | 0.351 | -0.46 | I | 0.384 |
| Pilocarpine | 19 | 2 | 76 | 0.48 | -1.28 | I | 0.214 |
| Piperine | 29 | 1 | 106 | 0.599 | -0.56 | I | 0.357 |
| Pirenzepine | 37 | 5 | 140 | 2.092 | -0.52 | I | 0.365 |
| Pizotyline | 30 | 1 | 116 | 1.249 | -0.31 | I | 0.417 |
| Potassiump-Aminobenzoate | 14 | 1 | 46 | 0.382 | -0.12 | I | 0.466 |
| Pravastatin | 35 | 0 | 148 | 1.769 | -0.54 | I | 0.359 |
| Primidone | 22 | 2 | 82 | 0.813 | -0.58 | I | 0.353 |
| Proadifen | 34 | 1 | 124 | 1.058 | -0.11 | I | 0.464 |
| Prochlorperazine | 34 | 3 | 134 | 1.573 | -0.85 | I | 0.293 |
| Promazine | 28 | 2 | 104 | 1.12 | -0.35 | I | 0.406 |
| Propantheline | 36 | 1 | 142 | 1.8 | -0.09 | I | 0.467 |
| Proscillaridin | 47 | 0 | 224 | 3.974 | -0.64 | I | 0.332 |
| Pyrazinamide | 13 | 3 | 40 | 0.191 | -0.73 | I | 0.323 |
| Quinapril | 43 | 2 | 160 | 2.178 | 0.48 | A | 0.608 |
| Reserpine | 58 | 2 | 242 | 4.097 | 0.37 | A | 0.575 |
| Retinol | 26 | 0 | 98 | 0.804 | -0.20 | I | 0.445 |
| Rimantadine | 15 | 1 | 78 | 0.573 | -2.05 | I | 0.112 |
| Rolipram | 26 | 1 | 104 | 0.938 | -0.78 | I | 0.308 |
| Ropinirole | 24 | 2 | 92 | 0.773 | -0.87 | I | 0.290 |
| Rutoside | 55 | 0 | 236 | 4.044 | 0.58 | A | 0.625 |
| Salicylamide | 14 | 1 | 46 | 0.302 | -0.22 | I | 0.443 |
| Selamectin | 67 | 1 | 304 | 4.991 | -0.67 | I | 0.320 |
| Sodiumnitroprusside | 24 | 6 | 66 | 0 | -0.99 | I | 0.269 |
| Sparteinesulfate | 20 | 2 | 98 | 0.809 | -2.30 | I | 0.089 |
| Streptozosin | 20 | 3 | 86 | 1.436 | -0.96 | I | 0.272 |
| Sucralose | 24 | 0 | 120 | 1.971 | -1.00 | I | 0.262 |
| Sulfacetamide | 20 | 2 | 68 | 0.582 | -0.31 | I | 0.419 |
| Sulfadimethoxine | 30 | 4 | 106 | 1.276 | -0.40 | I | 0.395 |
| Sulfamethizole | 25 | 4 | 88 | 0.777 | -0.90 | I | 0.285 |
| Sulfanitran | 34 | 3 | 116 | 1.364 | 0.26 | A | 0.558 |
| Sulfinpyrazone | 44 | 2 | 152 | 1.838 | 0.94 | A | 0.712 |
| Tacrine | 22 | 2 | 82 | 1.08 | -0.28 | I | 0.427 |
| Tadalafil | 43 | 3 | 172 | 2.337 | -0.53 | I | 0.361 |
| Teniposide | 62 | 0 | 266 | 4.044 | 0.10 | A | 0.508 |
| Tetracycline | 43 | 2 | 186 | 5.173 | 2.00 | A | 0.876 |
| Tetroquinone | 16 | 0 | 60 | 0.907 | 0.23 | A | 0.554 |
| Thalidomide | 28 | 2 | 104 | 1.401 | -0.03 | I | 0.486 |
| Thiamphenicol | 27 | 1 | 102 | 1.062 | -0.23 | I | 0.435 |
| Thiotepa | 14 | 3 | 68 | 0 | -2.80 | I | 0.056 |
| Timolol | 24 | 4 | 104 | 0.886 | -2.23 | I | 0.095 |
| Tolbutamide | 24 | 2 | 84 | 0.822 | -0.22 | I | 0.441 |
| Tranexamic acid | 12 | 1 | 50 | 0.422 | -0.89 | I | 0.289 |
| Tranilast | 34 | 1 | 116 | 1.436 | 0.93 | A | 0.711 |
| Triamcinolonediacetate | 43 | 0 | 196 | 4.504 | 1.06 | A | 0.732 |
| Trichlorfon | 12 | 0 | 56 | 0.48 | -0.98 | I | 0.271 |
| Trimethobenzamide | 36 | 2 | 134 | 1.667 | 0.06 | A | 0.507 |
| Troleandomycin | 65 | 1 | 298 | 5.809 | 0.22 | A | 0.534 |
| Undecylenic | 11 | 1 | 42 | 0.08 | -0.94 | I | 0.280 |
| Urea | 4 | 2 | 12 | 0 | -0.85 | I | 0.299 |
| Valsartan | 44 | 5 | 160 | 1.844 | -0.53 | I | 0.362 |
| Vecuronium | 47 | 2 | 230 | 4.375 | -1.21 | I | 0.219 |
| Vesamicol | 24 | 1 | 98 | 0.476 | -1.38 | I | 0.198 |
| Xylometazoline | 23 | 2 | 94 | 1.519 | -0.41 | I | 0.392 |
| Zoxazolamine | 16 | 2 | 58 | 0.448 | -0.73 | I | 0.322 |

DF: discriminant function value for each compound

CLASS: classification of the model for ach compound

P.(Activ): probability of a compounds for being active

SCBO: sum of conventional bond orders (H-depleted)

nN: number of Nitrogen atoms

ZM1: First Zagreb index M1

GGI4: topological charge index of order 4.
