# Supplementary material for: Novel Cancer Chemotherapy Hits by Molecular Topology: Dual Akt and Beta-Catenin Inhibitors
Source: PLoS One. 2015 Apr 24;10(4):e0124244. doi: 10.1371/journal.pone.0124244 (PMC4409212; doi:10.1371/journal.pone.0124244)
Supplement: S9 Table — (DOCX) [file pone.0124244.s009.docx]

**S9 Table. Selected compounds as potential anti-cancer agents by the virtual screening of SPECS databases by applying DF_1_-_4_.**

| **COMPOUNDS** | **nR09** | **Wap** | **EEig11r** | **T(O..Br)** | **SRW08** | **MPC04** | **piPC02** | **piPC05** | **Dz** | **S2K** | **PCR** | **X2sol** | **JGI4** | **SCBO** | **nN** | **ZM1** | **GGI4** |
| --- | --- | --- | --- | --- | --- | --- | --- | --- | --- | --- | --- | --- | --- | --- | --- | --- | --- |
| Inhibitor nº1* | 0 | 20410 | 1.68 | 0 | 5788 | 85 | 4.248 | 6.009 | 55 | 5.102 | 1.458 | 11.045 | 0.054 | 39 | 2 | 138 | 2.556 |
| Inhibitor nº2 |  |  |  | 0 | 5252 | 76 | 4.143 | 5.7 |  |  |  |  |  | 38 | 2 | 134 | 2.302 |
| Inhibitor nº3 |  |  |  | 0 | 6276 | 94 | 4.29 | 6.021 |  |  |  |  |  | 40 | 2 | 148 | 2.716 |
| Inhibitor nº4 |  |  |  | 28 | 5012 | 70 | 4.078 | 5.485 |  |  |  |  |  | 34 | 1 | 128 | 1.726 |
| Inhibitor nº5 |  |  |  | 0 | 3542 | 52 | 3.85 | 5.124 |  |  |  |  |  | 25 | 0 | 94 | 1.6 |
| Inhibitor nº6 |  |  |  | 0 | 5504 | 88 | 4.22 | 5.635 |  |  |  |  |  | 39 | 2 | 146 | 1.901 |

*Selected after screening SPECS natural compounds database.
